# Annexin-A1 deficiency attenuates stress-induced tumor growth *via* fatty acid metabolism in mice: an Integrated multiple omics analysis on the stress- microbiome-metabolite-epigenetic-oncology (SMMEO) axis

**DOI:** 10.7150/thno.68611

**Published:** 2022-05-09

**Authors:** Jianzhou Cui, Karishma Sachaphibulkij, Wen Shiun Teo, Hong Meng Lim, Li Zou, Choon Nam Ong, Rudi Alberts, Jinmiao Chen, Lina H. K. Lim

**Affiliations:** 1 Immunology Translational Research Program, Yong Loo Lin School of Medicine, National University of Singapore (NUS), Singapore; 2 Department of Physiology, Yong Loo Lin School of Medicine, NUS, Singapore; 3 NUS Immunology Program, Life Sciences Institute, NUS, Singapore; 4 Saw Swee Hock School of Public Health, NUS, Singapore; 5 NUS Environmental Research Institute, NUS, Singapore; 6 Department of Microbiology and Immunology, Yong Loo Lin School of Medicine, NUS, Singapore; 7 Singapore Immunology Network (SIgN), Agency for Science, Technology and Research (A*STAR), Singapore

**Keywords:** 16S rRNA gene sequencing, gut microbiome, metabolic level, epigenetic signature, PICRUSt, differentially methylated regions, machine learning, restraint stress, serum metabolites, feces metabolites, WGBS, breast cancer, tumorigenesis

## Abstract

**Background:** High emotional or psychophysical stress levels have been correlated with an increased risk and progression of various diseases. How stress impacts the gut microbiota to influence metabolism and subsequent cancer progression is unclear.

**Methods:** Feces and serum samples from BALB/c ANXA1^+/+^ and ANXA1^-/-^ mice with or without chronic restraint stress were used for 16S rRNA gene sequencing and GC-MS metabolomics analysis to investigate the effect of stress on microbiome and metabolomics during stress and breast tumorigenesis. Breast tumors samples from stressed and non-stressed mice were used to perform Whole-Genome Bisulfite Sequencing (WGBS) and RNAseq analysis to construct the potential network from candidate hub genes. Finally, machine learning and integrated analysis were used to map the axis from chronic restraint stress to breast cancer development.

**Results:** We report that chronic stress promotes breast tumor growth *via* a stress-microbiome-metabolite-epigenetic-oncology (SMMEO) axis. Chronic restraint stress in mice alters the microbiome composition and fatty acids metabolism and induces an epigenetic signature in tumors xenografted after stress. Subsequent machine learning and systemic modeling analyses identified a significant correlation among microbiome composition, metabolites, and differentially methylated regions in stressed tumors. Moreover, silencing Annexin-A1 inhibits the changes in the gut microbiome and fatty acid metabolism after stress as well as basal and stress-induced tumor growth.

**Conclusions:** These data support a physiological axis linking the microbiome and metabolites to cancer epigenetics and inflammation. The identification of this axis could propel the next phase of experimental discovery in further understanding the underlying molecular mechanism of tumorigenesis caused by physiological stress.

## Introduction

Breast cancer, among women alone, accounts for nearly 30% of all cancer diagnoses, with an estimated 281,550 new cases and close to 43,600 deaths in 2020 1. Epidemiological and clinical studies have provided strong evidence for links between chronic stress and breast cancer progression 2. Stress is becoming an increasingly inevitable part of people's lives in contemporary societies. An integrated definition describes stress as a constellation of events that involves a stimulus that precipitates a reaction in the brain and, in turn, activates physiological fight-or-flight responses in the body^3^-^5^. Stress has been proposed to modulate the metabolic, transcriptional, and epigenetic regulation of various diseases, including cancers 6-9, and affects cancer progression through the suppression of immunity and the exacerbation of chronic inflammation 10-12.

Stress factors are also linked to the disruption of the commensal microbiome homeostasis. Given that the commensal microbiota is a host-intrinsic regulator of systemic immune functions 13, it is not surprising that stress alters microbiota diversity, *via* a process known as dysbiosis, influencing the systemic immune environment 14, which is associated with a poorer outcome in multiple diseases 15-17. Perturbations in the diversity of the microbiome can affect the abundance of specific bacterial species and increase the risk of disease 18-20.

Epigenetic changes in tumors are emerging as fundamental regulators of breast cancer development and progression 21. During carcinogenesis, chromatin structure alterations mainly include DNA chemical modification such as CpG methylation and post-translational modification of DNA bound proteins, including histones 22. Together with subclonal mutations and signals from the microenvironment, methylation modulates the cancer cell phenotype and affects the metastatic propensity of the tumor 23-25. The direct connections between metabolism and chromatin dynamics now present important conceptual challenges to explain many aspects of tumorigenesis 26. Changes to intracellular metabolism can alter the expression of specific histone methyltransferases and acetyltransferases, conferring widespread variations in epigenetic modification patterns 27, 28.

Direct connections between the microbiome, metabolism, and chromatin dynamics present important conceptual challenges to explain many aspects of tumorigenesis. Several components of the epigenetic machinery require intermediates of cellular metabolism for enzymatic function. Furthermore, changes to intracellular metabolism can alter the expression of specific histone methyltransferases and acetyltransferases, conferring widespread variations in epigenetic modification patterns.

Studies on one or two aspects of our analysis, such as microbiome, metabolism, or epigenetics in breast cancer, have been reported. However, systemic analysis of the effect of stress on breast cancer through multi-omic approaches has not been performed. The overall purpose of this study was to determine the links between stress and breast tumorigenesis *via* the gut microbiome, gut-serum metabolite levels, epigenetic signatures, and transcriptomic expression in tumors using integrated analysis and machine learning. Hereby, we hypothesize that stress hormones (e.g., cortisol) alert the gut microbiome composition *via* the brain-gut axis and causes changes in metabolites in the gut and blood. Stress will also further disturb epigenetic signatures and transcriptome profiling in mice tumors in a remote manner.

In particular, Annexin-A1 (ANXA1) is a glucocorticoid regulated gene which plays an essential role in breast cancer development both *in vitro* and *in vivo*. ANXA1 is implicated in multiple functions essential in cancer, including cell proliferation, apoptosis, chemosensitivity, metastasis, and invasion 55. ANXA1 associates with, and regulates NF-κB, and increases c-Myc activity to promote breast cancer migration and metastasis 56, 57. Importantly, ANXA1 deficient mice exhibit reduced tumor growth and enhanced survival 58. In this study, we hypothesized that ANXA1 is involved in stress-related promotion of breast cancer development and determined its role in the regulation of microbiome, gut-serum metabolites.

To our knowledge, our study suggests a novel stress-microbiome-metabolite-epigenetic-oncology (SMMEO) axis that is induced by stress.

## Results

### Stress promotes breast tumor development

**Figure 1:**
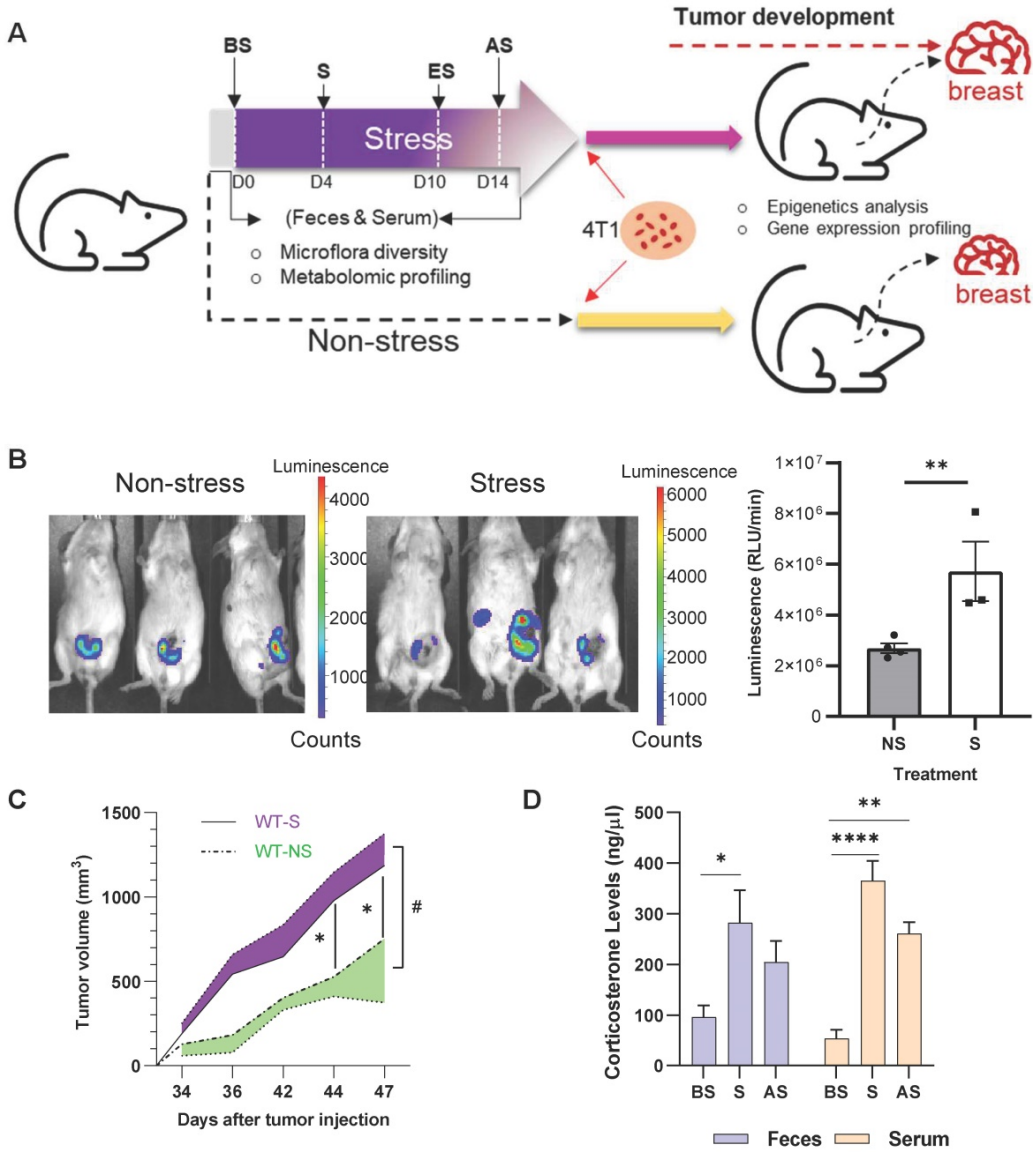
** Chronic stress promotes breast tumor development *in vivo* (A)** Schematic of experimental design and sample collection protocol for stress and non-stress; n ≥ 6 mice in each group. The treatment and sampling time were indicated with Before stress (BS), Stress (S), End of stress (ES), and After stress (AS) stages. **(B)** *In vivo* images of tumor samples by Xenogen IVIS imaging system in week seven after stress. The left mice were non-stressed; the right mice were stressed by restraining. 4T1 cells were injected after stress. The tumor area is shown in red. **(C)** Growth curve of tumors seven weeks after stress. ^#^*p* = 0.0216. **(D)** Fecal and serum corticosterone levels at different stages of acute restraint stress when compared with the non-stressed group. Data are representative of three independent experiments. *p < 0.05, **p < 0.01, ***p < 0.001 in one-way ANOVA with Tukey's post hoc test.

BALB/c mice were subjected to 10 days of restraint stress with one day of rest before orthotopic injection of murine 4T1-luc breast cancer cells into the mammary gland **(Figure 1A)**. Stressed mice exhibited higher levels of luciferase activity seven weeks after stress **(Figure 1B)**, and tumor size was markedly increased after stress from days 34 to 47 after injection **(Figure 1C)** (p < 0.05). Plasma and feces corticosterone levels were measured at day 0 (before stress, BS), 4 (during stress, S), and 14 (2 days after stress, AS) of the stress procedure **(Figure 1A)**. Stress significantly increased (p < 0.05) the plasma and feces corticosterone levels on days 4 and 14 **(Figure 1D)**. To further study the effect of stress on tumor development, multi-omics analyses were performed to investigate the underlying molecular mechanism in the process **(Figure S1)**.

### Stress alters gut microbiome composition in mice

**Figure 2:**
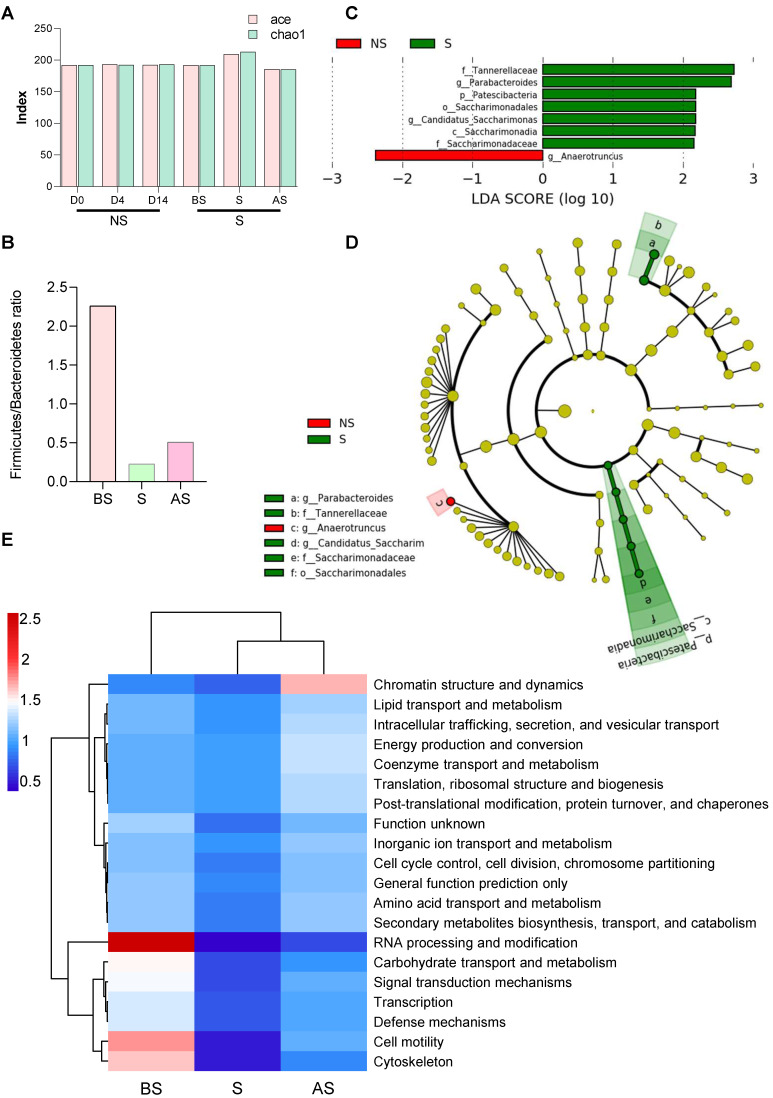
** Altered gut microbiome composition and metabolome in stress (A)** Alpha-diversity (ace and chao1) between the NS and S groups at three stages (D0, D4 and D14). **(B)** The ratio of Firmicutes and Bacteroidetes in NS and S groups at three stages. **(C)** Linear discriminant analysis effect size (LDA) between the NS and S groups at D4 stage. Red, bacterial taxa statistically overrepresented in NS samples; green, bacterial taxa overrepresented in S samples. The length of the bar represents log10 transformed LDA score. The absolute values of the effect size provide an interpretation of the scale of the difference between two groups for a certain taxon**. (D)** Taxonomic cladogram obtained from LEfSe analysis showing differentially abundant bacteria taxa between NS and S groups at D4 stage. Green represents increased abundance in the S group; red is increased abundance in the NS group. **(E)** COGs of PICRUSt analysis between NS and S groups at three stages. In this figure and below, BS, S, and AS were used to indicate the three stages when the S group compared with the NS stages (D0, D4, and D14). n ≥ 6 mice in each group.

16S rRNA gene sequencing was used to investigate the restraint-stress-induced changes in the microbiome that could lead to increased tumor development in mice. Ace and chao1 analysis of the fecal microbiome of non-stressed (NS) vs. stressed (S) mice showed that the stressed group had higher microbial alpha-diversity, even though the difference was not significant **(Figure 2A).** Principal component analysis (PCA) demonstrated that the fecal microbiome in NS mice clustered separately from S mice **(Figure S2A)**, suggesting both qualitative and quantitative differences in the two groups' gut microbiomes. Increased bacterial loads at the phylum, class, order level were observed in stress stages in stressed mice **(Figure S2B-G),** implying that stress regulated the gut microbiota response and altered the gut microbiome. Stress decreased the Firmicutes/Bacteroidetes (F/B) ratio **(Figure 2B)** in the S and AS stages. A Circos diagram shows the composition and abundance of the gut microbiome were different among the before stress, stress, and after stress stages (stress vs. non-stress) **(Figure S2H).** Compared with the even distribution of each OTU in the before stress group, OTU61 (*o_Rhodospirillales*), OTU59 (*Clostridiales*), and OTU62 (*o_Clostridiales*) were of highest abundance in the stress and after stress groups. The taxonomy of the differently distributed OTUs is listed in **Table S1**, while the microbial composition and disease link in stress is shown in **Table S2.** Three major taxonomic units, Clostridiales, Rhodospirillales, and Gastranaerophilales, are closely related to cancer.

A linear discriminant analysis (LDA) effect size (LEfSe) was applied to compare the microbiomes, demonstrating fine-scale differences in the bacterial taxon abundances between the non-stress and stress groups. We found seven differentially abundant clades (α = 0.05), including *g_Parabacteroides* in the stress mice and one differentially abundant clade in non-stress mice **(Figure 2C).** The cladogram **(Figure 2D)** summarizes the LEfSe associations by representing the taxonomic relationships and abundances between the two groups. Five taxa (a, b, d, e, f), in particular, significantly represent two certain phylogenies in the stress group. Stress mice exhibited a high abundance of *Lachnospiraceae* UCG-001, *Mucispirillum*, and *Rikenellaceae* RC9 gut group. In contrast, the non-stress group showed high levels of *Anaeroplasma* and *Ruminococcaceae* UCG-014 **(Figure S2I).**

After sequencing, we predicted the functional genes in the fecal samples using Phylogenetic Investigation of Communities by Reconstruction of Unobserved States (PICRUSt) analysis. The differences in the clusters of orthologous genes and pathways between the microbial communities were analyzed using the Clusters of Orthologous Genes (COG) database and Kyoto Encyclopedia of Genes and Genomes (KEGG) pathway analysis. After normalization, protein function classification demonstrated that stress stage samples had a lower abundance of all 24 COG gene families compared to the before stress microbial communities, but the abundance was restored in the after-stress stage. For example, COGs involved in chromatin structure and dynamics were more highly abundant in the regression period (after stress) **(Figure 2E).** Three COGs, (RNA processing and modification, cell motility, and the cytoskeleton), gradually decreased during stress from before stress to after stress.

Stressed microbiota were involved in diverse pathways at all three KEGG orthology levels (levels 1, 2, and 3). As shown in **Figure S2J**, metabolism was significantly enriched during stress compared to before stress and after stress stages in the level 1 category. A heatmap obtained from the level 2 analysis **(Figure S2K)** shows that pathways involved in carbohydrate metabolism, environmental adaption, cell motility, membrane transport, and transcription were highly enriched in samples taken before stress, while pathways classified to the digestive system and excretory system were more enriched in stress and after stress stages, and pathways involved the endocrine system, cancers, immune system, cell growth and death, were highly enriched after stress. Level 3 category results indicated that most annotated pathways were evenly distributed among all samples, with some outliers indicated **(Figure S2L).** Ether lipid metabolism was significantly enriched before stress, while a significantly greater proportion of genes involved in Parkinson's disease; various types of N-glycan biosynthesis and apoptosis was observed in after stress. Collectively, these results suggest that stress not only altered the composition of the gut microbiome but also changed the intestinal microbiome response which may lead to enhanced tumor growth.

### Stress alters fecal and serum metabolome

**Figure 3:**
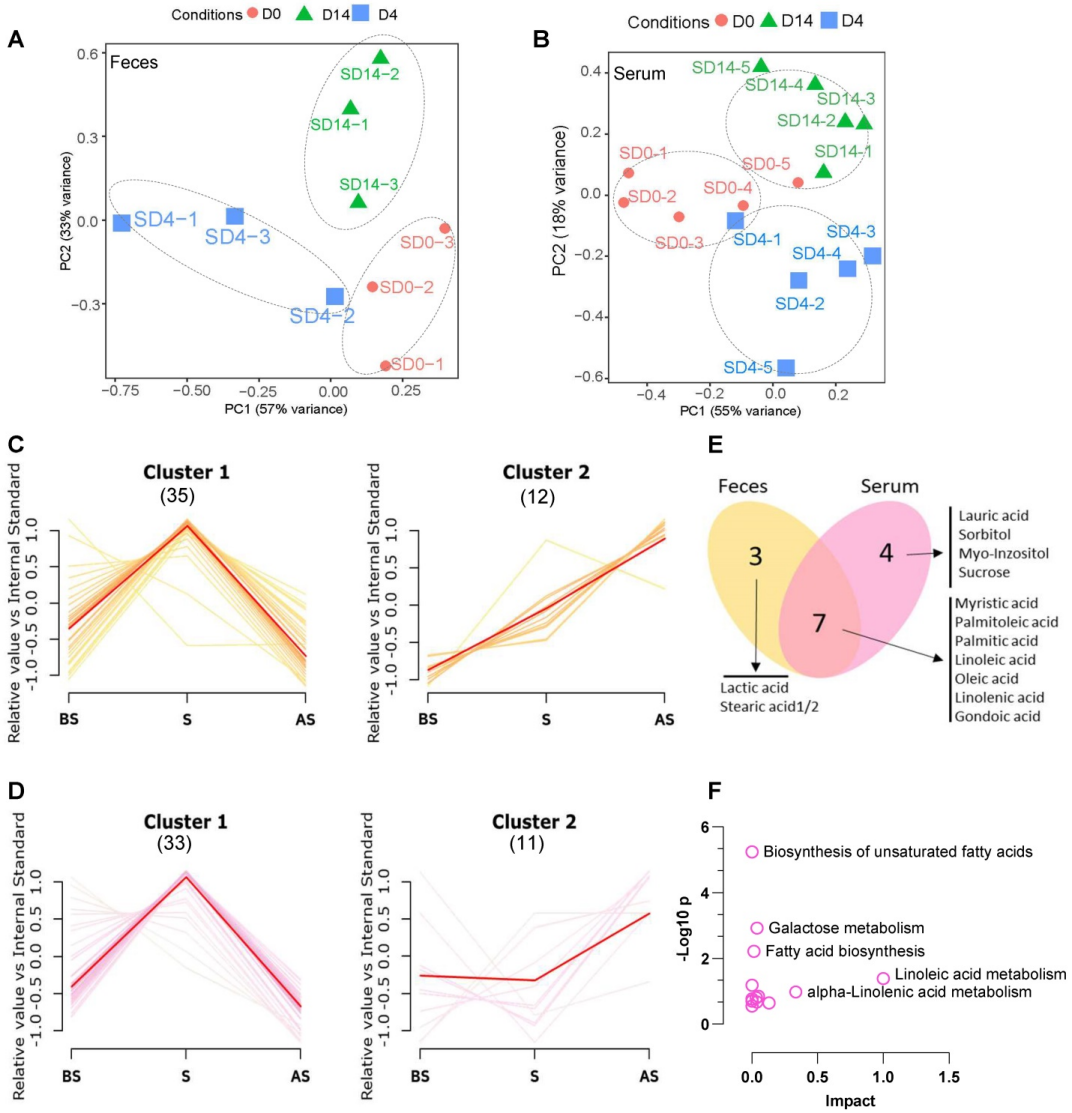
** Altered metabolome in stress (A, B)** PCA analysis of fecal and serum samples at three stages between NS and S samples. **(C)** Cluster analysis of fecal metabolites of S vs. NS samples at BS, S and AS stages; p < 0.01 Numbers in Parenthesis stand for the numbers of metabolites in the given cluster. **(D)** Cluster analysis of serum metabolites of S vs. NS samples at BS, S and AS stages; p < 0.01 **(E)** Venn diagram showing the overlap between Cluster 2 metabolites from fecal and serum samples. **(F)** KEGG analysis of the overlapping metabolites of Cluster 2 from fecal and serum samples according to MetaboAnalyst 5.0 30. The pathway whose *p*-value was below 0.05 was chosen as a potential target pathway. n ≥ 6 mice in each group.

Next, we wished to understand the metabolite status in feces and serum after stress and to study the relationship between changes in the composition of the microbiome and metabolism. First, untargeted profiling GC-MS analysis of the metabolites from fecal and serum samples of the stress group was performed. A total of 45 and 44 different metabolites were identified from the fecal and serum samples, respectively **(Table S3 and Table S4).** PCA analysis demonstrates the obvious clustering and separation of the before stress, stress, and after stress stages according to the fecal and serum samples **(Figure 3A-B)**.

The different fecal and serum metabolite levels in before stress, stress, and after stress, between the NS and S groups were compared using metabolite cluster analysis 29. In the fecal samples **(Figure 3C)**, the majority (35/45, 77.8%) of metabolites (Feces Cluster 1) increased during stress but reduced after stress, demonstrating a normal stress response. Approximately one-fourth (12/45, 26.6%) of the metabolites exhibited an adaptation response to stress (Feces Cluster 2), increasing from before stress to stress and remaining elevated after stress. In particular, some monounsaturated fatty acids, e.g., palmitoleic acid, linolenic acid, lactic acid, were up regulated in the stress and after stress fecal samples. Similarly, in the serum samples, the majority of metabolites showed the Cluster 1 pattern (33/44, 75.0%) **(Figure 3D)**, while 11 (25.0%) metabolites followed the Cluster 2 pattern. Seven metabolites were common to the Cluster 2 patterns in both the serum and feces samples **(Figure 3E),** which suggested that these altered metabolites exhibited a conserved pattern between feces and serum samples during stress. KEGG pathway analysis revealed that these metabolites are enriched in biosynthesis of unsaturated fatty acids, galactose metabolism, fatty acid biosynthesis, and linoleic acid metabolism **(Figure 3F).**

### The gut microbiome and metabolites show a positive correlation during stress

**Figure 4:**
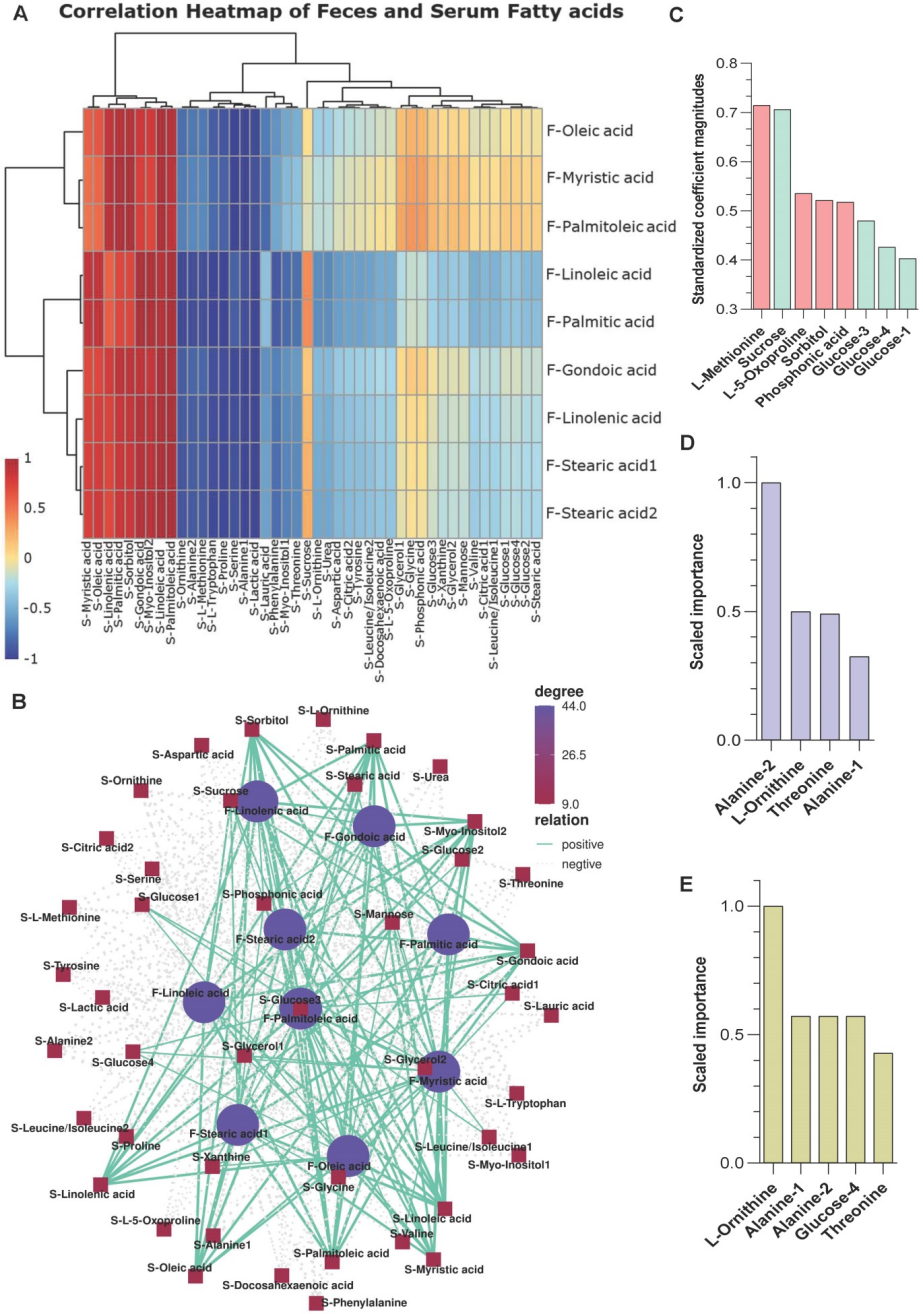
** Correlation analysis between the microbiome and metabolites (A-B)** Correlation analysis between fecal fatty acids and serum metabolites. There was a high degree of statistically significant concordance between fecal fatty acids and serum fatty acids, with p < 0.01.** (C-E)** Variable importance of metabolites from S serum samples using three machine learning approaches. **(C)** Coefficient magnitudes generated by generalized linear modeling (GLM) with 3-fold cross-validation; training AUC = 1 and testing AUC = 0.96. **(D)** Scaled importance generated by gradient boosting machine (GBM) with 3-fold cross-validation; training AUC = 1 and testing AUC = 1. **(E)** Scaled importance generated by distributed random forests (DRF) with 3-fold cross-validation; training AUC = 1 and testing AUC = 0.96.

A correlation matrix was generated to explore the functional correlation between the gut microbiome and fecal metabolite changes**.** Clear correlations were identified between the perturbed gut microbiome and altered metabolite profiles (p < 0.01). As shown in **Figure S3A**, the relative abundance of Cluster 2 fatty acids positively correlated with *g_Candidatus Saccharimonas* and *g_Parabacteroides*, (p < 0.05), while it negatively correlated with *g_Ruminiclostridium* 6, *g_Anaeroplasma* (p < 0.05)** (Table S5)**. The high correlation indicates that these specific bacterial taxa may enhance fatty acid metabolism pathway during stress. Further, the metabolites in feces and serum are well conserved in terms of function and expression **(Figure S3B).** In particular, the fatty acid metabolites (Cluster 2 pattern) in feces were positively correlated with fatty acid metabolites in serum samples from stress mice (p < 0.01) **(Figure 4A-B and Table S6)**.

Three machine learning methods, namely generalized linear modeling (GLM), gradient boosting machine (GBM), and distributed random forests (DRF), were used to predict potential metabolite markers in serum samples from S and NS mice. All three methods reasonably predicted that L-methionine, sucrose, alanine 1/2, L-ornithine, threonine, and glucose are candidate biomarkers for the stress response (training area under the curve [AUC] = 1 and testing AUC ≥ 0.96) **(Figure 4C-E).** Therefore, stress exposure induced a significant taxonomic perturbation in the gut microbiome *via* the brain-gut axis, which substantially altered the metabolomic profile of the gut/feces metabolites and, thus, the serum metabolites.

### Stress affects the epigenetic signature in mammary tumors

#### Genome-wide mCpG site analysis

As the gut microbiome is distant from the site of breast tumors, we propose that a potential systemic influx of microbiome-regulated metabolites from the feces to the blood may affect the epigenetic signature in tumors during stress by chromatin dynamics, whose activity is directly dependent on metabolites 26, 31. Bisulfite sequencing enabled the acquisition of the genome-wide DNA methylation landscape at the single-base resolution in tumors from NS and S mice. There were three contexts of C bases on the genome, CG, chlorhexidine gluconate (CHG), and CHH, where H represents any base of A, T, C. We first identified the percentage of methylated cytosines in each context. The average ratios of methylated mCG to total CG were 68.2% and 65.7% in the NS and S samples, respectively **(Figure S4A)**. Dramatically low methylation statuses were found for the CHG and CHH sites. Among these detected mC sites, mCHH represented the highest proportion (~50%), mCpG (CG sites) made up a moderate proportion (~35%) and mCHG accounted for the smallest proportion (`15%) of methylation sites **(Figure S4B)**. We observed significant differences (p < 0.05) between non-stressed and stressed samples for all three mC contexts.

**Figure 5:**
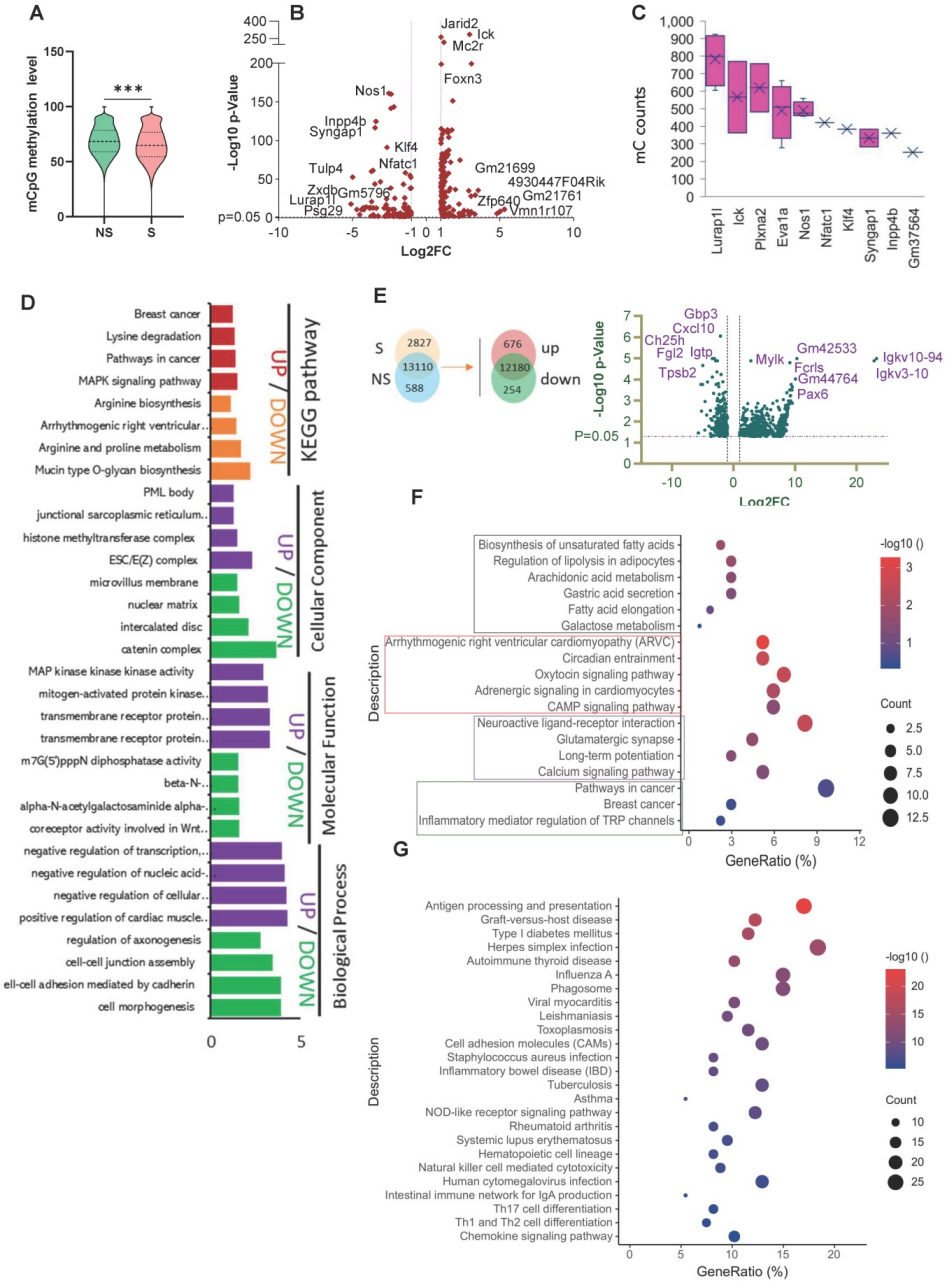
** Stress affects the epigenetic signature and gene expression changes in mice breast tumors. (A)** Distribution of the methylation rate of mCpG sites on the whole genome. **(B)** Summary of DMR-related gene (DMG) results. Volcano plot representation of DMGs in the S and NS tumor samples. The X-axis shows Log2fold-changes in methylation level, and the y-axis is the -Log 10 p-value of a DMG. **(C)** The top 10 DMGs for mC count. **(D)** GO and KEGG enrichment analysis of DMGs in the S tumor samples**.** Biological processes (BP), cellular components (CC), and molecular function (MF) **(E)** Venn diagram showing the co-expression and up/down-regulation of genes in NS and S tumor samples (top). Volcano plot of RNA-seq transcriptome data displaying significantly differentially expressed genes (DEGs) from tumor samples with or without stress (bottom). Significant DEGs (FDR-corrected P ≤ 0.05) are highlighted in blue, with the grey lines representing the boundary for identification of up- or down-regulated genes (Log2 FC>1). Selected top high-expression genes related to stress response are indicated. **(F, G)** KEGG pathway analysis of up- and down-regulated DEGs significantly enriched in functional categories (P ≤ 0.05). The gene ratio is the ratio of the DEG number and the number of all genes in a certain enrichment pathway. The dot size denotes the number of DEGs, while colors correspond to the adjusted p-value range**.** All transcriptome experiments were performed in biological triplicate. One-way analyses of variance (ANOVA) were used to determine the inter-group differences between two groups for one or two variables (***p < 0.001).

Next, we calculated the methylation density and average methylation level in each gene segment or transcript in the promoter, 5′UTR, exon, intron, and 3′UTR areas. A higher density of mCHG and mCHH methylation was found in the five genomic areas in the S group, particularly in exons **(Figure S4C)**. In comparison, a higher mCG methylation density was observed in almost all five genomic regions in the NS group. Compared with mCHG and mCHH, mCG showed the highest average methylation level in all five regions in both groups **(Figure S4D)**. A significantly higher average CpG site methylation level was observed in the NS group compared to the S group (*p < 0.0001*) **(Figure 5A)**.

#### Differentially methylated regions (DMR) and Gene (DMG) analysis

A total of 2,225 DMRs were identified under the CG context that included 254 significant DMGs (p < 0.01*,* |Log_2_FC| ≥ 1) **(Table S7)** in NS vs S tumors. Among these DMGs, 106 had down-regulated methylation levels, and 148 had up-regulated methylation levels in the S tumors. The volcano plot in **Figure 5B** provides a general overview of the DMGs between the two groups of samples. We next investigated the number of mC sites in each given DMG. *Lurap1l, Ick, Plxna2, Eva1a, Nos1, Nfatc1, Klf4, Syngap1, Inpp4b,* and *Gm37564* had the highest numbers of mC sites **(Figure 5C)**. The distribution of DMRs in each genomic feature is presented in the Sankey diagrams **(Figure S4E),** which show the genomic region and chromosome distributions of the DMGs.

We divided these DMGs genes into those that were up or downregulated in the S group and performed separate GO category and KEGG pathway analyses. The genes of the up and down-regulated groups were enriched to very different functional categories. MAPK signaling pathway, pathways in cancer, and breast cancer were associated with upregulated DMGs **(Figure 5D)**, while arginine and proline metabolism and arginine biosynthesis were associated with down-regulated DMGs in S tumors. In terms of GO enrichment analysis, histone methyltransferase complex was key in the upregulated group, and another methylation-related GO item, m7G (5') pppN diphosphatase activity, was associated with a down-regulated DMG set (NUDT10) from S tumors. Our data suggest that DNA methylation changes are involved in regulating metabolism, histone methyltransferase, and other critical breast cancer signaling pathways.

### Stress induces genome-wide gene expression changes in tumors

We performed RNA-seq analysis on NS and S breast tumor samples to identify the functional consequences underlying the negatively and positively altered DMGs under stress. We obtained 930 DEGs, including 676 significantly up-regulated genes and 254 significantly down-regulated genes, in the S vs. NS tumors. A volcano plot is used to visualize the results, in which the top 13 significantly changed genes were plotted **(Figure 5E).** A heatmap is also used to compare the levels of DEGs between non-stress and stress groups **(Figure S4F).**

To further analyze the functional variation in the DEGs, we used GO and KEGG analyses. The most significantly enriched GO terms in the BP, CC, and MF categories in the up-regulated DEGs were regulation of membrane potential, cell-cell junction, and inorganic cation transmembrane transporter activity. Downregulated DEGs were significantly enriched in pathways related to inflammation **(Figure S4G-H)**. KEGG pathway enrichment analysis showed that the up-regulated DEGs were mainly involved in fatty acid biosynthesis and metabolism and cancer **(Figure 5F),** while the down-regulated DEGs were again enriched in pathways and diseases related to inflammation **(Figure 5G)**. In addition, gene set enrichment analysis (GSEA) revealed that the relatively high-ranking pentose and glucuronate interconversions, fat digestion and absorption, and steroid hormone biosynthesis gene sets were positively associated with stress; while three gene sets, cytokine-cytokine receptor interactions, glycosaminoglycan biosynthesis chondroitin sulfate dermatan sulfate, and primary immunodeficiency, were negatively associated with stress **(Figure S5)**.

### DNA methylome and transcriptome analyses reveal the correlated regulation of hub genes in stress-promoted tumorigenesis

**Figure 6:**
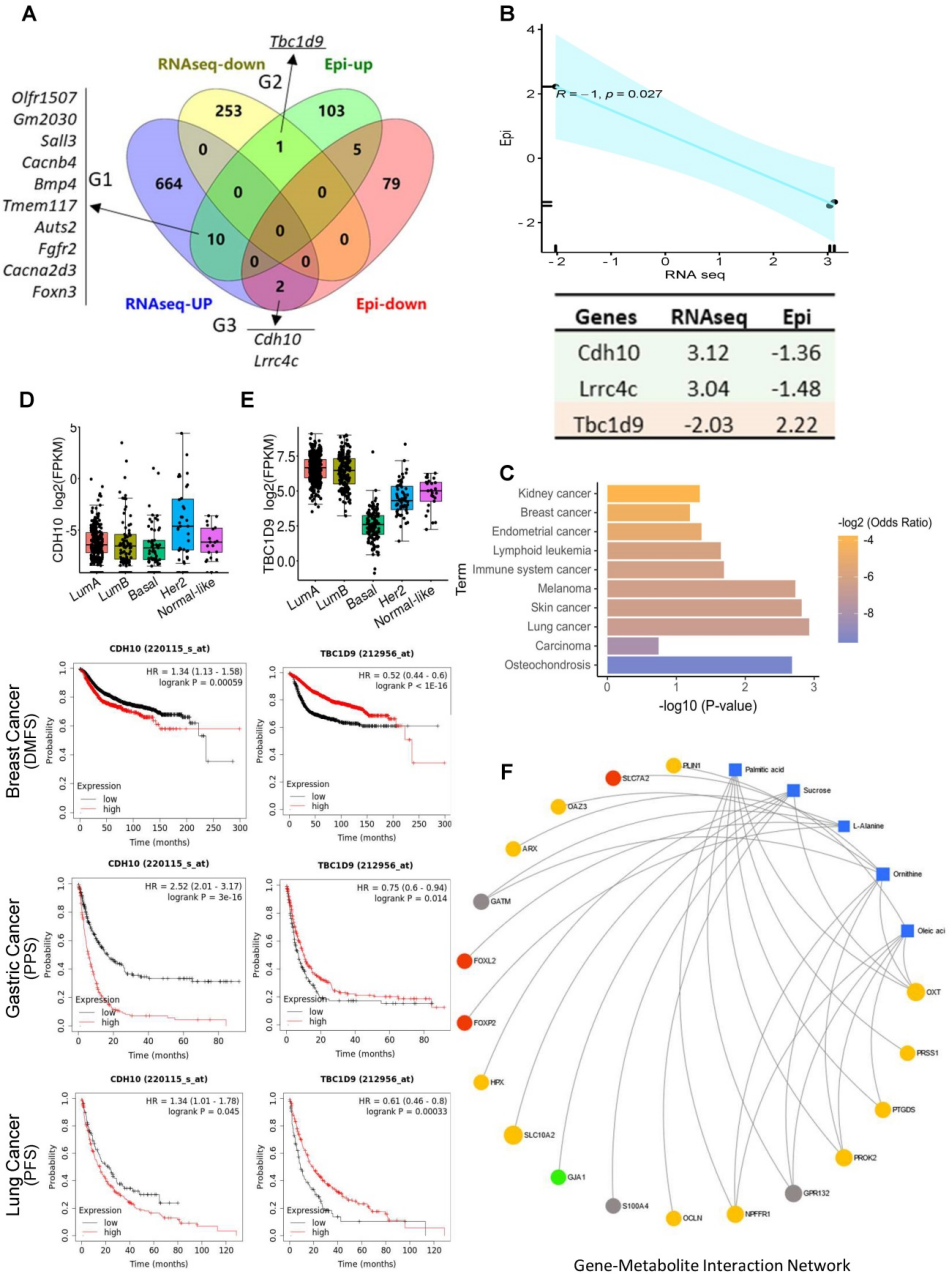
** Integrated gene-metabolite network analysis. (A)** Venn diagram comparing DMGs and DEGs. A total of 13 genes were identified from up- and down-regulated DMGs and DEGs. There were three groups identified: G1 (n = 10) genes were hypermethylated in the promoter with high gene expression level in tumor; G2 (n = 1) genes were hypermethylated in the promoter with low expression in tumor; G3 (n = 2) genes were hypomethylated in the promoter with high expression in S tumors. **(B)** Pearson correlation analysis of G2 and G3 genes; R = -1, p = 0.027. Epi and RNAseq stand for the fold change of given genes in methylation levels and RNA expression in S samples. **(C)** KEGG pathway analysis of G2 and G3 genes; p > 0.05. **(D-E).** Kaplan-Meier survival curves show the correlation between marker gene expression and distant metastasis free survival (DMFS), post progression survival (PPS), and progression free survival (PFS) in breast, lung, and gastric cancer. Gene expression in different breast cancer types on the top. FPKM: fragments per kilobase of transcript per million fragments mapped. **(F)** Metabolite-gene interaction network analysis related to fatty acid metabolism and cancer pathways. The correlation network is composed of five metabolite compounds combined with 18 genes. Metabolites are represented by blue diamonds and genes by circles. Yellow: up-regulated DEGs. Grey: down-regulated DEGs. Red: hypermethylated DMGs. Green: hypomethylated DMGs. Metabolites with KEGG annotations from the merged data set were mapped to KEGG reference pathways, and interaction networks were generated in MetaboAnalyst5.0 (p < 0.005).

DNA methylation at gene regulatory regions is usually considered to influence transcript expression levels 32. We merged the DMGs and DEGs and identified 13 genes that showed significantly differential methylation and gene expression **(Figure 6A)**. According to the methylation and gene expression patterns, the overlapping genes between the DMGs and DEGs were classified into three distinct groups (denoted G1, G2, and G3). Genes in G2 (*Tbc1d9*) and G3 (*Cdh10, Lrrc4c*) followed the canonical model (a negative correlation between promotor methylation and gene expression) (R = -1, p = 0.027) **(Figure 6B)** 33, with *Tbc1d9* downregulated and *Cdh10* upregulated in stress tumors, implying that methylation may play a direct role in the regulation of transcriptomic-level phenotypes in stress-induced tumor development. In contrast, genes in G1 showed a non-canonical and weak correlation pattern (R = 0.44, p = 0.2) **(Figure S6A).** KEGG pathway analysis demonstrated that G2 and G3 genes are enriched in cancer **(Figure 6C).** G1 genes were enriched in the MAPK signaling pathway and the oxytocin signaling pathway **(Figure S6B).** We validated the expression of *Cdh10* and *Tbc1d9* in breast cancer subsets using the TCGA dataset (http://tcgaportal.org/TCGA/Breast_TCGA_BRCA). *Cdh10* expression was not different throughout the subsets, while a lower expression of *Tbc1d9* (p < 0.001) was observed in the ER-negative basal-type and HER2+ breast cancers (p < 0.05) **(Figure 6D-E).** Using Kaplan-Meier survival curves (https://kmplot.com/analysis/) for distant metastasis free survival (DMFS) for breast cancer, higher expression of *Cdh10* was associated with worse DMFS. In contrast, lower expression levels of *Tbc1d9* were associated with prolonged DMFS (p < 1e^-16^) **(Figure 6D-E and Figure S7)**. Similarly, higher expression of *Cdh10* was associated with significantly worse post-progression survival (PPS, p < 1e^-16^) in gastric cancer datasets and progression free survival (PFS) in lung cancer patients, while lower expression levels of Tbc*1d9* in gastric cancer and lung cancer patients resulted in better PPS and PFS, respectively **(Figure 6D-E and Figure S7)**. This clinical data demonstrates a positive correlation to our mouse stress studies, linking stress to DNA methylated genes *Cdh10* and *Tbc1d9,* related to cancer prognosis and survival.

### Integrated analysis of multi-omics data reveals the regulation network in stress-enhanced tumorigenesis

We next performed intergraded network analysis combined with metabolites with the DMGs/DEGs data. The metabolite-gene-disease interaction network showed that the list of metabolites identified, such as ornithine, linoleic acid, l-alanine, are involved in Alzheimer disease and lung cancer, amongst others. 25 significantly differently expressed hub genes in the network indicated that this new emergent network is composed of metabolites, genes, and related disease pathways **(Figure S8).** Neuronal diseases and cancer pathways share some of the same metabolites and genes, showing a strong brain-cancer link. A more detailed gene-metabolite network analysis revealed the relationships between fatty acid metabolism and cancer pathways. Five metabolites and 18 hub genes were identified, and the correlation networks were constructed **(Figure 6F).** The up-regulated DEGs mainly contributed to the activation of palmitic acid and oleic acid metabolism or synthesis pathways. Two forked family genes (*Foxl2 and Foxp2*) contributed to L-alanine synthesis pathways, suggesting that the DMGs/DEGs are involved in stress-induced fatty acid biosynthesis during tumorigenesis.

These hub genes are enriched in the inflammatory response, neuroactive ligand-receptor interactions, and glycine, serine, and threonine metabolism pathways **(**KEGG, **Figure S9A).** Seventeen hub genes were significantly enriched in GO classes for terms such as positive regulation of metabolic processes, glutathione transmembrane transporter activity **(**GO, **Figure S9B).** Interestingly, all the metabolites mentioned above were also directly connected to brain disorders, such as Alzheimer's disease, as well as hyperglycinemia. These results further confirm the concept that the brain-gut axis is a leading player in stress-enhanced tumorigenesis. In addition to the fatty acid metabolic changes in tumorigenesis after stress, the downregulated DEGs were significantly enriched in the inflammation pathways. Based on our current results and our previous findings that ANXA1 is involved in breast cancer growth, we hypothesized that ANXA1 may also play a role in stress-induced tumor development.

### ANXA1 deficiency reshapes the murine gut microbiome

**Figure 7:**
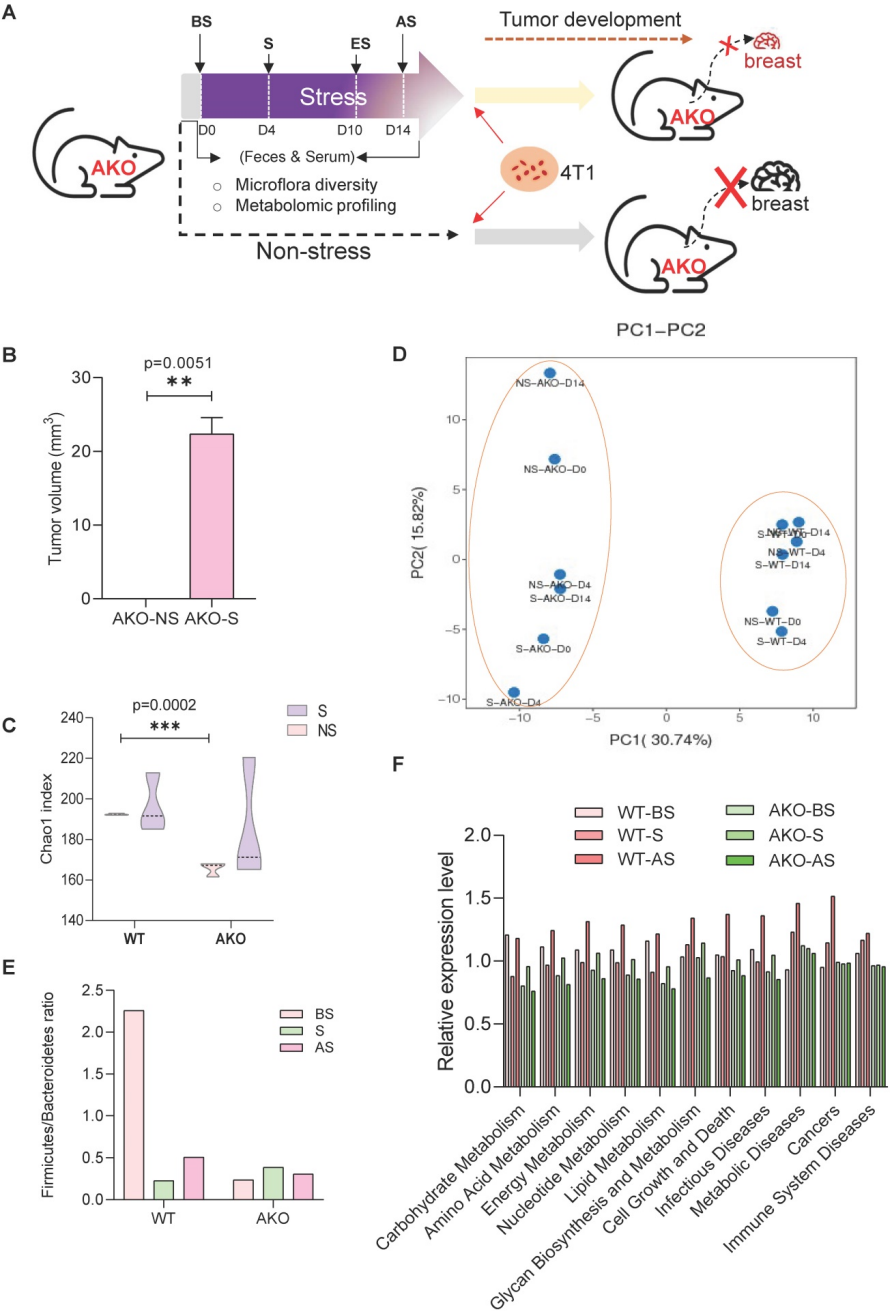
** ANXA1 deficiency alters gut microbiome structure under stress (A)** Schematic of experimental design and sample collection protocol for ANXA1-knockout mice. n ≥ 6 mice in each group. **(B)** Orthotopic tumor weight 36 days after injection of 4T1 cells. Results were obtained in three independent experiments. The data are shown as the mean ± SD; **p < 0.01. **(C-D)** Alpha-diversity and PCA analysis of bacteria varied across ANXA1^+/+^ and ANXA1^-/-^ fecal samples; ***p < 0.001 **(E)** Ratio of Firmicutes and Bacteroidetes in ANXA1+/+ and ANXA1^-/-^ fecal samples. **(F)** KEGG analysis of ANXA1^+/+^ and ANXA1^-/-^ feces samples under stress conditions. (WT, ANXA1^+/+^; AKO, ANXA1^-/-^).

ANXA1 knockout (ANXA1^-/-^) mice were subjected to restraint stress conditions, and fecal and serum samples were collected for microbiome and metabolism analyses **(Figure 7A).** ANXA1 deficiency significantly reduced tumor growth to non-existent levels in the NS mice and S mice (p = 0.0051). Even with stress, the ANXA1^-/-^ mice exhibited significantly smaller tumor volumes **(Figure 7B)** than wild-type (ANXA1^+/+^) mice **(Figure 1C).** We performed identical analyses in ANXA1^-/-^ feces as described in **Figure 2A**. ANXA1^-/-^ exhibited significantly lower α diversity under NS conditions, suggesting that the gut microbiome in ANXA1^-/-^ were more sensitive to stress **(Figure 7C)**. β-diversity composition of the fecal microbiome was significantly affected by ANXA1 deficiency (p < 0.001) **(Figure 7D).** The changes in the fecal microbiota after stress in ANXA1^-/-^ were explored at different taxon levels (**Figure S10A-F**). At the phylum level, the F/B ratio in BS ANXA1^-/-^ feces samples was significantly lower than ANXA1^+/+^ BS feces and did not change upon stress **(Figure 7E).** The enrichment in gut microbiota functions of metabolism, cell growth and death; metabolic diseases; cancers; and immune system diseases in response to stress in the ANXA1^+/+^ mice was abolished in the ANXA1^-/-^ mice **(Figure 7F).**

A cladogram representative of the structure of the fecal microbiota and their predominant bacteria in NS and S ANXA1^-/-^ feces is shown in **Figure S11A-B**, in which the greatest differences in taxa between the two communities are displayed using LEfSe. In addition, tracking individual OTU abundances revealed different dynamics within the dominant microbiota in ANXA1^-/-^ mice to those of ANXA1^+/+^ samples **(Figure S11C, Table S8).** Microbiota species in the S ANXA1^-/-^ feces mainly belonged to *Saccharimonadia, Lachnospiraceae,* and *Bacteroidales*. These microbes, different from those in the ANXA1^+/+^ sample, are lowered in many diseases, including colorectal cancer, obesity and type 2 diabetes **(Table S9).** Collectively, these changes in the fecal microbiota revealed that deleting ANXA1 expression attenuated the stress-induced gut microbiome dysbiosis.

### ANXA1 deficiency influences metabolism and methylation

**Figure 8:**
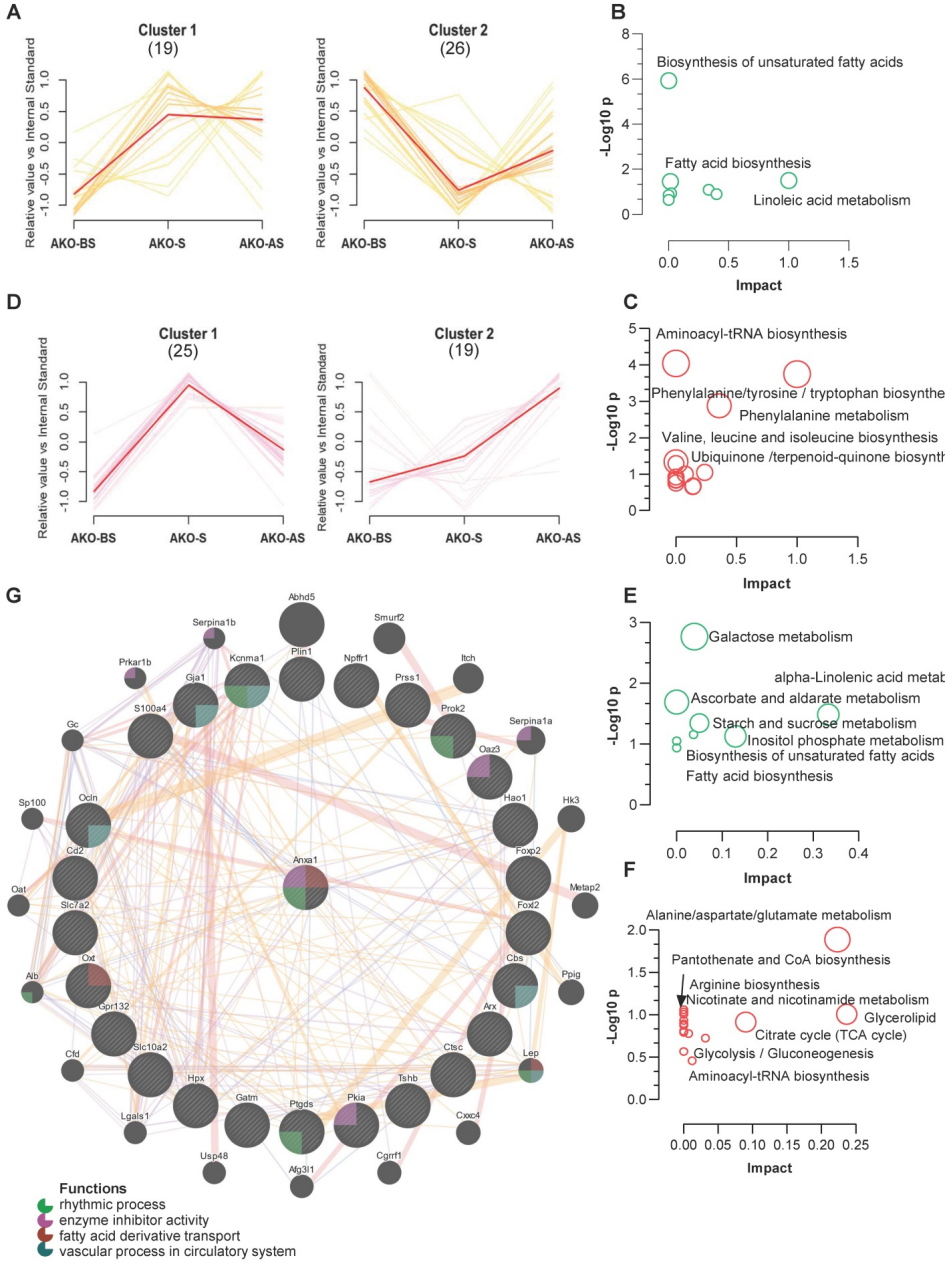
** ANXA1 deficiency changes feces and serum metabolites (A, D)** Metabolite clustering results (time-series line) based on metabolite expression levels. The horizontal axis denotes stress procedure timepoints (BS, S and AS) at which fecal **(A)** and serum** (D)** samples of ANXA1^+/+^ and ANXA1^-/-^ mice were collected. The vertical axis denotes the levels of the metabolites relative to the internal standard. Numbers in brackets indicate the number of metabolites in each cluster. **(B)** KEGG analysis of the 11 suppressed metabolites transferred from ANXA1^+/+^ -C2 to ANXA1^-/-^ -C2 in fecal samples. **(C)** KEGG analysis of the 15 increased metabolites transferred from ANXA1^+/+^ -C1 to ANXA1^-/-^ -C2 in fecal samples. **(E)** KEGG analysis of the 4 suppressed metabolites transferred from ANXA1^+/+^ -C2 to ANXA1^-/-^ -C1 in serum samples. **(F)** KEGG analysis of the 12 increased metabolites transferred from ANXA1^+/+^ -C1 to ANXA1^-/-^ -C2 in fecal samples. **(G)** Interaction network constructed with GeneMania for highly altered hub genes and *Anxa1*. The color of the lines connecting the genes depicts the type of interaction (purple for co-expression, yellow for predicted, blue for co-localization, and pink for physical interaction). The colors in the circles indicate the functions (see key).

Using a clustering algorithm to classify the levels of metabolites in all stress stages (as described in **Figure 3C-D**), two major distinct clusters (C1 and C2) of patterns representing differentially regulated metabolites from fecal and serum samples of the ANXA1^-/-^ mice. Combined with the clustering data shown in **Figure 3C-D,** 11 metabolites from ANXA1^+/+^ -C2 (enhanced at S and AS, **Figure 3C**) were suppressed to ANXA1^-/-^ -C2 (reduced at S and AS), while 15 metabolites from ANXA1^+/+^ -C1 (enhanced at S only, **Figure 3C**) were increased to ANXA1^-/-^ -C2 in the ANXA1^-/-^ fecal samples **(Figure 8A).** The suppressed metabolites in ANXA1^-/-^ were enriched in the biosynthesis of unsaturated fatty acids, linoleic acid metabolism, and fatty acid biosynthesis, suggesting that ANXA1 alters fecal microbiome metabolism by enhancing fatty acid metabolism during stress **(Figure 8B).** Moreover, the increased metabolites in ANXA1^-/-^ included aminoacyl-tRNA biosynthesis, phenylalanine, tyrosine and tryptophan biosynthesis, and phenylalanine metabolism, suggesting that these pathways may be involved in tumor suppression in ANXA1^-/-^ **(Figure 8C).** Similarly, we observed clear differences in the clusters between the ANXA1^+/+^ and ANXA1^-/-^ serum samples **(Figure 8D).** Four metabolites were suppressed in ANXA1^+/+^ -C2 to ANXA1^-/-^ -C1 in serum samples, and 12 metabolites from ANXA1^+/+^ -C1 to ANXA1^-/-^ -C2 were increased in ANXA1^-/-^ S serum. Similarly, the suppressed serum metabolites were enriched in fatty acid biosynthesis, while the increased serum metabolites were enriched in alanine, aspartate, and glutamate metabolism pathways **(Figure 8E-F)**. When these differentially regulated metabolites in the feces and serum were compared, three pathways: biosynthesis of unsaturated fatty acids, fatty acid biosynthesis, and alpha-linolenic acid metabolism, were commonly suppressed. Three other pathways, aminoacyl tRNA biosynthesis, glycerolipid metabolism, and glycolysis/gluconeogenesis, were commonly increased**).** Moreover, correlation analysis further demonstrated that all the metabolites in the fecal samples were strongly connected to those in the serum samples from the ANXA1^-/-^ mice **(Figure S12 A).**

Machine learning analysis showed consistent results among three approaches that L-5-Oxoproline, L-Methionine, and Mannose represent the high AUC. The prediction analysis demonstrated the S ANXA1^-/-^ mice would have a different metabolite composition compared to the ANXA1^+/+^ **(Figure 4C-E, Figure S12B-D).** The machine learning prediction results further demonstrated that ANXA1 deficiency changes the levels of the potential metabolite markers in the S serum samples. Accordingly, ANXA1 deficiency may alert the gut microbiome and regulate fatty acid metabolism in the feces and serum, leading to the remote inhibition of tumorigenesis.

To further investigate the role of ANXA1 in fatty acid synthesis, the expression of fatty acid synthase (Fasn) and the upstream protein-ATP citrate lyase (Acly) were assessed. ANXA1 deficiency suppressed the protein expression of Fasn and Acly, but not mRNA **(Figure S13A, B).** Furthermore, the effect of ANXA1 deficiency on DNA methylation was assessed. DNA methyltransferase (cytosine-5) 1(Dnmt1) mRNA and protein expression were not significantly different between WT and ANXA1 -/- 4T1 cells **(Figure S14A)**. However, mRNA and protein expression of lysine (K)-specific demethylase 1A (Lsd1) was significantly lower in 4T1 cells deficient in ANXA1 **(Figure S14B)**.

### Correlation of ANXA1 with hub genes to regulate tumor development

Finally, as no tumors developed in the ANXA1^-/-^ mice, we next determined if ANXA1 interacts with hub genes to enhance oncogenesis based on the RNA-seq and epigenetic analysis of the ANXA1^+/+^ tumor samples. The correlation network results showed that *Anxa1* is involved in vascular processes in the circulatory system, rhythmic processes, enzyme inhibitor activity, and fatty acid derivative transport *via* interactions with *Ocln, Cbs, Ptgds, Pkia, Prok2, Gja1, Kcnma1, Oaz3,* and* Oxt* **(Figure 8G).** In addition, two upregulated hub DEGs, *Plin1* and* Oxt*, were positively correlated with *Anxa1* expression in breast cancer patients **(Figure S15A-B)**. *Plin1* was found to function as a modulator of adipocyte lipid metabolism, and *Oxt* is involved in cognition, tolerance, adaptation, and stress responses 34, 35.

Therefore, we here propose a model that illustrates the entire process through which stress promotes tumorigenesis *via* modulation of the microbiome composition and regulation of metabolism and resulted in the alteration of epigenetic signatures in our mouse model of breast cancer*.* ANXA1 deficiency suppresses tumor growth by altering the gut microbiome, regulating serum metabolite levels, and interacting with hub genes. All these changes involved fatty acid metabolism, the inflammatory stress response, and changes to neuro-hormone levels **(Graphical Abstract).** We have defined this microbiome-mediated metabolite and epigenetic interactions that caused breast cancer cell proliferation and collective feedback loops as the SMMEO axis. Brain (stress)-gut-tumor crosstalk, especially in non-gastrointestinal cancers, remains a crucial area of discovery.

## Discussion

Humans are increasingly exposed to environmental factors that influence cancer development as the intensities of their lifestyles increase. Many studies have investigated the impact of stress in the promotion of cancer. Moreover, researchers have also tried to illustrate the impact of the gut microbiota on treatment responses to cancer 36, 37. To our knowledge, our study is the first to systematically evaluate the role of stress in breast tumorigenesis through a multi-omics approach. This study will assist our understanding in determining how relevant the gut microbiome, metabolism, and tumor epigenetic signatures are to human breast cancer and what interventional strategies could be employed to improve patient outcomes.

Our study has demonstrated that exposure to stress prior to the initiation of mammary cancer contributes to increased tumor growth, and that perturbations in the gut microbiome caused by stress can affect tumor growth at a distant site, which is in line with previous findings that suggest the gut microbiome has endocrine functions 18, 38, 39. A number of metabolites associated with particular gut bacterial species correlated with DMG expression changes. Therefore, stress signals can disrupt the gut microbiome composition *via* the brain-gut axis and change gut and blood metabolites. Subsequently, these blood metabolites can mediate epigenetic and gene expression changes in the breast tumor microenvironment **(Graphical Abstract).**

The F/B ratio is regarded to be of significant relevance in human gut microbiota composition 40, where a lower F/B ratio was observed in breast cancer survivors 41. Our results showed that stress decreased the F/B ratio, which is consistent with previous studies 42-44. Interestingly, the profile and abundance of the microbiome may return to the baseline after stress, or the microbiome may adapt to a new state. This latter pattern may continuously affect downstream pathways to promote disease and tumorigenesis. Hence, the composition of the gut microbiome exists in a dynamic equilibrium that becomes more complex after stress 38.

An early study showed that the abundance of Clostridiales is closely related to steroidal estrogen production, contributing to breast carcinogenesis and stimulating tumor growth 45. More detailed studies demonstrated that Clostridiales increases bile acid degradation in breast cancer patients 46. A secondary bile acid, lithocholic acid (LCA), reduced breast tumor cell proliferation, aggressiveness, and metastatic potential of primary tumors through mesenchymal-to-epithelial transition and enhancing antitumor immune response 47. Moreover, Faecalibacterium prausnitzii, a member of Clostridium cluster IV, suppressed the proliferation and invasion and promoted the apoptosis of breast cancer cells *via* inhibition of the secretion of IL-6 and the phosphorylation of JAK2/STAT3 in MCF-7 cells 48, 49. The collagenase enzyme of the bacterium Clostridium histolyticum (CCH) was shown to reduce cell proliferation and wound healing in breast cancer MDA-MB-231 cells 50. In addition, dietary fat affects Clostridiales level while smoking increases Rhodospirillales abundance and impacts the tumor microbiome in lung cancer 51. Accordingly, different lifestyles, including stress may contribute to the gut microbiome composition and may thus be linked to cancer development.

Besides gut microbiota, breast microbiome can differ between healthy and cancer patients and contributes to the pathology of cancer *via* regulation of lipid signatures 52. Accumulating evidence suggests that the microbial composition of breast tissue plays an important effect in cancer development 53-57. It has been proposed that the endogenous and exogenous microbiome from the breast contributes to the maintenance of normal function of breast tissue either by stimulating resident immune cells or regulating metabolism 58. Moreover, cholesterol (and lipid) metabolism has been implicated in breast cancer carcinogenesis 59-61. In addition, cholesterol is a risk factor for breast cancer, through its impact on membrane fluidity and signaling pathways 62. High plasma cholesterol levels can enhance the proliferation of tumor cells and tumor growth in mouse models 63. Cholesterol and other lipid metabolites may act as biomarkers for breast cancer development and provide a novel target for cancer therapy.

Short-chain fatty acids (SCFAs) are known to play crucial roles in immune regulation and inflammatory diseases 64-66. However, the underlying mechanisms of how long-chain fatty acids (LCFAs) influence brain physiology, gut microbiota, gut-brain communication, and tumor development have not been fully elucidated 67. Cancer cells have an exuberant ability to synthesize fatty acids to maintain and promote cell growth and cancer progression 68. In our study, stress significantly altered the levels of fatty acids in the feces and serum, which indicates that stress may promote breast tumor development *via* fatty acid metabolism and biosynthesis. A number of metabolites were directly shown to correlate with different microbiome taxonomic levels and DEGs/DMGs. Notably, the up-regulated DEGs mainly contribute, *via* the gut-metabolite-gene network, to the biosynthesis of LCFAs palmitic acid and oleic acid. Palmitic acid and oleic acid bridge the link between fatty acid biosynthesis, metabolism and cancer pathways, further supporting the notion that the brain-gut axis plays a vital role in tumorigenesis. Four hub genes, Npffr, Gpr132, Prok2, and Ptgds were commonly connected with palmitic acid and oleic acid, suggesting the functional conservation of these genes in fatty acid metabolism.

Bioactive metabolites such as short-chain fatty acids, amino acid metabolites, or secondary bile acids secreted from the gut microbiome play an essential role in regulating breast cancer development 69. The fatty acids highlighted in Figure S3A have previously been shown to be involved in breast cancer development. Epidemiological studies have shown that elevated serum concentrations of oleic acid together with low levels of stearic acid are associated with an increased breast cancer risk 70, 71. High levels of palmitoleic acid to palmitic acid concentrations are associated with an increased breast cancer risk and can enhance cancer development 72-74. The influence of stearic acid on the inhibition of tumor cells *in vitro* and tumor development *in vivo* has been reported 75. Stearate induces apoptosis preferentially in breast cancer cells this may be protein kinase C dependent 76. The protective association provided by stearic acid has been previously reported in premenopausal women in a meta-analysis of fatty acids in biological samples and BC risk 77. In a meta-analysis of 12 prospective studies, both linoleic acid intake and serum levels of linoleic acid were associated with decreased breast cancer risk. However, none of the associations was statistically significant 78. In animal studies, α-linolenic acids have been shown to suppress the growth and proliferation of cancer cells and promotes breast cancer cell death 79-81. α-linolenic acid reduces growth and inhibits the migration of both triple-negative and luminal breast cancer cells in high and low estrogen environments 82, 83. Our results have confirmed that fatty acids play an essential and complex role in breast cancer development.

Similar to SCFAs 84, blood-borne microbial metabolites, including lithocholic acid (LCA) 85, 86, deconjugated estrogens 87, and amino acid degradation products can remotely execute their physiological function. Anaerobic bacteria, such as Clostridiales, are responsible for bile acid transformation from gut origin to breast 88, 89. The synthesis of LCA from the microbiome was vastly reduced in the advanced stage of breast cancer, pointing towards an antineoplastic role of LCA. LCA inhibited epithelial-to-mesenchymal transition and metastasis formation in breast cancer cells 85, and a high concentration of LCA inhibits fatty acid biosynthesis which could promote cell death 86, 90. Interestingly, a large-scale metabolomics analysis demonstrated that a high level of microbiome-derived deoxycholate was found in breast tumors which was inversely associated with the cell proliferation in breast tumors, suggesting that this bile acid accumulation in the breast could be used for breast cancer survival prediction. 91. In terms of amino acid degradation, bacteria synthesize cadaverine from lysine using the enzymes LdcC and CadA^92^. Similar to LCA, the cadaverine expression in gut microbiome is decreased in the later stages of breast cancer^93^. Moreover, cadaverine inhibits cell proliferation, cell growth and invasion, and tumor infiltration to the surrounding tissues by changing metabolism or reducing the proportion of ALDH1+ cancer stem cells 93. Cadaverine exerts its function through the trace amine-associated receptor-1, 2, 3, 5, 8, 9 (TAAR1, 2, 3, 5, 8, 9), of which TAAR1 has tumor suppressive roles 94. These metabolites secreted from the microbiome are critical constituents of the tumor microenvironment involved in breast cancer development. The dysregulation of the same metabolic pathways in tumors and the breast tumor microbiome therefore suggest an interconnection between the tumor and the corresponding microbiome.

Previous reports have shown that microbiota can directly affect fatty acid metabolism levels. Multiple metagenomes analyses have demonstrated that g_Candidatus Saccharimonas can negatively regulate short-chain fatty acids metabolism 95. Candidatus_Saccharimonas were significantly inhibited with high-fat diets (HFDs) treatment in C57BL/6J mice^96^. On the other hand, the abundance of Parabacteroides was elevated after HFD feeding 4-weeks, which suggested that fatty acid level regulated microbe abundance 97. A strong correlation between gut microbiota and fatty acids has been reported in autistic rats 98. In terms of specific fatty acid, mice injected with valproic acid showed higher abundance of Candidatus_Saccharimonas which suggests a direct link between gut microbiota and fecal metabolites 99. In line with our results, a positive regulation between Parabacteroides and SCFAs has been demonstrated in human fecal microbiota identified by *in vitro* fermentation 100. In addition, high levels of SCFAs promote Parabacteroides bacteria abundance 101.

The essential amino acid L-alanine, which is related to alanine, aspartate, and glutamate metabolism, is an ingredient of protein synthesis and inflammation 102. Alterations can cause an imbalance in the energy metabolism and inflammatory responses involved in stress-induced tumorigenesis 103. In addition to L-alanine, sucrose also showed strong correlations with DEGs and DMGs. A higher total sugar intake increases the risk of breast cancer development was associated with a poorer prognosis after breast cancer diagnosis 104, 105. These findings suggest that carbohydrate and amino acid metabolism are also involved in stress-enhanced tumor growth.

Finally, our DMG/DEG combined analysis identified 2 genes, CDH10 and TBC1D9*,* which were upregulated, and downregulated in stressed tumors, respectively. CDH10 is the gene for cadherin 10, which is associated with autism^106^. CDH10 is shown to be highly mutated in colorectal cancer and associated with better survival^107^. However, our study showed that stress enhances CDH10, and a higher expression of CDH10 in breast, gastric and lung tumors is associated with poorer survival. TBC1D9 is a gene implicated as a long-term survival gene in breast cancer 108, its expression can differentiate TNBC (low) from non-TNBC (high) breast cancer samples, and overexpression leads to better prognosis 109, which is similar to our study. This shows that CDH10 and TBC1D9 are important genes in cancer and stress.

Immune-modulatory AnxA1 possesses multiple functions essential to cancer pathogenesis, including cell proliferation, apoptosis, metastasis, and invasion 110, 111. AnxA1 -deficient mice exhibit reduced tumor growth and enhanced survival *in vivo* 112, and AnxA1 expression is correlated with the high metastatic ability^113^, and ANXA1 modulates the immune response in cancer 114. Recently, evidence has shown that ANXA1 could be used as a possible new therapeutic avenue *via* repairing blood-brain barrier damage in metabolic disease 115. In addition, ANXA1 inhibits obesity, suggesting that ANXA1 improves metabolism in models of metabolically stressed animals 116. Our current study further demonstrated that silencing ANXA1 slows tumor growth and reverses the effects of stress on the changes in the gut microbiome and fatty acid metabolism. This has not been previously reported and may explain the multiple functions of ANXA1 in cancer.

In conclusion, combining multi-omics analysis with machine learning has led us to define the SMMEO axis that results from the stress induction of microbiome-mediated metabolite and epigenetic interactions that eventually lead to the enhancement of tumorigenesis. One limitation of this study is we did not present the direct relationship between microbiota composition / metabolomic profile changes and tumor growth. Other host-related changes may also play an important role in stress. An additional work showing that microbiota depletion or supplementation orally with the metabolites of interest can induce similar changes in tumor growth would benefit this multi-omics study. The novel microbiome and epigenetics markers are potential therapeutic avenues to preventing cancer in women facing stress and thus have enormous potential for improving treatment outcomes.

## Materials and Methods

### Mice, Stress exposure and Bioluminescence imaging of 4T1 tumor

All animal work was approved by the NUS Institutional Animal Care and Use Committee (IACUC) and was in accordance with the National Advisory Committee for laboratory Animal Research (NACLAR) Guidelines (Guidelines on the Care and Use of Animals for Scientific Purposes). BALB/c ANXA1^+/+^ and ANXA1^-/-^ mice were housed under a 12-h light/dark cycle with food and water provided ad libitum under pathogen-free conditions in the animal housing unit of the Comparative Medicine Department of the National University of Singapore.

Mice (ANXA1^+/+^ and ANXA1^-/-^ ) were allocated to the NS control or tube-restraint stress groups (n ≥ 6 per group). Mice in the tube-restraint stress group were placed in a ventilated 50 mL polypropylene conical tube (Corning Inc.) and subjected to restraint stress for two h per day (9:00 AM to 11:00 AM), while the NS group mice were left undisturbed in their cages. Mice in the stress group were restrained for 10 consecutive days. The mice were then subcutaneously injected with 50 µL of stably transfected 4T1-luciferase cell suspension (10^4^ cells per mouse) into the mammary fat pad, and mice were monitored for up to 40 days. The size of the tumours and tissue metastasis was measured and analyzed *via* a bioluminescence imaging assay using the Xenogen IVIS Spectrum Imaging System, along with the machine's software (Comparative Medicine facility at NUS). Mice were injected with 150 µL of luciferin (150mg/kg) VivoGlo™ Luciferin, Promega) intraperitoneal. Tumour volumes were measured manually using a digital caliper and were calculated using the equation: V (mm^3^) = length (mm) × width (mm) × width (mm)/2). Mice were euthanized either at the end of the study or earlier if they displayed significant weight loss, signs of distress, or palpable tumours ≥2.0 cm in diameter.

### 16S ribosomal rDNA sequencing and microbiome analysis

The QIAamp DNA Stool Mini Kit was used for fecal DNA extraction in accordance with the manufacturer's protocols, and the DNA (20-30 ng) was used to generate amplicons libraries. To analyze the taxonomic composition of the bacterial community, amplicons containing the V3 and V4 regions of the prokaryotic 16S RNA gene obtained with the primers were selected for the subsequent pyrosequencing (16S Amplicon PCR Forward Primer: 5'-CCTACGGRRBGCASCAGKVRVGAAT-3'; 16S Amplicon PCR Reverse Primer: 5'- GGACTACNVGGGTWTCTAATCC-3'). Then, the library was purified with magnetic beads, the concentration was detected on a microplate reader, and fragment size was detected by agarose gel electrophoresis. The library was quantified to 10 nM, and PE250/FE300 paired-end sequencing was performed according to the Illumina MiSeq/Novaseq (Illumina, San Diego, CA, USA) manual. MiSeq Control Software (MCS)/Novaseq Control Software (NCS) was used to read sequence information.

After quality filtering, VSEARCH clustering (1.9.6) sequence (sequence similarity was set to 97%) was used for OTU clustering. Then the Ribosomal Database Program (RDP) classifier Bayesian algorithm was used to analyze the OTU species taxonomic representative sequences and the different species classification levels. Shannon and Chao1 analyses and PCA results were displayed based on the sample OTU abundances table.

Intergroup difference analysis at the phylum, class, order, family, genus, and species levels in each cluster were analyzed with the LEfSe method with default settings in Galaxy workflow framework (https://huttenhower.sph.harvard.edu/galaxy/root)^117^. LEfSe used the two-tailed nonparametric Kruskal-Wallis test to evaluate the significance of differences between OTUs in the non-stress and stress groups. A set of pairwise tests was performed using the unpaired Wilcoxon test. Finally, LDA was performed to estimate the effect size of each differentially abundant OTU. The results are expressed as the mean ± SEM. The gut microbiotas were considered significantly different if their differences had a p-value of < 0.05 and an LDA score of |log10| > 2^118^.

The functions from the prokaryotic clades were first predicted using FAPROTAX 119 and visualized by ImageGP (http://www.ehbio.com/ImageGP/index.php/Home/Index/index.html). In addition, we used Phylogenetic Investigation of Communities by Reconstruction of Unobserved States (PICRUSt) software (v1.0.0) 120 to characterize the functional genes in the sample through a comparison of the bacterial composition information obtained from the 16S RNA gene sequencing data. The three following steps were performed in the analysis: (1) The selection of closed-reference OTUs from the obtained 16S rRNA gene sequences, comparison of the sequences with the Greengenes database, and the use of “nearest neighbor” in the database as the reference OTU. (2) Normalizing the OTU abundance matrix using the “nearest neighbor” rRNA gene sequence counts as reference. (3) Calculating and predicting the overall COG functions and pathways based on the “nearest neighbor” function profile derived from the KEGG/COG database. Next, we used the G-test and Fisher exact test to test the significance of the difference between two samples and t-test to compare between two groups.

Disbiome database was used to uncover the microbial composition changes in different kinds of diseases, managed by Ghent University 121. The major taxonomic units in the S group were searched and returned the information related to the experiment (related disease/bacteria, abundance subject/control, control type, detection method, and related literature).

### Metabolomics Analysis

*Chemicals and reagents.* Methanol (MeOH, MS grade), pyridine (anhydrous grade), N-(9-fluorenylmethoxycarbonyl)-glycine (FMOC-glycine), methoxyamine hydrochloride, and N-methyl-N-trimethyl-silyl-trifluoroacetamide (MSTFA) were purchased from Sigma-Aldrich (St. Louis, Missouri, USA). Deionized water was obtained from Milli-Q purification system (Bedford, MA, USA).

*Sample preparation.* The serum/feces sample preparation method was similar to one we used previously 122. Briefly, 40 μL of serum was extracted with 280 μL of cold MeOH (FMOC-glycine as internal standard) to precipitate the proteins. Feces samples were extracted in an ice-cold methanol/water mixture (7:1, FMOC-glycine as internal standard) after by placing in a TissueLyser LT (QIAGEN, Germany) for 10 min and sonicating at 25 Hz for 10 min. The mixture was then centrifuged for 20 min at 14,000 rpm and 5 °C, and the resulting supernatant was filtered through a micro-centrifugal filter (Thermo Scientific 750-µL micro-centrifugal filter, PTFE membrane, 0.2-µm pore size, non-sterile). The sample was vacuumed-dried (CentriVap concentrator, Labconco, USA) and derivatized with 100 µL of methoxyamine-pyridine solution (5 mg/mL) for 2 h at 60 °C, and then with 100 µL of MSTFA (40 °C, 16 h) for GC-MS analysis. Pooled quality control (QC) samples were prepared by mixing a certain amount of each serum/feces sample. The QC samples were analyzed at the beginning, the end, and randomly throughout the whole assay to evaluate the stability and reproducibility of the GC-MS analytical system.

*GC-MS analysis.* GC-MS analysis was performed on an Agilent 7683B Series Injector (Agilent, Santa Clara, CA, USA) coupled with an Agilent 7890A Series gas chromatography system and a 5977B mass detector (Agilent). A fused silica capillary column HP-5MSI (60 m × 0.25 mm i.d., 0.25-μm film thickness) was used, and the injector was kept at 250 °C. A 1-μL aliquot of the sample was pulsed-split injected for each individual analysis. Helium was used as the carrier gas at a constant flow rate of 2 mL/min through the column. The GC oven temperature was maintained at 50 °C for 1 min, then increased to 250 °C at a rate of 8 °C/min, and further increased at 25 °C/min to 300 °C and held for 7 min. The transfer line temperature was kept at 280 °C. Detection was achieved using MS in electron impact mode (70 eV) and full-scan monitoring (m/z 50 to 650). The temperature of the ion source was set at 230 °C, and that of the quadrupole was set at 150 °C.

*Metabolite Identification:* The spectral data were exported as mzData files and pretreated with the online open-source XCMS (https://xcmsonline.scripps.edu/) for peak detection and peak alignment. Peak area normalization in each dataset was calculated by comparison with the internal standard. All identified metabolites were confirmed with standards or matched to the NIST library in GCMS (>80%, p < 0.05). The low relative standard deviation filtration was less than 30%, and the detection frequency was more than 100%.

*Metabolite cluster and metabolomics data analysis:* The identified metabolites were clustered by using Cluster Trend tools in Hiplot (https://hiplot.com.cn), a comprehensive web platform for scientific data visualization. The biomarker analyses, enrichment analysis, integrated metabolic pathway analysis, and network analysis were performed by MetaboAnalyst5.0 123.

### Whole-Genome Bisulfite Sequencing (WGBS)

Next-generation sequencing libraries were constructed following the manufacturer's protocol (Illumina, San Diego, CA, USA). For each tumor sample in the NS and S groups, 1 μg of genomic DNA was randomly fragmented to < 500 bp by sonication (Covaris S220). The fragments were treated with End Prep Enzyme Mix for end repairing, 5′ phosphorylation, and dA-tailing in a single reaction, followed by T-A ligation to add methylated adaptors to both ends. Size selection of the adaptor-ligated DNA was then performed using VAHTS DNA Clean Beads (Vazyme, China), and fragments of ~410 bp (with the insert of approximately 350 bp) were recovered. Then bisulfite conversion was performed using EZ DNA Methylation-Gold™ Kit (Zymo Research, CA, USA). Each sample was then amplified by PCR (ETC811 Thermal Cycler (EASTWIN, China)) for 10 cycles using the P5 (5' AAT GAT ACG GCG ACC ACC GA 3') and P7 (5' CAA GCA GAA GAC GGC ATA CGA GAT 3') primers, with both primers carrying sequences that can anneal with patterned flow cell technology (Illumina) to perform bridge PCR, and P7 primer carrying a six-base index that allows for multiplexing. The PCR products were cleaned up using VAHTS DNA Clean Beads (Vazyme, China), validated using an Qsep100 DNA analyzer (Bioptic, China), and quantified on a Qubit3.0 Fluorometer (Invitrogen, Carlsbad, USA). Then, libraries with different indices were multiplexed and loaded onto an Illumina instrument (NovaSeq 6000) according to the manufacturer's instructions (VAHTS Universal Pro DNA Library Prep Kit for Illumina, Catalog# ND608). Sequencing was carried out using a 2 × 150 paired-end configuration, and image analysis and base calling were conducted using the Illumina pipeline (image control software [HCS/MCS] + OLB + GAPipeline-1.6).

Data analysis with Cutadapt (V1.9.1) was performed to remove the sequences for adaptors, PCR primers, those containing more than 10% N bases, and bases of a quality lower than 20. Bismark (V0.7.12) was used to map clean data to a reference genome and determine the number of mC sites with the coverage2cytosine command. Differentially methylated cytosines between the S and NS groups were detected by methylKit (V0.9.5), and swDMR (V1.0.7) was used to reveal a set of DMRs. Enrichment for target genes was completed with the GO/KEGG database. We obtained 2.48-2.49 × 10^9^ uniquely mapped reads among all the samples to ensure concordant coverage. The average ratios of uniquely mapped reads for the NS and S tumor samples were 91.21% and 91.35%, respectively. The methylation level was calculated as an average of 100,572,417 and 113,164,957 methylated cytosines (mCs) in the NS and S samples, respectively.

### RNA-sequencing

Library preparation for transcriptome sequencing. A total of 1 μg of RNA per tumor sample from the NS and S groups were used as input material for RNA sample preparation. Sequencing libraries were generated using the NEBNext Ultra RNA Library Prep Kit from Illumina, following the manufacturer's recommendations. Briefly, mRNA was purified from total RNA using poly-T oligo-attached magnetic beads. RNA strand fragmentation was carried out using divalent cations under an elevated temperature (42 °C) in NEBNext First Strand Synthesis Reaction Buffer (5X) or using sonication with the Diagenode Bioruptor Pico. Second-strand cDNA synthesis was subsequently performed using DNA Polymerase I and RNase H. In the reaction buffer, the dTTP in the dNTPs was replaced with dUTP. To preferentially select cDNA fragments of 250-300 bp in length, the library fragments were purified with the AMPure XP system (Beckman Coulter, Beverly, USA). Then 3 μL of USER enzyme (NEB, USA) was used with size-selected, adaptor-ligated cDNA at 37 °C for 15 min followed by 5 min at 95 °C before PCR. The PCR was performed with 0.2 µl Phusion High-Fidelity DNA polymerase (Catalog # M0530S, NEB), universal oligo-dt primer [d(T)23VN]. Lastly, the products were purified with the AMPure XP system, and library quality was assessed on the Agilent Bioanalyzer 2100 system.

### Data analysis

After cluster generation, the prepared libraries were sequenced on an Illumina platform, and paired-end reads were generated. Raw data (raw reads) of FASTQ format were first processed using fastp. In this step, clean data (clean reads) were obtained by removing reads containing adapter and poly-N sequences and low-quality reads from the raw data. Paired-end clean reads were aligned to the reference genome using Spliced Transcripts Alignment to a Reference (STAR) software (https://github.com/alexdobin/STAR).

*Differential expression and enrichment analysis.* Differential expression analysis between the NS and S groups (three biological replicates per condition) was performed using the DESeq2 R package. DEGs were identified using the DESeq2 R package based on the read counts at the transcriptional level, which were identified using the absolute log2FC value ≥1 and adjusted p-value of < 0.05 as the statistical standards. DESeq2 provides statistical routines for determining differential expression from digital gene expression data using a model based on the negative binomial distribution. The resulting p-values were adjusted using Benjamini and Hochberg's approach for controlling the false discovery rate (FDR). Genes with an adjusted p-value of < 0.05 found by DESeq2 were assigned as differentially expressed.

GO enrichment analysis and biological pathway analysis were carried out using REACTOME (http://www.reactome.org/PathwayBrowser) and Enrichr (https://maayanlab.cloud/Enrichr/#) 124. MetaboAnalyst 5.0 123 was used for functional pathways, and the default setting was used for enrichment analysis of metabolites. The gene interaction network was created using highly altered hub genes with the GeneMania Prediction Server 125.

### ELISA

Feces and blood samples collected from the NS and S groups were kept at -80 °C till use. The Corticosterone ELISA Kit (Cayman,501320, Ann Arbor, USA) was used to measure corticosterone levels according to the manufacturer's instructions. Samples were extracted before use in ELISA. Briefly, ELISA buffer (100 µl) and a corticosterone standard were added to a 96-wells plate, followed by 50 µl of sample per well at a minimum of two dilutions. After that, 50 µl each of AChE tracer and ELISA antiserum were added to the wells, except for the blank control. The plate was covered with plastic film and incubated overnight at 4 °C. To develop the color reaction, Ellman's reagent was added, followed by wash buffer. Lastly, the color in the plate was read at 412 nm wavelength.

### Correlation and survival analysis

Correlation analysis between the microbiome and metabolites was performed using the OmicStudio tools at https://www.omicstudio.cn/tool/62. R version 3.6.1 (Pearson and spearman, 2019-07-05), ggplot2 (3.3.2), and heatmaply (http://talgalili.github.io/heatmaply/)^126^. Machine learning was performed by h2o-genmodel (https://docs.h2o.ai/h2o/latest-stable/h2o-docs/flow.html). The General Linear Model (GLM is just the sum of the coefficient times the value and then adjusts the threshold. Gradient boosting machine (GBM) and distributed random forests (DRF) takes the mean of 40 forests, then compare with threshold and decide 1 or 0. Correlations in the expression of selected DMGs and DEGs were revealed by the interactive web tool GEPIA 127. Overall survival based on gene expression was analyzed with GEPIA (http://gepia.cancer-pku.cn/) and Kaplan Meier plotter (http://kmplot.com/analysis/index.php?p=background).

### Data Analysis

Samples sizes were calculated using practical meta-analysis effect size calculator (https://www.campbellcollaboration.org/escalc/html/EffectSizeCalculator-Home.php) and G*Power version 3.1.9.7 (https://stats.idre.ucla.edu/other/gpower/#) (power > 0.8)^128^. Three independent experiments were performed, and a two-tailed Student's t-test was used to determine the statistical significance of pro-inflammatory gene expression between the control and treated mice. Pearson correlation coefficient for significant DMGs and DEGs was calculated based on their respective intensity values using the CorrelationCalculator v1.0.1, in MetScape 3.1^129^. One-way and two-way analyses of variance (ANOVA) were used to determine the inter-group differences between more than two groups for one or two variables. The level of statistical significance was taken to be p < 0.05 throughout the study.

## Supplementary Material

Supplementary figures and tables.

## Abbreviations

ANOVA: Analysis of variance
AUC: Area under the curve
SMMEO: Stress-Microbiome-Metabolite-Epigenetic-Oncology
CHG: Chlorhexidine gluconate
COG: Clusters of orthologous genes
DEG: Differentially expressed genes
DMFS: Distant metastasis-free survival
DMR: Differentially methylated regions
DRF: Distributed random forests
FC: Fold change
GBM: Gradient boosting machine
GC-MS: Gas chromatography-mass spectrometry
GLM: Generalized linear modeling
GO: Gene ontology
GSEA: Gene set enrichment analysis
KEGG: Kyoto encyclopedia of genes and genomes
LEfSe: Linear discriminant analysis effect Size
MAPK: Mitogen-activated protein kinase
OUT: Operational taxonomic unit
PCA: Principal component analysis
PFS: Progression free survival
PICRUSt: Phylogenetic investigation of communities by reconstruction of unobserved States
PPS: Post progression survival
TCGA: The cancer genome atlas
WGBS: Whole-genome bisulfite sequencing
