# Supplementary material for: Annexin-A1 deficiency attenuates stress-induced tumor growth via fatty acid metabolism in mice: an Integrated multiple omics analysis on the stress- microbiome-metabolite-epigenetic-oncology (SMMEO) axis
Source: Theranostics. 2022 May 9;12(8):3794–817. doi: 10.7150/thno.68611 (PMC9131274; doi:10.7150/thno.68611)
Supplement: Supplementary file 1 — Supplementary figures and tables. [file thnov12p3794s1.pdf]

Supplementary Materials for

Annexin-A1 deficiency attenuates stress-induced tumor growth via fatty acid metabolism in mice: an Integrated multiple omics analysis on the stress- microbiome-metabolite-epigenetic-oncology (SMMEO) axis

Jianzhou Cui, Karishma Sachaphibulkij, Wen Shiun Teo, Hong Meng Lim, Li Zou, Choon Nam Ong, Rudi Albert, Jinmiao Chen, Lina H. K. Lim*

*Corresponding author. Lina H. K. Lim Email: linalim@nus.edu.sg

This file includes:

Materials and Methods

Figure S1 to S15

Tables S1 to S9

Materials and Methods

Real-time PCR

Cell lysis from WT and Anxa1-A1 knockout 4T1 cells rinsed with 1X PBS. RNA extraction was carried out using the GeneJET RNA Purification Kit (Thermo Fisher Scientific, # K0732) according to the manufacturer's instructions. RNA concentration measured using Nanodrop spectrophotometer (BioFrontier Technology). cDNA synthesis from 1µg of RNA based on two steps protocol. Oligo(dT) and nuclease-free water and RNA were incubated for 5 minutes at 65 °C first. They then followed incubation with a master mix is prepared consisting of dNTPs (ProMega), RevertAid Reverse Transcriptase (Thermo Fisher Scientific), RevertAid RT Buffer at 42 °C for 60 minutes and at 90 °C for 5 minutes. cDNA samples are kept at -20 °C till real-time PCR. GoTaq® qPCR Master Mix (ProMega, # A6010) was used, and a master mix was prepared with each reaction required. Real-time PCR was run according to protocol below: 50 °C 2 minutes, 1 cycle; 95 °C, 10 minutes,1 cycle; 95 °C 15 seconds, 60 °C 1 minutes, 40 cycles; 50 °C 15 seconds, 1 cycle; 60 °C 1 minute, 1 cycle; 95 °C 30 seconds, 1 cycle; 60 °C 15 seconds, 1 cycle. Real-time PCR was run on ABI 7500 real-time PCR system (Applied Biosystems). GAPDH was used as an internal loading control, with minimum duplicates for each gene. Relative expression was calculated using ΔΔCt approximation. Primers used in this part of the experiment were listed below:

*Mus musculus fatty acid synthase (**Fasn**):*

Ms-Fasn-F: ATCATTGGGCACTCCTTGGG

Ms-Fasn-R: CCTCCCAGGACAAACCAACA.

*Mus musculus ATP citrate lyase (**Acly**):*

Ms- Acly-F: TCGACTCCAGCACCCAGTAG,

Ms- Acly-R: TTGGACTTGGGACTGAATCTTGG.

*Mus musculus DNA methyltransferase (cytosine-5) 1 (**Dnmt1**):*

Ms- Dnmt1-F: GGACAGTGACACCCTTTCAGTT,

Ms- Dnmt1-R: TGGGTTTCCGTTTAGTGGGG.

*Mus musculus lysine (K)-specific demethylase 1A **(Lsd1):***

Ms- Lsd1-F: TGGTTGTAACAGGTCTTGGAGG,

Ms- Lsd1-R: GGAACAGCTTGTCCATTGGC.

Western blot

WT and Anxa1-A1 knockout 4T1 cells were harvested, and the concertation of cell lysates was determined using 1X Bradford's Reagent (Bio-Rad Laboratories, # 5000002). Protein samples were prepared by adding 5X Loading Dye with 2-Mercaptoethanol to the sample and boiled at 100 °C for 5 minutes. Samples (20-40 µg) were loaded onto 12% SDS-PAGE gel which was run in 1x running buffer at 100 V for 2.5 hours, followed the transfer procedure. After which, the PVDF membranes were blocked using 3% BSA before primary antibodies, and the blots were incubated overnight at 4°C shaking. The secondary antibodies were added and were washed prior to chemiluminescent detection. ECL Prime Western Blotting Detection Reagent (GE Healthcare Life Sciences) with a GelDoc (Bio-rad Laboratories). The primary antibodies used were ANXA1 (sc-12740, Santa Cruz), Fasn (sc-55580, Santa Cruz), Acly (sc-51726, Santa Cruz), Dnmt1 (sc-271729, Santa Cruz), Lsd1(sc-271720, Santa Cruz) and β-Actin (#4970, Cell signaling). The secondary antibodies used were goat anti-mouse HRP (#sc-2005, Santa Cruz) and goat anti-rabbit HRP (#sc-2030, Santa Cruz).

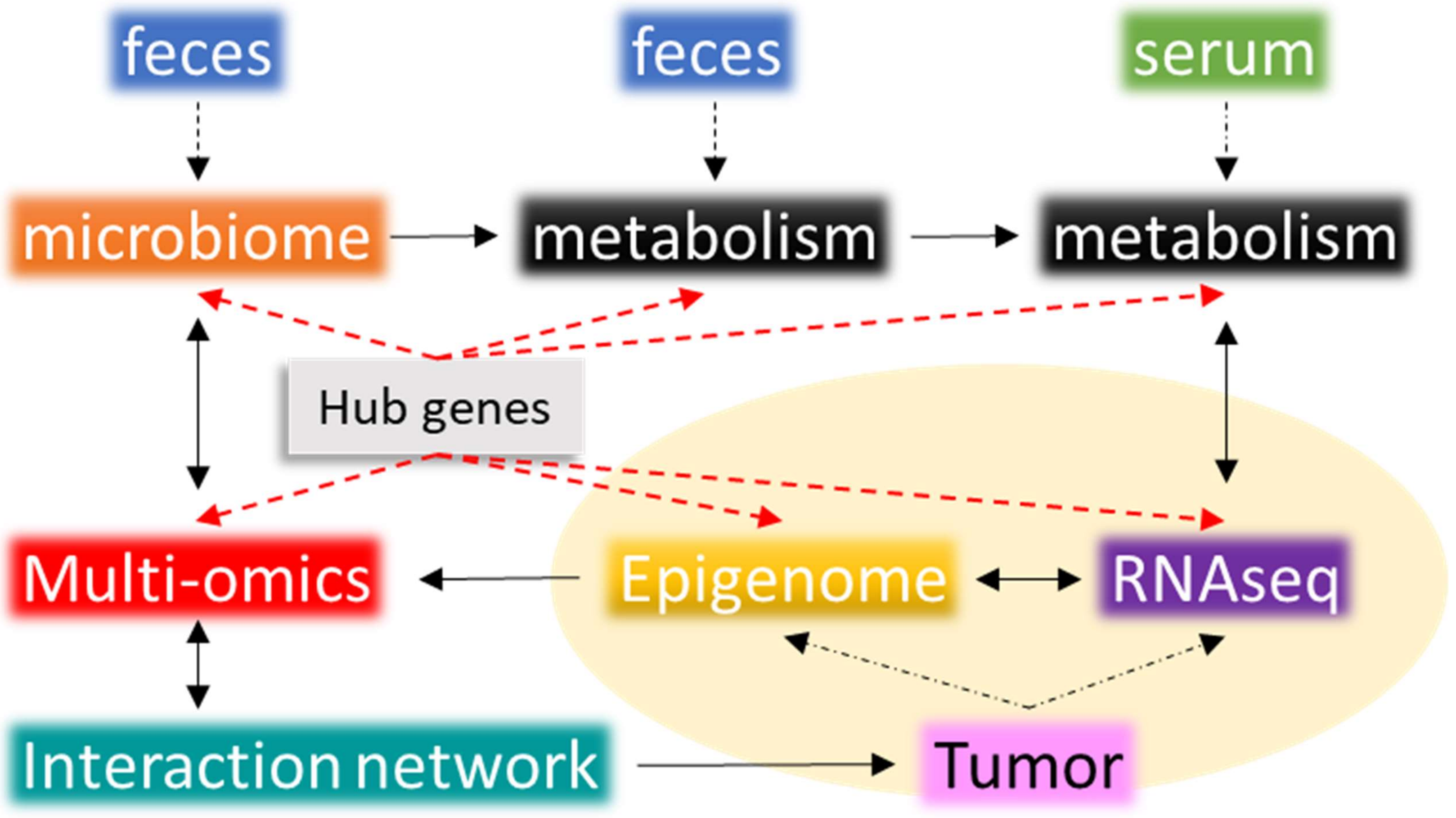

Figure S1. Schematic of experimental design and samples collection protocol. Feces, serum, and tumor samples were used to perform 16S rRNA gene (V3 and V4 regions) metagenomics sequencing, GC-MC metabolism analysis, RNAseq, and Whole-Genome Bisulfite Sequencing (WGBS). The interaction network was constructed, and hub genes were identified via multi-omics systematic analysis and machine learning methods.

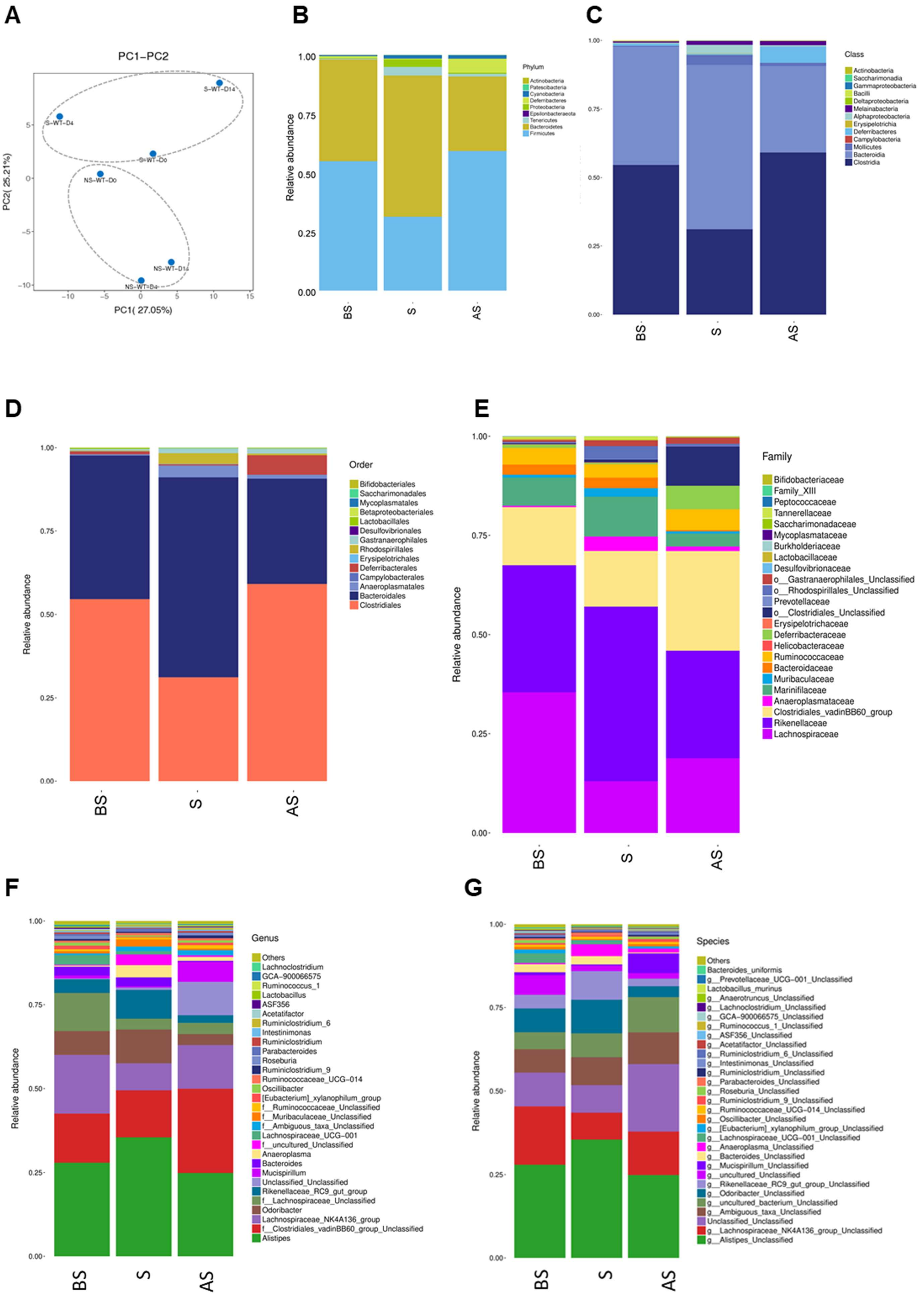
A
PC1−PC2
S−WT−D14
S−WT−D4
S−WT−D0
NS−WT−D0
NS−WT−D14
NS−WT−D4
PC2(25.21%)
PC1(27.05%)
B
Relative abundance
BS
S
AS
Phylum
Actinobacteria
Patescibacteria
Cyanobacteria
Deferribacteres
Proteobacteria
Epsilonbacteraeota
Tenericutes
Bacteroidetes
Firmicutes
C
Class
Actinobacteria
Saccharimonadia
Gammaproteobacteria
Bacilli
Deltaproteobacteria
Melainabacteria
Alphaproteobacteria
Erysipelotrichia
Deferribacteres
Campylobacteria
Mollicutes
Bacteroidia
Clostridia
D
Relative abundance
Order
Bifidobacteriales
Saccharimonadales
Mycoplasmatales
Betaproteobacteriales
Lactobacillales
Desulfovibrionales
Gastranaerophilales
Rhodospirillales
Erysipelotrichales
Deferribacterales
Campylobacterales
Anaeroplasmatales
Bacteroidales
Clostridiales
E
Relative abundance
Family
Bifidobacteriaceae
Family_XIII
Peptococcaceae
Tannerellaceae
Saccharimonadaceae
Mycoplasmataceae
Burkholderiaceae
Lactobacillaceae
Desulfovibrionaceae
o__Gastranaerophilales_Unclassified
o__Rhodospirillales_Unclassified
Prevotellaceae
o__Clostridiales_Unclassified
Erysipelotrichaceae
Deferribacteraceae
Helicobacteraceae
Ruminococcaceae
Bacteroidaceae
Muribaculaceae
Marinifilaceae
Anaeroplasmataceae
Clostridiales_vadinBB60_group
Rikenellaceae
Lachnospiraceae
F
Relative abundance
Genus
Others
Lachnoclostridium
GCA−900066575
Ruminococcus_1
Lactobacillus
ASF356
Acetatifactor
Ruminiclostridium_6
Intestinimonas
Ruminiclostridium
Parabacteroides
Roseburia
Ruminiclostridium_9
Ruminococcaceae_UCG−014
Oscillibacter
[Eubacterium]_xylanophilum_group
f__Ruminococcaceae_Unclassified
f__Muribaculaceae_Unclassified
f__Ambiguous_taxa_Unclassified
Lachnospiraceae_UCG−001
f__uncultured_Unclassified
Anaeroplasma
Bacteroides
Mucispirillum
Unclassified_Unclassified
Rikenellaceae_RC9_gut_group
f__Lachnospiraceae_Unclassified
Odoribacter
Lachnospiraceae_NK4A136_group
f__Clostridiales_vadinBB60_group_Unclassified
Alistipes
G
Relative abundance
Species
Others
Bacteroides_uniformis
g__Prevotellaceae_UCG−001_Unclassified
Lactobacillus_murinus
g__Anaerotruncus_Unclassified
g__Lachnoclostridium_Unclassified
g__GCA−900066575_Unclassified
g__Ruminococcus_1_Unclassified
g__ASF356_Unclassified
g__Acetatifactor_Unclassified
g__Ruminiclostridium_6_Unclassified
g__Intestinimonas_Unclassified
g__Ruminiclostridium_Unclassified
g__Parabacteroides_Unclassified
g__Roseburia_Unclassified
g__Ruminiclostridium_9_Unclassified
g__Ruminococcaceae_UCG−014_Unclassified
g__Oscillibacter_Unclassified
g__[Eubacterium]_xylanophilum_group_Unclassified
g__Lachnospiraceae_UCG−001_Unclassified
g__Anaeroplasma_Unclassified
g__Bacteroides_Unclassified
g__Mucispirillum_Unclassified
g__uncultured_Unclassified
g__Rikenellaceae_RC9_gut_group_Unclassified
g__Odoribacter_Unclassified
g__uncultured_bacterium_Unclassified
g__Ambiguous_taxa_Unclassified
Unclassified_Unclassified
g__Lachnospiraceae_NK4A136_group_Unclassified
g__Alistipes_Unclassified

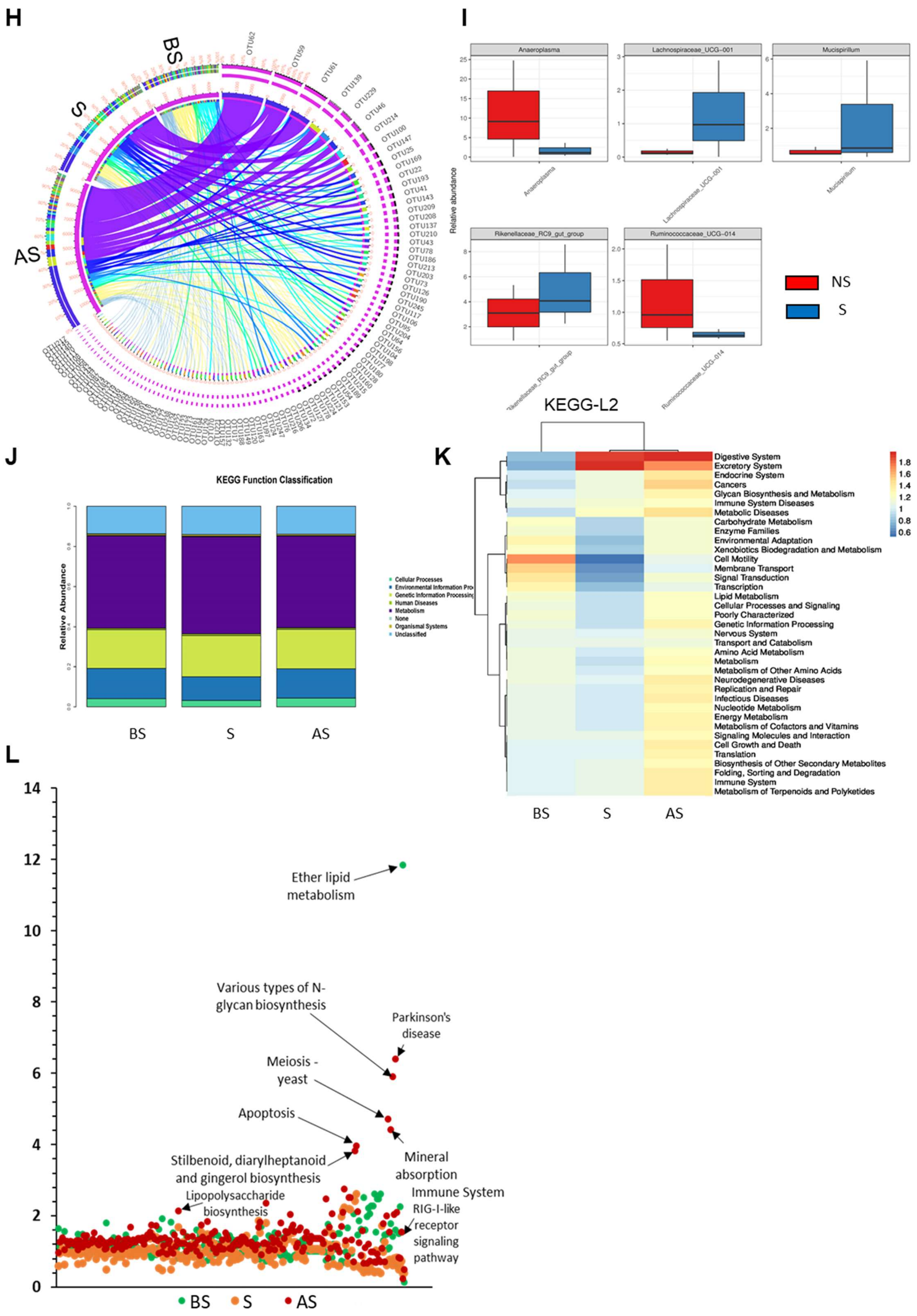

Figure S2. Stress alters gut microbiome composition. (A) Beta Diversity between the non-stressed and stressed group. **(B-G)** Taxonomic information based on 16S rRNA gene sequences (classified with a confidence threshold of 80%) and expressed as the fraction of total sequences at three stages. Relative abundance bar plots represent the bacterial composition in feces microbiota at the phylum, class, order, family, genus, species levels, respectively. Each legend box shows the top 10-40 classified taxa among the whole proportion. **(H)** Circos analysis results of the corresponding abundance relationship between three stress stages and bacterial communities. **(I)** Relative abundance of five strains between NS and S groups. The x-axis indicates the names of the five strains, and the y-axis provides the relative abundance of each genus. The multiple hypothesis tests and the false discovery rate of rare frequency data were performed to evaluate the significance of the difference between NS and S groups at $P < 0.05$. **(J-L)** Summary of feces microbiome of COG Function Classification and KEGG function classification (Level 1 and level3) of assembled contigs at three stages when stressed group compared with the non-stressed group.

Table S1. OTU distribution pattern in fecal microbiome of stressed mice

OTU_ID	Taxonomy
OTU62	k__Bacteria;p__Firmicutes;c__Clostridia;o__Clostridiales
OTU59	k__Bacteria;p__Firmicutes;c__Clostridia;o__Clostridiales;f__Clostridiales_vadinBB60_group;g__uncultured_bacterium;s__uncultured_bacterium
OTU46	k__Bacteria;p__Firmicutes;c__Clostridia;o__Clostridiales;f__Clostridiales_vadinBB60_group
OTU61	k__Bacteria;p__Proteobacteria;c__Alphaproteobacteria;o__Rhodospirillales;f__uncultured;g__Ambiguous_taxa;s__Ambiguous_taxa
OTU229	k__Bacteria;p__Firmicutes;c__Clostridia;o__Clostridiales;f__Clostridiales_vadinBB60_group
OTU214	k__Bacteria;p__Cyanobacteria;c__Melainabacteria;o__Gastranaerophilales;f__Ambiguous_taxa;g__Ambiguous_taxa;s__Ambiguous_taxa
OTU139	k__Bacteria;p__Firmicutes;c__Clostridia;o__Clostridiales;f__Clostridiales_vadinBB60_group;g__uncultured_bacterium;s__uncultured_bacterium
OTU147	k__Bacteria;p__Firmicutes;c__Clostridia;o__Clostridiales;f__Clostridiales_vadinBB60_group
OTU100	k__Bacteria;p__Firmicutes;c__Clostridia;o__Clostridiales;f__Clostridiales_vadinBB60_group;g__uncultured_bacterium;s__uncultured_bacterium

Table S2. Microbial compositional changes in WT mice in relation to different diseases

	Disease	Organism	Qualitative outcome[a]	Response	Method
**Clostridiales**	Periodontal disease	*Johnsonella*	up	Log2	MiSeq sequencing
	Type 2 diabetes	*Faecalibacterium prausnitzii*	down	Log10	qPCR
	Aggressive periodontitis	*Peptostreptococcaceae*	down	% (abundancy)	16S rDNA pyrosequencing
	Autism spectrum disorders	*Peptoniphilus*	up	% (abundancy)	16S rRNA sequencing
	Autoimmune polyendocrine syndrome type 1	*Catonella*	up	% (abundancy)	16S rRNA sequencing
	Crohn's Disease	*Ruminococcus gnavus*	up	Median intensity	Denaturing gradient gel electrophoresis
	Hepatocellular cancer	*Anaerostipes*	up	% (abundancy)	16S rRNA sequencing
	Obesity	*Catonella morbi*	up	% (abundancy)	Metagenomic sequencing
**Rhodospirillales**	Colorectal cancer	*Acidocella*	down	% (abundancy)	16S rRNA sequencing
	Inflammatory bowel disease	*Gluconobacter oxydans*	up	% (prevalence)	16S rRNA sequencing
	Type 1 diabetes	*Thalassospira*	up	% (prevalence)	16S rRNA sequencing
	Breast cancer	*Gluconacetobacter*	up	% (prevalence)	16S rRNA sequencing
	Parkinson's disease	*Enhydrobacter*	up	B-value	16S rRNA sequencing
**Gastranaerophilales**	Type 1 diabetes	*Eubacterium eligens*	down	% (abundancy)	16S rRNA sequencing
	Type 2 diabetes	*Shuttleworthia*	up	% (abundancy)	16S rRNA sequencing
	Non-alcoholic fatty liver disease	*Ruminococcus*	down	% (abundancy)	16S rRNA pyrosequencing
	Pulmonary tuberculosis	*Roseburia*	down	% (abundancy)	16S rRNA sequencing
	Coronary artery disease	*Roseburia*	down	% (abundancy)	16S rRNA sequencing
	Obesity	*Lachnospira*	up	% (abundancy)	16S rDNA pyrosequencing

[a] up: Elevated down: Reduced

Table S3. Differential metabolites in feces between non-stressed and stressed groups at three sampling points

Feces: metabolites	NIST match	m/z[a]	RT[b] (min)	RFQC_CV (n=7; %)	FC[c] (S vs NS)		
					D0	D4	D14
Lactic acid	87	117.1	10.76	4	0.46	1.11	1.92
Alanine1	STD	116.1	11.54	6	0.80	2.55	0.93
Alanine2	STD	190.1	11.54	5	0.87	2.45	1.07
Valine	STD	144.1	13.72	14	1.07	1.89	0.65
Urea	94	147.1	13.96	9	2.16	2.39	1.41
Leucine/Isoleucine1	STD	158.2	14.71	7	1.31	1.68	0.63
Glycerol1	93	205.1	14.76	11	0.69	1.61	1.14
Glycerol2	93	218.1	14.76	8	0.62	1.58	1.18
Phosphonic acid	84	299.1	14.81	11	0.85	1.25	0.93
Leucine/Isoleucine2	STD	158.1	15.12	6	1.12	1.87	0.59
Proline	STD	142.1	15.18	4	1.44	2.02	0.73
Glycine	STD	174.1	15.35	4	1.18	2.19	0.74
Serine	STD	218.1	16.27	10	0.70	1.43	1.18
Threonine	STD	219.1	16.73	6	1.03	1.72	0.82
β-Alanine	STD	248.2	17.36	8	0.91	1.25	1.14
L-Methionine	STD	176.1	18.81	9	1.55	0.85	0.86
Aspartic acid	STD	232.1	18.81	3	2.21	1.42	0.27
L-5-Oxoproline	89	156.1	18.92	7	1.48	1.55	0.62
Ornithine	STD	142.1	20.25	7	1.67	3.09	0.37
Phenylalanine	STD	218.1	20.48	7	1.01	1.81	0.72
Xylose/Arabinose/Ribose1	84	103.1	21.01	6	0.58	1.44	0.82
Xylose/Arabinose/Ribose2	84	217.1	21.05	7	0.55	1.63	0.88
Arabinose/Xylose/Ribose	84	307.2	21.10	8	0.55	1.59	0.87
Myristic acid	STD	285.2	23.28	8	0.63	1.28	1.77
Mannose	STD	319.2	24.17	6	0.73	1.52	0.67
Glucose1	STD	147.1	24.32	6	0.78	1.66	0.45
Glucose2	STD	160.1	24.32	7	0.76	1.67	0.42
Glucose3	STD	205.1	24.32	6	0.79	1.67	0.47
Glucose4	STD	319.2	24.32	5	0.77	1.65	0.50
Tyrosine	STD	218.1	24.63	6	0.98	1.78	0.53
Manitol	STD	319.2	24.74	10	1.04	2.27	0.94
Sorbitol	STD	205.1	24.84	3	0.66	1.84	1.01
Palmitoleic acid	STD	311.3	25.52	3	0.54	1.03	1.37
Xanthine	84	294.2	25.67	5	0.65	2.73	0.72
Palmitic acid	STD	313.3	25.72	4	0.63	0.78	1.81
Myo-Inositol1	STD	305.3	26.71	5	0.70	2.30	1.12
Myo-Inositol2	STD	318.2	26.71	3	0.66	2.40	1.45
Linoleic acid	STD	337.3	27.85	5	0.52	0.63	1.55
Oleic acid	STD	339.3	28.05	5	0.53	1.03	1.55
Linolenic acid	STD	335.3	27.96	3	0.66	0.82	1.18
Stearic acid1	STD	341.3	28.27	10	0.64	0.97	1.78
Stearic acid2	STD	356.3	28.27	14	0.62	0.97	1.90
L-Tryptophan	STD	202.1	28.32	9	0.93	1.43	0.90
Gondoic acid	STD	367.3	31.23	5	0.48	0.95	1.74
Sucrose	STD	361.2	34.40	7	0.39	1.54	0.89

[a]m/z: mass value

[b]RT: retention time

[c] The ratio of the relative value (vs. internal standard) of metabolites in the stressed (S) and non-stressed (NS) groups of samples; FC > 1 indicates upregulated metabolites, and FC < 1 indicates down-regulated metabolites.

Table S4. Differential metabolites in serum between non-stressed and stressed groups at three sampling points

Serum metabolites	NIST match	m/z[a]	RT[b] (min)	RSQC_CV (n=10; %)	FC[c] (S vs NS)		
					D0	D4	D14
Lactic acid	87	117.1	10.76	12	2.26	1.95	0.99
Alanine1	STD	116.1	11.54	8	3.07	2.28	1.30
Alanine2	STD	190.1	11.54	10	1.58	1.69	1.22
Valine	STD	144.1	13.72	12	2.37	9.00	1.79
Urea	94	147.1	13.96	10	1.74	3.55	0.98
Leucine/Isoleucine1	STD	158.2	14.71	7	2.66	7.98	2.31
Glycerol1	93	205.1	14.76	12	1.17	1.69	1.27
Glycerol2	93	218.1	14.76	10	1.33	2.47	1.43
Phosphonic acid	84	299.1	14.81	14	0.94	2.63	1.36
Leucine/Isoleucine2	STD	158.1	15.12	9	0.79	1.45	0.66
Proline	STD	142.1	15.18	11	2.35	2.32	1.90
Glycine	STD	174.1	15.35	9	0.70	0.95	0.77
Serine	STD	218.1	16.27	13	1.14	0.93	0.80
Threonine	STD	218.1	16.73	10	1.63	2.80	0.61
L-Methionine	STD	176.1	18.81	10	2.50	3.00	0.77
Aspartic acid	STD	232.1	18.81	9	2.25	3.18	2.00
L-5-Oxoproline	89	156.1	18.92	6	1.78	3.24	1.57
Ornithine	STD	142.1	20.25	13	1.05	1.14	0.62
Phenylalanine	STD	218.1	20.48	12	1.70	2.15	0.98
Lauric acid	STD	257.2	20.64	13	1.34	1.22	1.25
L-Ornithine	STD	142.1	23.15	11	1.10	1.50	0.94
Citric acid1	STD	273.1	23.18	12	0.53	1.70	0.48
Citric acid2	STD	347.2	23.18	15	0.68	2.00	0.38
Myristic acid	STD	285.2	23.28	12	2.07	1.71	2.59
Mannose	STD	319.2	24.17	11	1.52	7.00	1.96
Glucose1	STD	147.1	24.32	9	1.26	3.42	1.31
Glucose2	STD	160.1	24.32	9	1.49	4.02	1.45
Glucose3	STD	205.1	24.32	7	1.13	4.24	1.55
Glucose4	STD	319.2	24.32	8	1.32	5.18	1.43
Tyrosine	STD	218.1	24.63	11	5.71	8.42	5.12
Sorbitol	STD	205.1	24.84	14	0.76	1.07	1.14
Palmitoleic acid	STD	311.3	25.57	12	0.34	0.40	0.66
Xanthine	84	294.2	25.67	13	0.44	1.33	0.50
Palmitic acid	STD	313.3	25.72	12	0.66	0.81	0.85
Myo-Inositol1	STD	305.3	26.71	10	0.93	1.16	0.69
Myo-Inositol2	STD	318.2	26.71	11	0.97	0.93	1.19
Linoleic acid	STD	337.3	27.85	13	0.80	1.05	1.37
Oleic acid	STD	339.3	27.91	10	1.17	0.90	1.65
Linolenic acid	STD	335.3	27.96	13	1.20	1.33	1.33
Stearic acid	STD	341.3	28.27	19	1.04	1.51	1.04
L-Tryptophan	STD	202.1	28.32	8	0.94	0.94	0.73
Gondoic acid	STD	367.3	31.23	13	0.75	0.70	1.31
Docosahexaenoic acid	STD	117.1	33.05	16	0.92	2.00	0.78
Sucrose	STD	361.2	34.40	12	1.00	0.67	1.00

[a]m/z: mass value

[b]RT: retention time

[c] The ratio of the relative value (vs. internal standard) of metabolites in the stressed (S) and non-stressed (NS) groups of samples; FC > 1 indicates upregulated metabolites, and FC < 1 indicates down-regulated metabolites.

Table S5. Correlation among taxonomic units of fecal microbiome and fatty acids

OTU_ID	Relationship	Taxonomy
OTU112	positive	p__Patescibacteria;c__Saccharimonadia;o__Saccharimonadales;f__Saccharimonadaceae;g__Candidatus_Saccharimonas;s__uncultured_bacterium
OTU122	positive	p__Firmicutes;c__Clostridia;o__Clostridiales;f__Lachnospiraceae;g__uncultured;s__Clostridium_sp._Culture-27
OTU128	positive	p__Firmicutes;c__Clostridia;o__Clostridiales;f__Lachnospiraceae;g__Lachnospiraceae_NK4A136_group
OTU142	positive	p__Firmicutes;c__Clostridia;o__Clostridiales;f__Lachnospiraceae;g__Blautia;s__Lachnospiraceae_bacterium_609
OTU188	positive	p__Firmicutes;c__Clostridia;o__Clostridiales;f__Clostridiales_vadinBB60_group;g__uncultured_bacterium;s__uncultured_bacterium
OTU190	positive	p__Firmicutes;c__Clostridia;o__Clostridiales;f__Lachnospiraceae;g__Lachnospiraceae_NK4A136_group;s__uncultured_bacterium
OTU199	positive	p__Firmicutes;c__Clostridia;o__Clostridiales;f__Ruminococcaceae;g__Ruminiclostridium_5;s__uncultured_bacterium
OTU223	positive	p__Firmicutes;c__Clostridia;o__Clostridiales;f__Ruminococcaceae;g__Candidatus_Soleaferrea
OTU3	positive	p__Bacteroidetes;c__Bacteroidia;o__Bacteroidales;f__Rikenellaceae;g__Alistipes
OTU34	positive	p__Deferribacteres;c__Deferribacteres;o__Deferribacterales;f__Deferribacteraceae;g__Mucispirillum
OTU37	positive	p__Firmicutes;c__Clostridia;o__Clostridiales;f__Lachnospiraceae;g__uncultured;s__uncultured_bacterium
OTU46	positive	p__Firmicutes;c__Clostridia;o__Clostridiales;f__Clostridiales_vadinBB60_group
OTU72	positive	p__Bacteroidetes;c__Bacteroidia;o__Bacteroidales;f__Tannerellaceae;g__Parabacteroides
OTU108	negative	p__Firmicutes;c__Clostridia;o__Clostridiales;f__Lachnospiraceae;g__Tyzzerella;s__uncultured_bacterium
OTU118	negative	p__Firmicutes;c__Clostridia;o__Clostridiales;f__Ruminococcaceae;g__uncultured;s__unidentified
OTU13	negative	p__Bacteroidetes;c__Bacteroidia;o__Bacteroidales;f__Bacteroidaceae;g__Bacteroides;s__Ambiguous_taxa
OTU149	negative	p__Firmicutes;c__Clostridia;o__Clostridiales;f__Ruminococcaceae;g__Ruminiclostridium_6
OTU16	negative	p__Firmicutes;c__Clostridia;o__Clostridiales;f__Lachnospiraceae;g__Lachnospiraceae_UCG-001;s__uncultured_bacterium
OTU165	negative	p__Firmicutes;c__Clostridia;o__Clostridiales;f__Ruminococcaceae;g__Butyricicoccus;s__Ambiguous_taxa
OTU198	negative	p__Bacteroidetes;c__Bacteroidia;o__Bacteroidales;f__Bacteroidaceae;g__Bacteroides;s__Bacteroides_uniformis
OTU207	negative	p__Bacteroidetes;c__Bacteroidia;o__Bacteroidales;f__Tannerellaceae;g__Parabacteroides;s__uncultured_bacterium
OTU215	negative	p__Firmicutes;c__Clostridia;o__Clostridiales;f__Ruminococcaceae;g__Ruminococcaceae_UCG-014;s__uncultured_rumen_bacterium
OTU219	negative	p__Bacteroidetes;c__Bacteroidia;o__Bacteroidales;f__Muribaculaceae;g__uncultured_bacterium;s__uncultured_bacterium
OTU47	negative	p__Firmicutes;c__Clostridia;o__Clostridiales;f__Ruminococcaceae;g__Intestinimonas;s__uncultured_bacterium
OTU6	negative	p__Tenericutes;c__Mollicutes;o__Anaeroplasmatales;f__Anaeroplasmataceae;g__Anaeroplasma;s__uncultured_bacterium
OTU74	negative	p__Firmicutes;c__Clostridia;o__Clostridiales;f__Lachnospiraceae;g__Roseburia;s__Clostridium_sp._Clone-44
OTU81	negative	p__Firmicutes;c__Clostridia;o__Clostridiales;f__Ruminococcaceae;g__Ruminiclostridium;s__uncultured_bacterium

A

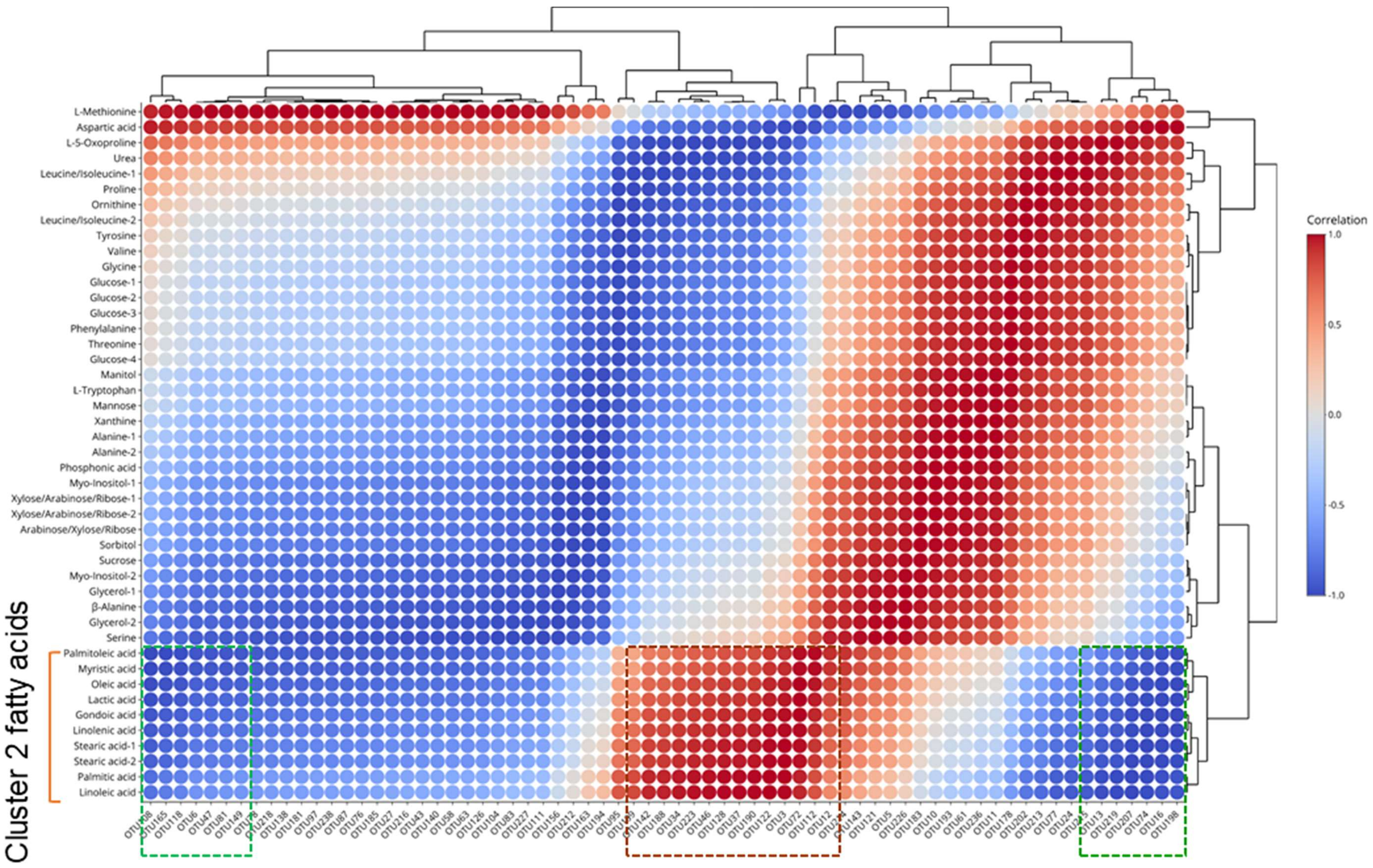

B

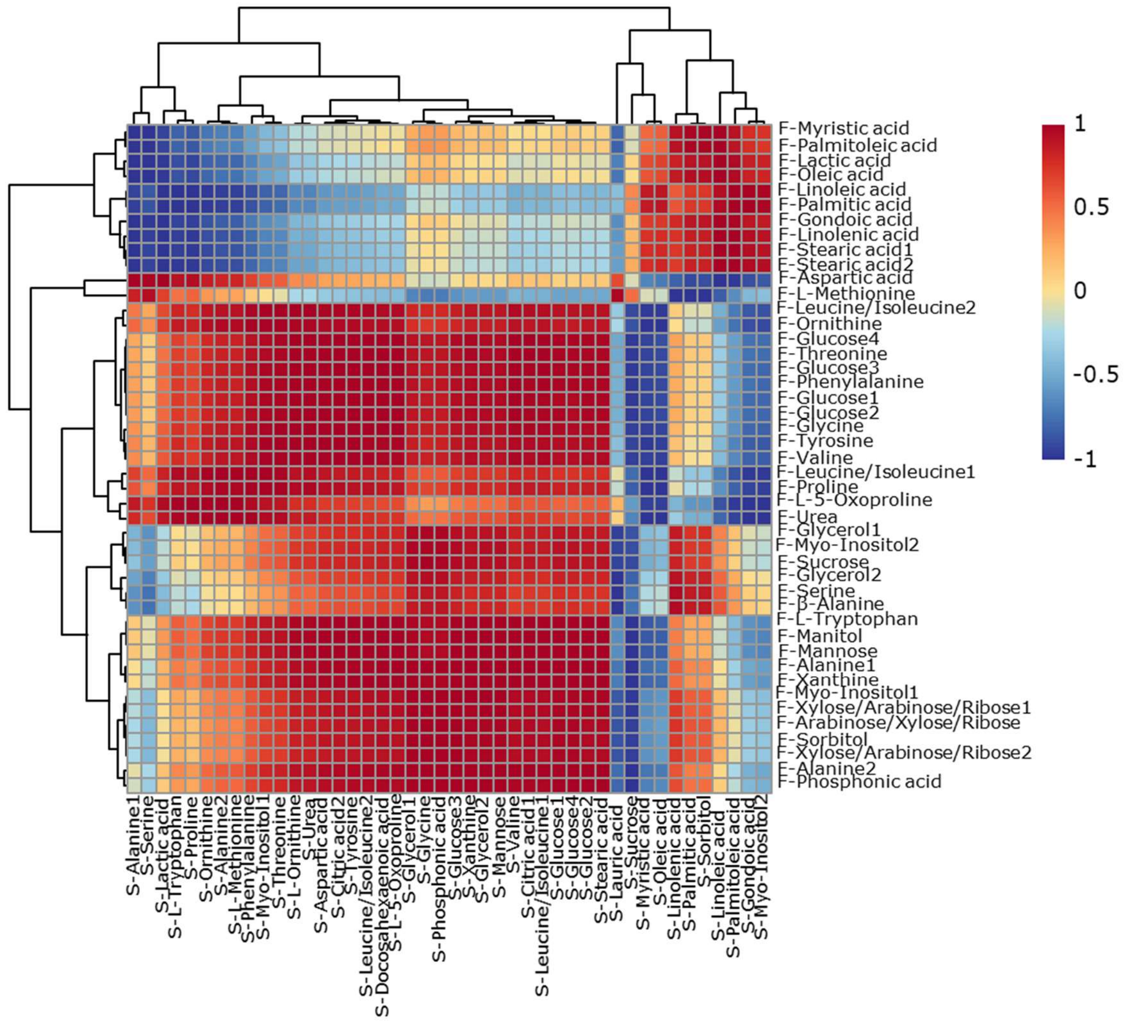

Figure S3. Correlation analyis. (A) Correlation between fecal microbiota and fecal metabolites under stress exposure. The color indicates the Spearman correlation coefficient distribution. Red represents a significant positive correlation ($p < 0.05$), green represents a significantly negative correlation ($p < 0.05$) **(B)**The Pearson correlation coefficients were calculated for log2 transformed ratios of the metabolites between feces and serum sample in the stressed group compared with non-stressed groups.

Table S6. Correlation among fecal and serum fatty acids metabolites

Feces	Serum	Correlation coefficient-rho	Relation
F-Gondoic acid	S-Myo-Inositol2	0.875456071	positive
F-Gondoic acid	S-Sorbitol	0.88205238	positive
F-Gondoic acid	S-Palmitic acid	0.884070967	positive
F-Gondoic acid	S-Gondoic acid	0.896855856	positive
F-Gondoic acid	S-Palmitoleic acid	0.978161733	positive
F-Gondoic acid	S-Linoleic acid	0.997396232	positive
F-Linoleic acid	S-Myristic acid	0.869682552	positive
F-Linoleic acid	S-Oleic acid	0.893170939	positive
F-Linoleic acid	S-Linoleic acid	0.939096188	positive
F-Linoleic acid	S-Myo-Inositol2	0.974617318	positive
F-Linoleic acid	S-Gondoic acid	0.983922284	positive
F-Linoleic acid	S-Palmitoleic acid	0.997602825	positive
F-Linolenic acid	S-Palmitic acid	0.853221516	positive
F-Linolenic acid	S-Myo-Inositol2	0.903878077	positive
F-Linolenic acid	S-Gondoic acid	0.922682264	positive
F-Linolenic acid	S-Palmitoleic acid	0.989214884	positive
F-Linolenic acid	S-Linoleic acid	0.990961788	positive
F-Myristic acid	S-Linolenic acid	0.903971946	positive
F-Myristic acid	S-Palmitoleic acid	0.906086352	positive
F-Myristic acid	S-Sorbitol	0.965585477	positive
F-Myristic acid	S-Palmitic acid	0.966695352	positive
F-Myristic acid	S-Linoleic acid	0.987964318	positive
F-Oleic acid	S-Linolenic acid	0.858913605	positive
F-Oleic acid	S-Sorbitol	0.936271129	positive
F-Oleic acid	S-Palmitic acid	0.937773557	positive
F-Oleic acid	S-Palmitoleic acid	0.942410269	positive
F-Oleic acid	S-Linoleic acid	0.998231541	positive
F-Palmitic acid	S-Myristic acid	0.861027811	positive
F-Palmitic acid	S-Oleic acid	0.885270777	positive
F-Palmitic acid	S-Linoleic acid	0.944891283	positive
F-Palmitic acid	S-Myo-Inositol2	0.970605429	positive
F-Palmitic acid	S-Gondoic acid	0.980691029	positive
F-Palmitic acid	S-Palmitoleic acid	0.998649162	positive
F-Palmitoleic acid	S-Palmitoleic acid	0.895225315	positive
F-Palmitoleic acid	S-Linolenic acid	0.914379678	positive
F-Palmitoleic acid	S-Sorbitol	0.971786108	positive
F-Palmitoleic acid	S-Palmitic acid	0.97279173	positive
F-Palmitoleic acid	S-Linoleic acid	0.983788272	positive
F-Stearic acid1	S-Myo-Inositol2	0.916807845	positive
F-Stearic acid1	S-Gondoic acid	0.934282895	positive
F-Stearic acid1	S-Linoleic acid	0.986284639	positive
F-Stearic acid1	S-Palmitoleic acid	0.993309791	positive
F-Stearic acid2	S-Myo-Inositol2	0.923331906	positive
F-Stearic acid2	S-Gondoic acid	0.940091718	positive
F-Stearic acid2	S-Linoleic acid	0.983398681	positive
F-Stearic acid2	S-Palmitoleic acid	0.995095441	positive
F-Gondoic acid	S-Alanine1	-0.996507092	negtive
F-Gondoic acid	S-Lactic acid	-0.990088181	negtive
F-Gondoic acid	S-Serine	-0.962267965	negtive
F-Gondoic acid	S-Proline	-0.949870261	negtive
F-Gondoic acid	S-L-Tryptophan	-0.925006613	negtive
F-Gondoic acid	S-Ornithine	-0.859114022	negtive
F-Linoleic acid	S-Proline	-0.999225926	negtive
F-Linoleic acid	S-L-Tryptophan	-0.993832888	negtive
F-Linoleic acid	S-Lactic acid	-0.990532456	negtive
F-Linoleic acid	S-Ornithine	-0.966740322	negtive
F-Linoleic acid	S-L-Methionine	-0.950956747	negtive
F-Linoleic acid	S-Alanine2	-0.949212986	negtive
F-Linoleic acid	S-Alanine1	-0.935108159	negtive
F-Linoleic acid	S-Phenylalanine	-0.881100727	negtive
F-Linoleic acid	S-Serine	-0.850319063	negtive
F-Linolenic acid	S-Lactic acid	-0.996917173	negtive
F-Linolenic acid	S-Alanine1	-0.989364312	negtive
F-Linolenic acid	S-Proline	-0.967510629	negtive
F-Linolenic acid	S-L-Tryptophan	-0.946890598	negtive
F-Linolenic acid	S-Serine	-0.943436476	negtive
F-Linolenic acid	S-Ornithine	-0.889343389	negtive
F-Linolenic acid	S-L-Methionine	-0.86243403	negtive
F-Linolenic acid	S-Alanine2	-0.859591419	negtive
F-Myristic acid	S-Serine	-0.998843707	negtive
F-Myristic acid	S-Alanine1	-0.989667273	negtive
F-Myristic acid	S-Lactic acid	-0.932905655	negtive
F-Myristic acid	S-Proline	-0.854888669	negtive
F-Oleic acid	S-Alanine1	-0.998845622	negtive
F-Oleic acid	S-Serine	-0.989661559	negtive
F-Oleic acid	S-Lactic acid	-0.963081493	negtive
F-Oleic acid	S-Proline	-0.900605655	negtive
F-Oleic acid	S-L-Tryptophan	-0.867121199	negtive
F-Palmitic acid	S-Proline	-0.99839748	negtive
F-Palmitic acid	S-Lactic acid	-0.992755622	negtive
F-Palmitic acid	S-L-Tryptophan	-0.991769538	negtive
F-Palmitic acid	S-Ornithine	-0.962178972	negtive
F-Palmitic acid	S-L-Methionine	-0.945472664	negtive
F-Palmitic acid	S-Alanine2	-0.943637448	negtive
F-Palmitic acid	S-Alanine1	-0.941088785	negtive
F-Palmitic acid	S-Phenylalanine	-0.872801489	negtive
F-Palmitic acid	S-Serine	-0.85928126	negtive
F-Palmitoleic acid	S-Serine	-0.999733433	negtive
F-Palmitoleic acid	S-Alanine1	-0.985773182	negtive
F-Palmitoleic acid	S-Lactic acid	-0.923610607	negtive
F-Stearic acid1	S-Lactic acid	-0.998882518	negtive
F-Stearic acid1	S-Alanine1	-0.984334291	negtive
F-Stearic acid1	S-Proline	-0.974940593	negtive
F-Stearic acid1	S-L-Tryptophan	-0.95647879	negtive
F-Stearic acid1	S-Serine	-0.932612147	negtive
F-Stearic acid1	S-Ornithine	-0.903200769	negtive
F-Stearic acid1	S-L-Methionine	-0.877833947	negtive
F-Stearic acid1	S-Alanine2	-0.87514315	negtive
F-Stearic acid2	S-Lactic acid	-0.999531153	negtive
F-Stearic acid2	S-Alanine1	-0.981261075	negtive
F-Stearic acid2	S-Proline	-0.978510744	negtive
F-Stearic acid2	S-L-Tryptophan	-0.961206375	negtive
F-Stearic acid2	S-Serine	-0.926471929	negtive
F-Stearic acid2	S-Ornithine	-0.910224562	negtive
F-Stearic acid2	S-L-Methionine	-0.885689845	negtive
F-Stearic acid2	S-Alanine2	-0.883081019	negtive

Table S7. Differentially methylated region (DMR) of tumor samples (stressed vs non-stressed group)

GeneSymbol	Log2 (FC) (N /NS)	Corrected p-value	Feature
Zxdb	-5.096215333	9.13E-19	ncRNA_exonic
Tulp4	-4.981679629	1.55E-53	ncRNA_intronic
Lurap1l	-4.701111268	1E-11	upstream
Psg29	-4.499466251	4.07E-05	intergenic
Lurap1l	-4.377086037	1E-11	ncRNA_exonic
Eva1a	-4.264348601	1E-13	ncRNA_intronic
Lurap1l	-4.218415443	1E-11	ncRNA_exonic
Lurap1l	-4.217217091	1E-11	upstream
Gm3500	-4.066443276	7.21E-15	ncRNA_intronic
Eva1a	-3.853417098	1E-13	ncRNA_intronic
Gm21699	-3.798701345	1.03E-13	intergenic
Eva1a	-3.773287448	1E-13	ncRNA_intronic
Pih1d3	-3.740240714	0.002375431	intergenic
Nfatc1	-3.706540714	5.00E-61	ncRNA_intronic
P4ha1	-3.705333769	6.38E-22	intergenic
Syngap1	-3.605555277	6.01E-62	ncRNA_intronic
Eva1a	-3.584125584	1E-13	ncRNA_intronic
Scgb1b17	-3.578267782	5.93E-05	intergenic
Gm21645	-3.502500341	0.004765755	intergenic
Gm5796	-3.47912587	1.50E-44	intergenic
Syngap1	-3.462443194	2.25E-117	ncRNA_intronic
Inpp4b	-3.401712671	1.88E-125	intergenic
Gm21699	-3.376729802	1.99E-13	intergenic
Vmn2r37	-3.27753398	0.005333929	intergenic
Lrp1b	-3.243454037	0.002344967	ncRNA_intronic
Gm37564	-3.107427571	2.30E-47	ncRNA_intronic
Ywhaq	-2.853787367	1.23E-13	intergenic
Gm37371	-2.807354889	0.006755345	upstream
Tbr1	-2.733998643	1.33E-15	intergenic
Fibin	-2.729352424	0.000503212	intergenic
Tbr1	-2.70570914	6.33E-18	intergenic
Ctag2	-2.675838934	2.09E-25	intergenic
Kctd16	-2.661504056	0.003483593	intergenic
Klf4	-2.649154807	7.32E-92	intergenic
Ceacam3	-2.600112927	2.04E-07	intergenic
Plxna2	-2.595053421	1E-11	ncRNA_exonic
Nos1	-2.518458095	7.78E-162	ncRNA_intronic
Xlr5a	-2.514026599	0.003180533	ncRNA_intronic
Sbspon	-2.495120793	0.000979253	intergenic
Fam3c	-2.484746464	0.001318026	intergenic
Plxna2	-2.478125399	1E-11	ncRNA_intronic
Runx1t1	-2.402734039	2.32E-39	intergenic
Nos1	-2.387318337	3.01E-142	ncRNA_intronic
Nos1	-2.3624263	1.39E-160	ncRNA_intronic
Abca8b	-2.333068643	0.001620567	intergenic
Plxna2	-2.28755598	1E-11	ncRNA_exonic
Gm21761	-2.272023179	3.63E-05	intergenic
Gm5640	-2.236891991	0.000341133	ncRNA_intronic
Cdh12	-2.205675026	0.009297906	intergenic
Nos1	-2.182961018	3.67E-144	ncRNA_intronic
Pag1	-2.170934422	6.16E-05	intergenic
Plxna2	-2.157039629	1E-11	ncRNA_intronic
Qrfpr	-2.121872004	3.82E-26	intergenic
Gm10147	-2.060473862	2.17E-05	intergenic
Nkx2-4	-2.057715502	0.000213684	intergenic
RP24-334B8.20	-2.040641998	0.000453942	intergenic
Slc35f1	-1.987320866	0.002895693	ncRNA_intronic
Gm10139	-1.98719221	0.0019278	intergenic
D630023O14Rik	-1.955012545	0.001915673	intergenic
Gja1	-1.918027362	5.79E-06	intergenic
Obox7	-1.828911065	0.007704503	intergenic
Lce1d	-1.745771492	2.50E-07	intergenic
RP24-267O6.1	-1.742420029	0.008828072	ncRNA_exonic
Syne1	-1.727179036	3.68E-06	ncRNA_intronic
Tdpoz5	-1.619519723	0.005176686	ncRNA_exonic
4933402D24Rik	-1.569020957	0.007640747	intergenic
Idi1	-1.533338789	3.92E-44	intergenic
Gm21608	-1.532239391	0.000123485	intergenic
Fbn2	-1.513235744	5.62E-15	intergenic
Lrrc4c	-1.480501439	4.84E-13	ncRNA_intronic
Psg20	-1.472426485	8.99E-05	ncRNA_intronic
B4galt5	-1.454985709	5.88E-19	intergenic
Podxl	-1.450948442	8.98E-27	intergenic
Lrp1b	-1.442297272	0.009893232	ncRNA_intronic
Pgrmc1	-1.413138927	3.97E-59	upstream
Rbbp7	-1.381124938	3.03E-13	upstream
Gpc4	-1.379390429	1.09E-13	ncRNA_exonic
Bet1	-1.367509141	5.44E-06	intergenic
Eri1	-1.365845214	0.002308285	intergenic
Cdh10	-1.360747344	0.002717908	intergenic
Astn2	-1.354349569	0.005746336	intergenic
Ywhaq	-1.349661488	0.00197287	intergenic
Ywhaq	-1.349661488	0.00197287	intergenic
St6galnac1	-1.347270939	0.001251423	ncRNA_intronic
Tenm1	-1.323876367	0.001611079	intergenic
Akr1c14	-1.298684606	0.001021967	ncRNA_intronic
Cdh2	-1.278859378	2.14E-05	intergenic
1700015G11Rik	-1.273672247	1.03E-05	intergenic
Alk	-1.270863085	1.61E-05	ncRNA_intronic
Fam71e2	-1.255898214	2.58E-10	ncRNA_intronic
Nudt10	-1.249718567	3.91E-05	ncRNA_exonic
Gm37825	-1.221904098	2.71E-09	intergenic
Kcnn2	-1.203671013	3.28E-14	ncRNA_intronic
Gm20388	-1.173410616	0.00448148	ncRNA_intronic
Immp2l	-1.170772901	1.81E-08	ncRNA_intronic
Snd1	-1.135184019	1.77E-09	ncRNA_intronic
Vmn2r120	-1.098187988	3.86E-08	intergenic
Trim14	-1.09114437	7.62E-56	ncRNA_exonic
Tas2r134	-1.082391542	3.02E-39	intergenic
Cdc42bpb	-1.071083098	0.000171091	intergenic
Poteg	-1.052780728	2.52E-09	intergenic
Hcn1	-1.041914879	4.45E-53	upstream
Prdm6	-1.029051898	4.48E-08	upstream
Nudt10	-1.025938214	8.23E-05	ncRNA_exonic
Map3k15	-1.017684148	1.75E-39	upstream
Snd1	-1.01612741	6.65E-08	ncRNA_intronic
Gm3252	1.002810378	1.58E-11	upstream
Jag2	1.006002091	1.19E-101	ncRNA_exonic
Kcnd3os	1.007576196	2.33E-42	ncRNA_intronic
Jarid2	1.009706958	2.08E-265	ncRNA_intronic
Foxn3	1.011275568	2.73E-97	intergenic

Gm8797	1.014227904	1E-11	intergenic
Zic2	1.017446096	1.74E-40	upstream
Ndrg4	1.018273594	1.28E-71	ncRNA_intronic
Onecut2	1.018567452	2.97E-46	upstream
Auts2	1.019253919	2.57E-30	upstream
Celsr1	1.020897921	9.17E-64	ncRNA_exonic
Iqsec1	1.023475738	3.99E-21	intergenic
6030419C18Rik	1.023657922	2.94E-23	upstream
Foxn3	1.024093096	1.97E-199	intergenic
Jph1	1.025181223	4.70E-42	ncRNA_exonic
Ttc34	1.027350706	6.31E-116	upstream
Fbn2	1.028126888	2.03E-19	ncRNA_exonic
Necab2	1.028682168	1.19E-62	ncRNA_exonic
Nol4l	1.028842323	3.77E-13	ncRNA_exonic
Foxn3	1.030943066	1.00E-10	intergenic
Foxp2	1.032498577	1.39E-19	upstream
Ptprt	1.032861417	5.81E-47	ncRNA_intronic
Iqsec1	1.033063403	1.24E-19	intergenic
Ttc34	1.033087405	5.79E-104	upstream
Dlgap4	1.034130965	2.37E-52	upstream
Cadm1	1.037547541	3.90E-12	ncRNA_intronic
Eid2	1.038686039	4.68E-77	upstream
Foxn3	1.040011252	1.00E-10	intergenic
Slc2a13	1.040336588	2.38E-68	ncRNA_intronic
Gm2238	1.042032693	3.83E-35	ncRNA_exonic
Gm2238	1.045400142	4.81E-45	ncRNA_exonic
Fgfr2	1.049432472	6.92E-56	ncRNA_intronic
Gprasp1	1.053439379	1.30E-16	ncRNA_intronic
Neto1	1.053711497	2.90E-28	upstream
Fignl2	1.055328987	8.11E-08	intergenic
Elmo1	1.05792395	1.31E-38	upstream
Bnc2	1.061613886	5.04E-28	ncRNA_intronic
Cacna2d3	1.062833899	1.04E-51	upstream
Zfp536	1.066125401	9.74E-12	ncRNA_intronic
Brsk2	1.071809406	1.61E-70	upstream
Dab1	1.072565014	1.32E-33	ncRNA_intronic
Adgrb1	1.080518078	3.03E-80	ncRNA_intronic
Ttc34	1.084050714	1.07E-83	upstream
Tpk1	1.084333908	1.44E-05	intergenic
Erbb4	1.089900711	3.16E-63	ncRNA_intronic
Acyp2	1.090983814	1.57E-12	intergenic
Grid1	1.093617632	1.70E-65	upstream
Samd5	1.097905615	3.22E-57	ncRNA_exonic
Fbn2	1.09836931	6.03E-19	ncRNA_exonic
Cadps2	1.098850193	1.95E-61	ncRNA_intronic
Enah	1.099803681	3.75E-35	ncRNA_intronic
Arhgap20	1.099808256	7.65E-62	upstream
Grid1	1.100919323	9.54E-54	upstream
Sall3	1.101479382	4.51E-109	ncRNA_intronic
Cebpa	1.10509527	2.48E-52	upstream
Fzd8	1.105821521	1.21E-73	ncRNA_exonic
Nol4l	1.109743298	1.41E-20	ncRNA_intronic
Auts2	1.110145842	1.88E-50	upstream
Ptprm	1.111947416	8.20E-72	ncRNA_intronic
Nkx6-2	1.119266325	5.32E-81	ncRNA_intronic
Xk	1.122884373	6.34E-17	upstream
Sall3	1.124065264	2.90E-62	ncRNA_intronic
Sgpp2	1.131291808	2.05E-43	upstream
Foxq1	1.136563062	2.22E-66	ncRNA_exonic
Cdh2	1.137923522	2.28E-48	ncRNA_intronic
V1rd19	1.153545258	0.005347362	intergenic
Foxl2	1.153748959	3.45E-33	ncRNA_exonic
Ppm1h	1.160276629	1.49E-61	upstream
Kit	1.163332605	1.02E-42	upstream
Necab2	1.168847652	3.23E-07	ncRNA_intronic
Grid1	1.169427247	3.86E-50	upstream
Fgfr2	1.174989555	1.19E-53	ncRNA_intronic
Rora	1.178213365	1.53E-45	upstream
Eid2	1.178492283	4.04E-77	upstream
Ptprm	1.194630593	1.75E-66	ncRNA_exonic
Nbl1	1.199515725	1.08E-60	ncRNA_intronic
Plxnd1	1.201471997	2.32E-113	ncRNA_intronic
Mc2r	1.20423295	2.24E-219	intergenic
Tbx2	1.205817126	2.80E-31	upstream
Kcnq4	1.207115085	3.17E-76	ncRNA_intronic
Alkbh5	1.211451559	3.70E-05	intergenic
Gm2030	1.211908287	0.006437206	ncRNA_intronic
Stox2	1.212678104	3.02E-75	ncRNA_exonic
Camk2n1	1.219376316	3.09E-27	upstream
Rragd	1.226416573	6.35E-21	upstream
1700008P02Rik	1.23628497	0.000266145	intergenic
4933412E24Rik	1.239960771	0.00273385	intergenic
Satb1	1.247123551	2.81E-73	ncRNA_exonic
1700095A21Rik	1.24726709	9.89E-14	intergenic
Shisa7	1.267508748	1.21E-47	ncRNA_exonic
Rnf157	1.26884742	0.001581862	ncRNA_intronic
Shcbp1	1.269657357	0.003927351	intergenic
Fam214a	1.275251618	2.23E-53	upstream
Igkv4-58	1.281556151	3.63E-80	intergenic
Dcaf13	1.30836556	5.02E-13	intergenic
Gnao1	1.312657222	1.98E-11	ncRNA_intronic
Necab2	1.315031514	1.04E-54	upstream;downstream
Ebf3	1.328688358	3.09E-34	intergenic
Vmn2r37	1.344736145	0.00348062	intergenic
Adgrb1	1.349550355	4.65E-28	upstream
Slc7a2	1.354648452	1.81E-85	upstream
Cadm1	1.370527656	2.91E-79	ncRNA_intronic
Cacnb4	1.375760357	6.51E-05	ncRNA_exonic
Kcnq4	1.407612613	8.86E-112	ncRNA_intronic
Rec114	1.446299499	1.16E-114	intergenic
Sox13	1.471733622	1.66E-82	ncRNA_intronic
Igkv4-58	1.496385258	3.99E-111	intergenic
Nsd1	1.514472983	0.0046802	ncRNA_intronic
Dusp22	1.526385921	1.46E-58	upstream
2810459M11Rik	1.527600323	4.91E-105	upstream
Mterf1b	1.532126253	8.49E-07	intergenic
Ick	1.537990219	3.78E-88	intergenic
Adgrb1	1.547468918	1.23E-35	upstream
1700095A21Rik	1.558351817	7.41E-15	intergenic
Asph	1.560087832	8.58E-05	intergenic
Gm15319	1.569020941	4.85E-07	intergenic
Tmem117	1.626515635	3.46E-39	upstream
Alk	1.647084218	4.13E-06	ncRNA_intronic
Bmp4	1.662177535	7.17E-73	ncRNA_intronic
Cabp1	1.742826592	1.26E-114	ncRNA_intronic
Cabp1	1.798889814	5.75E-152	ncRNA_intronic
Pop4	1.845594597	6.05E-11	intergenic

Gm5784	1.892391027	0.003821498	upstream
Igf2bp3	1.991028467	2.15E-37	ncRNA_exonic
Krtap11-1	2.222392421	0.009020037	intergenic
Tbc1d9	2.226827385	1.08E-18	ncRNA_intronic
Brinp1	2.244887059	1.01E-06	intergenic
Mc2r	2.294970712	1.41E-75	intergenic
Tmem121	2.428236997	0.001109918	intergenic
Tmem121	2.428236997	0.001109918	intergenic
Olfr1507	2.466600305	2.60E-07	intergenic
Olfr1354	2.537656786	0.000360903	intergenic
Wdfy3	2.563385419	2.24E-10	intergenic
Gm3993	2.831276269	1.31E-09	intergenic
Gm3558	2.886570638	6.73E-36	ncRNA_intronic
Gm11554	2.901762717	4.77E-07	upstream
Mid1	2.92201733	9.50E-05	ncRNA_intronic
Ick	2.956019154	2.84E-287	intergenic
E030030I06Rik	3.049696961	7.32E-29	ncRNA_intronic
Gm3383	3.059034433	1.02E-08	ncRNA_intronic
Ick	3.071910967	5.61E-200	intergenic
Gm20388	3.301169548	8.17E-06	ncRNA_intronic
Zfp640	3.332914846	2.40E-30	intergenic
Gnl3l	3.528711481	1.85E-36	intergenic
Ezh2	4.807354811	1.24E-06	intergenic
Gm21800	4.978127975	1.75E-08	intergenic
Ighv1-25	5.018872185	3.23E-09	downstream
4930447F04Rik	5.30762038	6.55E-12	intergenic

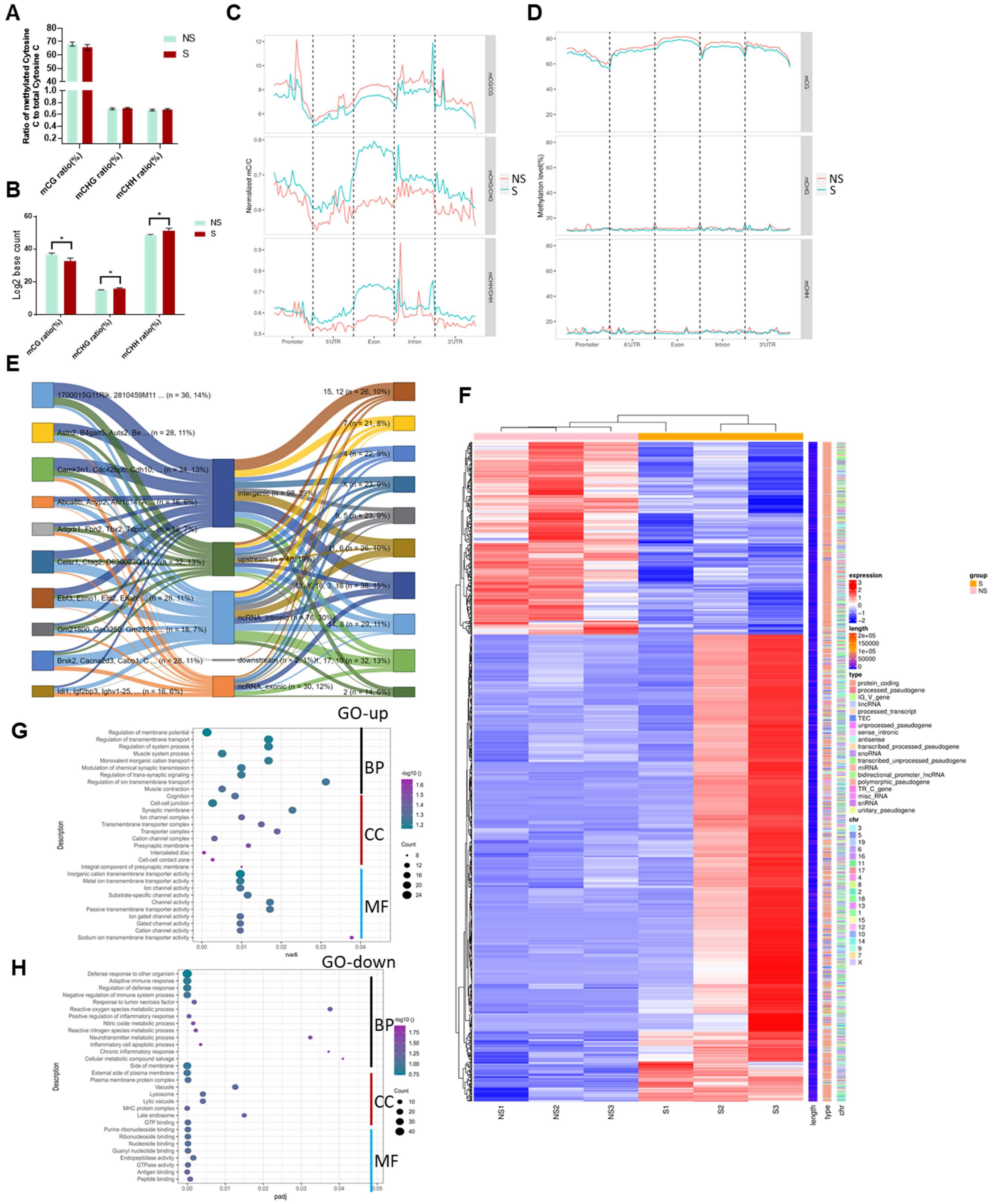

Figure S4. Stress affects the epigenetic signature and gene expression changes in breast tumors. (A) Percentage of methylated cytosines in each context between the NS and S groups. **(B)** The number of each DNA methylation context in NS and S groups. **(C)** Normalized mCs in different genomic regions or elements. The vertical axis on the left represents the normalized mC/C in the two groups. **(D)** Distribution of DNA methylation levels in different genomic functional regions between NS and S groups. Different functional regions were divided into 20 segments, then methylation density and average methylation rate were calculated for each segment. **(E)** Sankey diagram presenting the DMG-genomic region-chromosome stream. The height of each vertical block represents the total number of relevant DMGs. The DMGs located in the same genomic regions, and chromosomes are linked by streams across the vertical blocks. **(F)** Hierarchical Clustering Heatmap. The overall results of FPKM cluster analysis, clustered using the log2(FPKM+1) value. The red color indicates genes with high expression levels in the stressed group sample, and the blue color indicates genes with low expression levels in the stressed group sample. The color ranging from red to blue indicates that log2(FPKM+1) values wherefrom large to small. **(G, H)** Gene ontology analysis of unregaled DEGs and downregulated DEGs in stressed groups. Biological process (BP); Cellular component (CC); Molecular function (MF). One-way analyses of variance (ANOVA) were used to determine the inter-group differences between two groups for one or two variables (*p < 0.05).

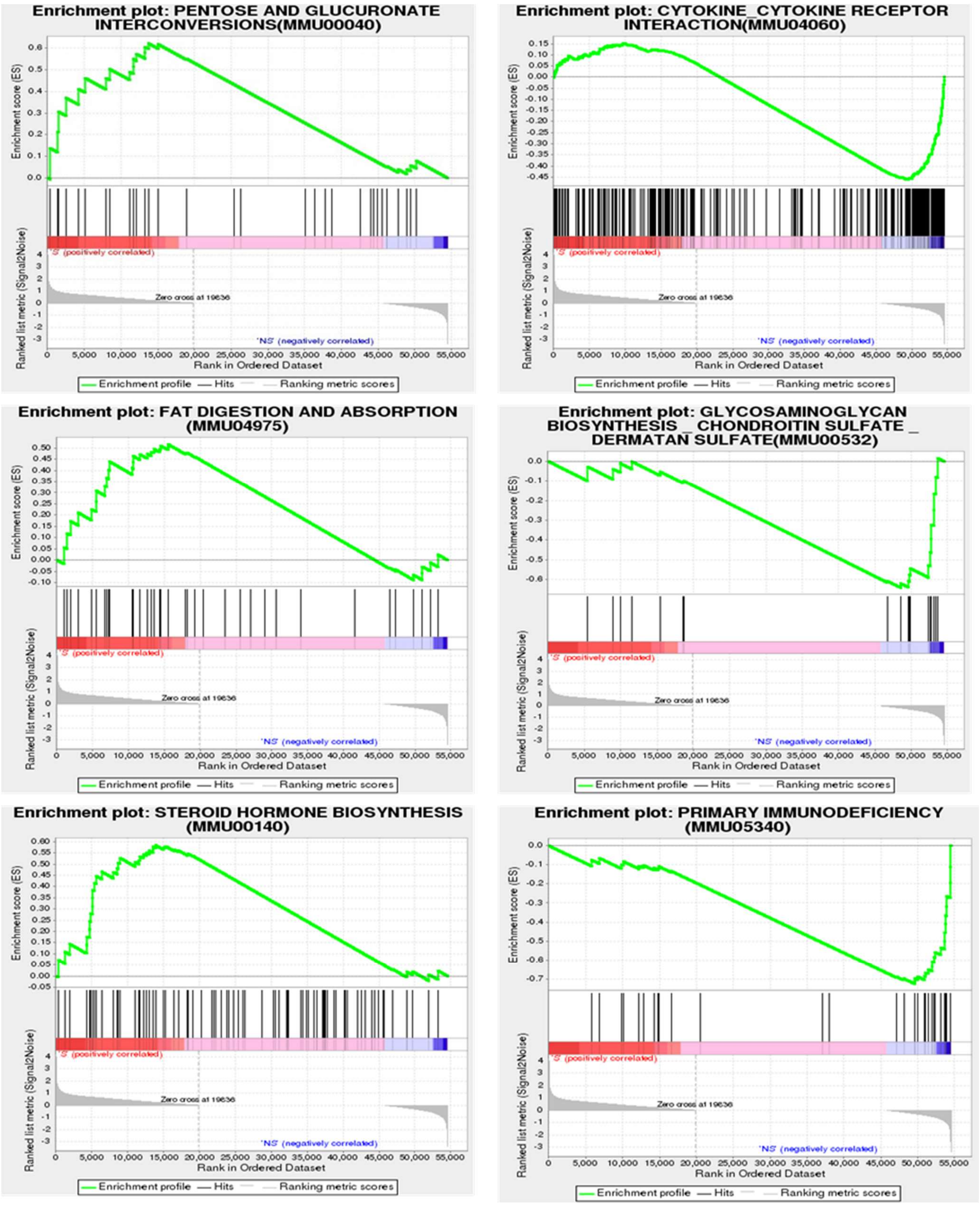

Figure S5. Gene set enrichment analysis (GSEA) was performed using the whole gene list generated from up- and down-regulated DEGs. This whole gene list was pre-ranked based on T-score, then uploaded to GSEA software

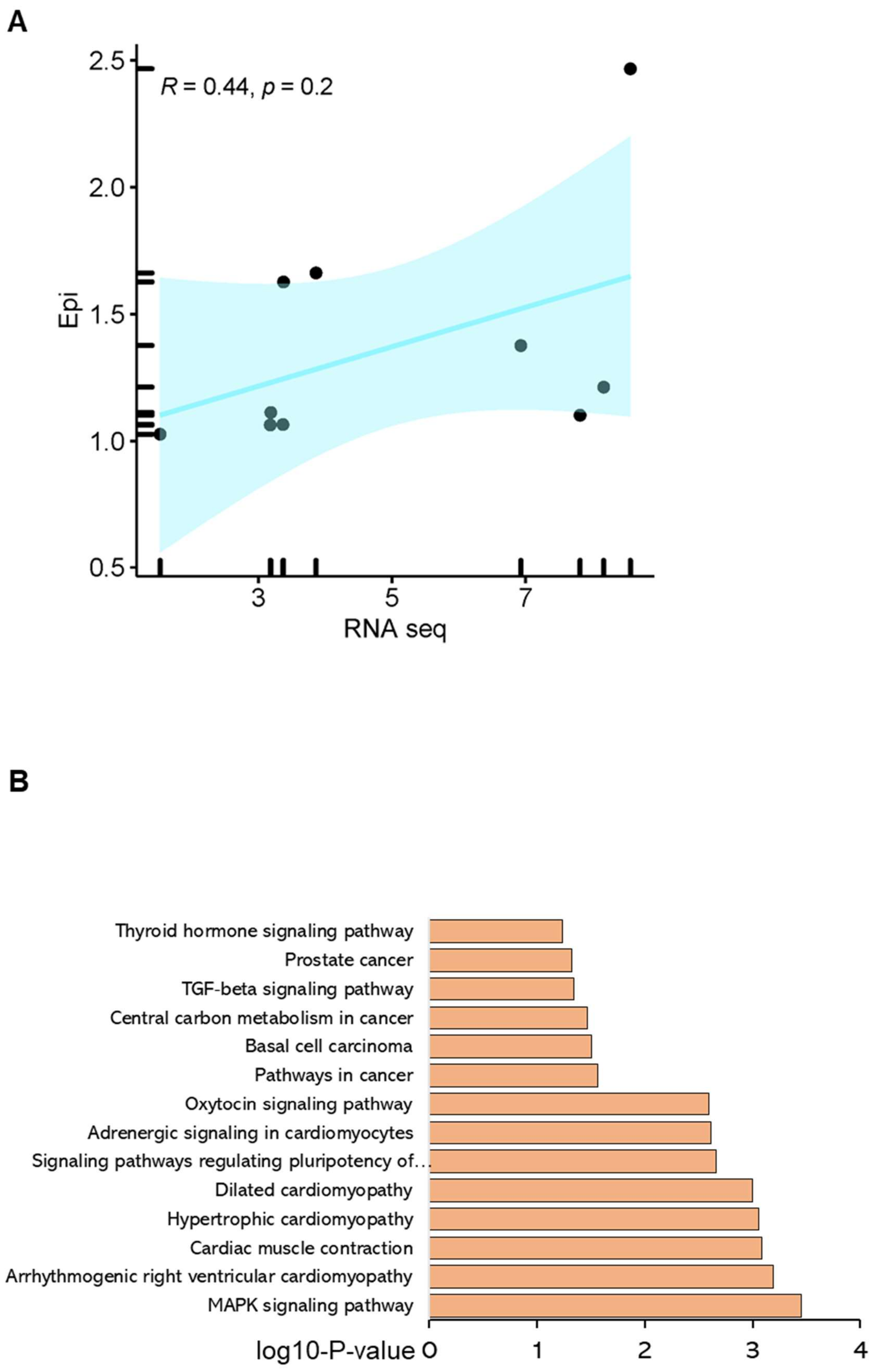

Figure S6. G1 genes Pearson correlation and KEGG analysis. (A) Pearson correlation analysis of G1 genes; R = −1, p = 0.027. Epi and RNAseq stand for the fold change in given genes in stressed samples (R = 0.44, p = 0.2). **(B)** KEGG pathway analysis of G1 genes. The horizontal axis represents the log10 P-value. The vertical axis represents the different KEGG pathways. KEGG, Kyoto encyclopedia of genes and genomes.

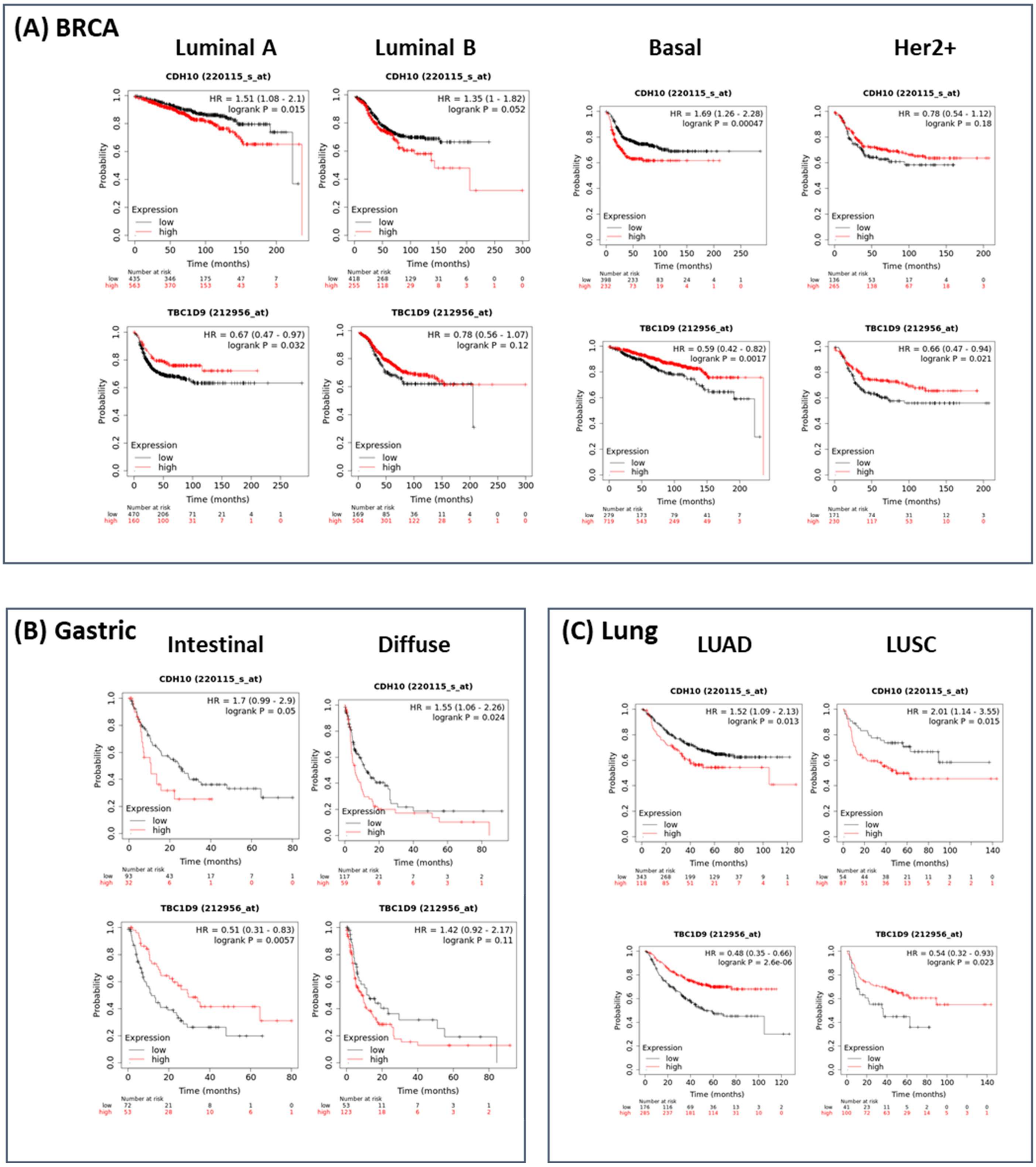

Figure S7. Kaplan-Meier survival curves in different breast (**A**), Gastric (**B**) and lung cancer (**C**) types with low and high expression level of CDH10 and TBC1D9.

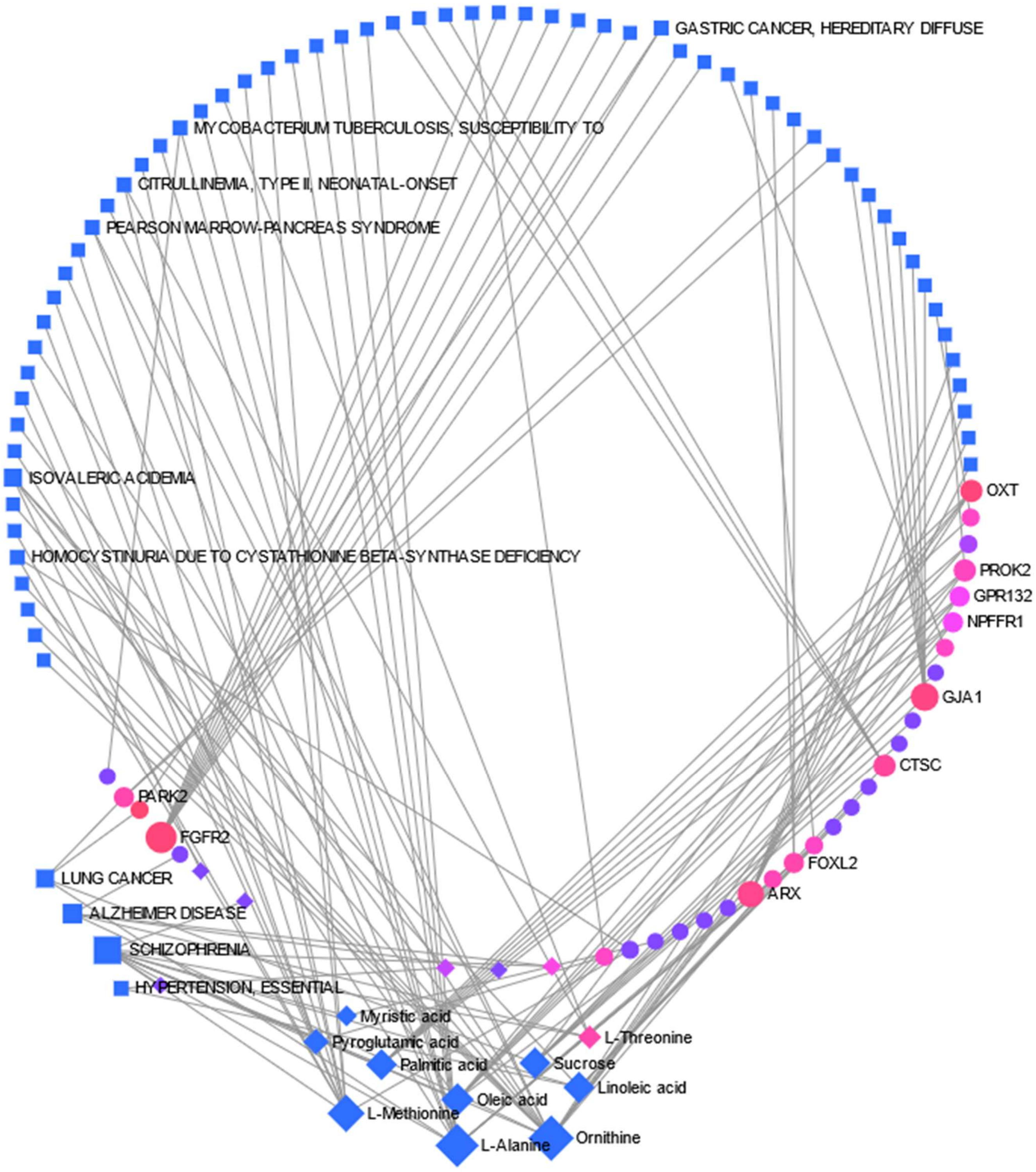

Figure S8. Metabolite-Gene-Disease Interaction Network. Metabolite-gene-disease interaction network analysis using selected metabolites, DEGs, and DMGs. Metabolites are represented by blue diamonds, genes by circles, and related diseases by blue squares. The correlation network is composed of 20 metabolite compounds combined with 31 genes and other relevant diseases. Metabolites with KEGG annotations from the merged data set were mapped to KEGG reference pathways, and interaction networks were generated in MetaboAnalyst5.0 ($p < 0.005$).

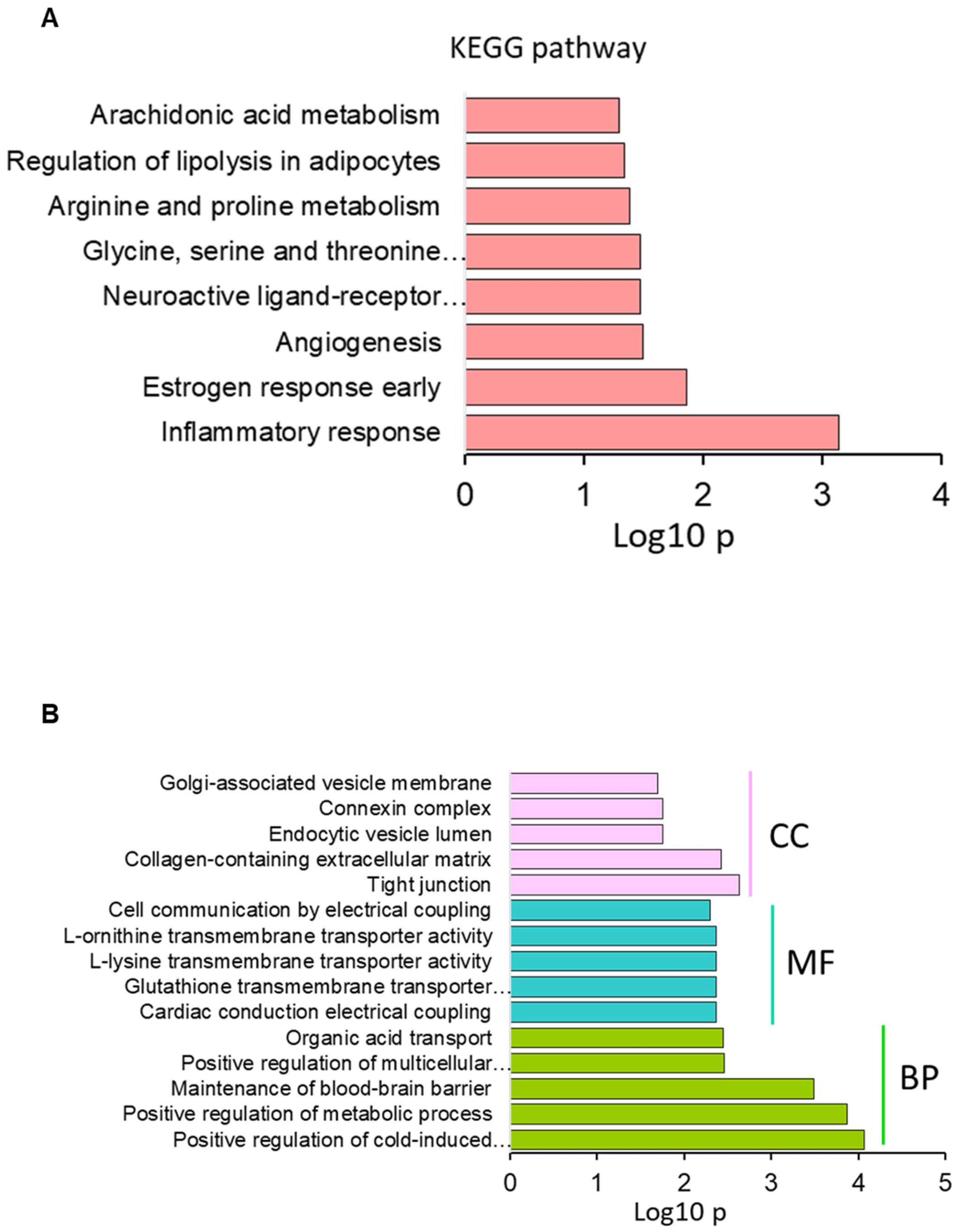

Figure S9. KEGG (A) and gene ontology (B) analysis of 17 mapped genes from Figure. 6F ($p < 0.05$).

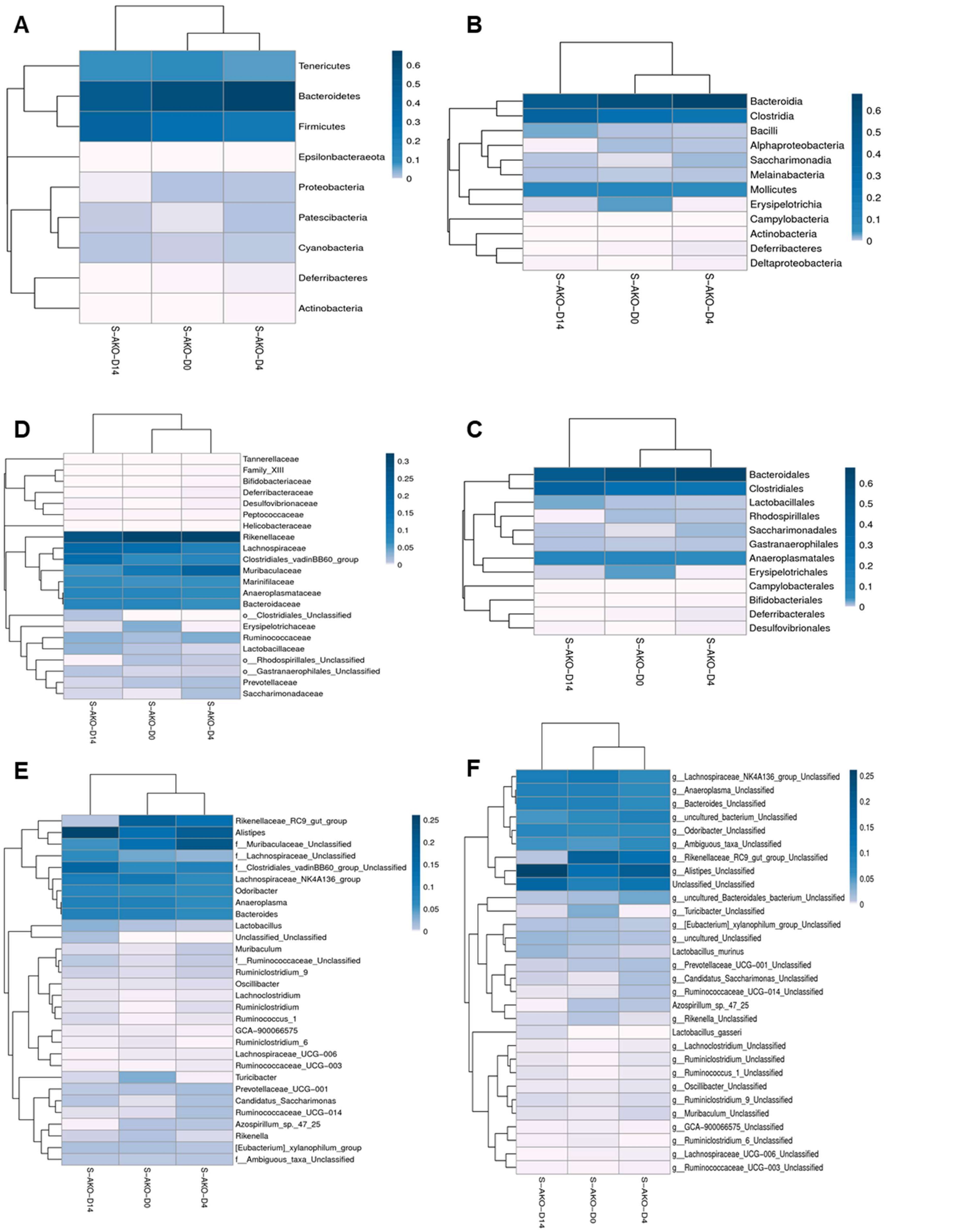

Figure S10. ANXA1$^{-/-}$ alters gut microbiome under stress exposure. The changes in the fecal microbiota after stress were explored using the Mann-Whitney U test at different taxon levels (A-F, at the phylum, class, order, family, genus, and species levels).

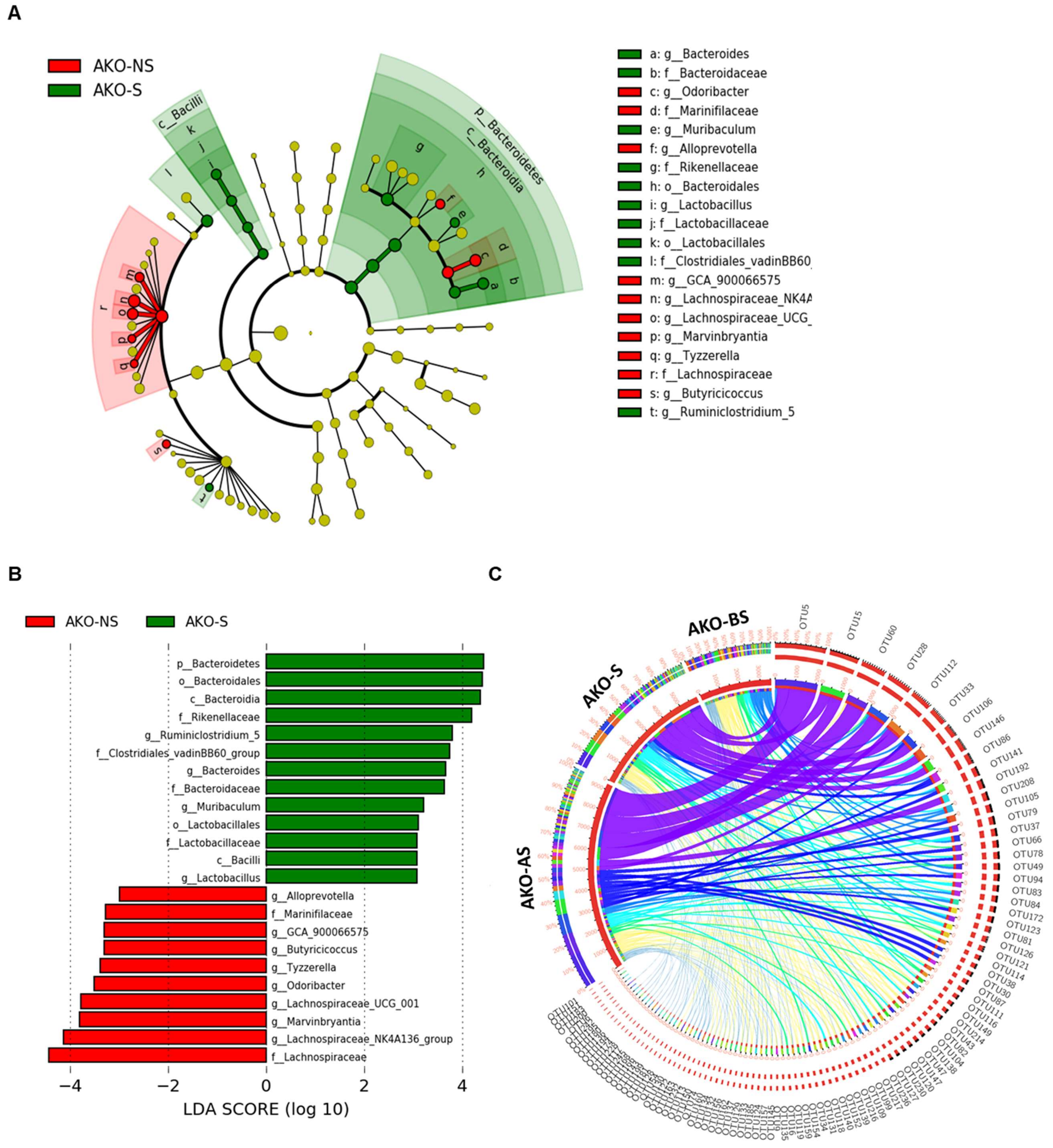

Figure S11 ANXA1 deficiency alters gut microbiome structure under stress (A) LEfSe identified the most differentially abundant taxa between NS controls and S ANXA1$^{-/-}$ mice. Taxonomic cladogram obtained from LEfSe analysis of 16S sequences (relative abundance ⩾ 0.5%). (Red) taxa enriched in NS samples; (Green) taxa enriched in S samples. The brightness of each dot is proportional to the effect size. **(B)** ANXA1$^{-/-}$ stress-enriched taxa are indicated with a positive LDA score (green), and taxa enriched in non-stress have a negative score (red). Only taxa meeting an LDA significant threshold >2 are shown. **(C)** Cladogram depicting the bacterial OTUs detected in the ANXA1$^{-/-}$ mice's fecal microbiota. Branch colors reflect the different OTUs. The intensity and width of the outer ring reflect the average relative abundance of each OTU across all stress timepoints.

Table S8. OTU distribution pattern in fecal microbiome of stressed ANXA1$^{-/-}$ mice

	OTU_ID	taxonomy
ANXA1$^{-/-}$ -D4 and D14 shared	OTU112	p__Patescibacteria;c__Saccharimonadia;o__Saccharimonadales;f__Saccharimonadaceae;g__Candidatus_Saccharimonas;s__uncultured_bacterium
	OTU15	p__Firmicutes;c__Clostridia;o__Clostridiales;f__Lachnospiraceae;g__Lachnospiraceae_NK4A136_group;s__uncultured_bacterium
	OTU28	p__Firmicutes;c__Clostridia;o__Clostridiales;f__Lachnospiraceae
	OTU33	p__Bacteroidetes;c__Bacteroidia;o__Bacteroidales;f__Muribaculaceae;g__uncultured_bacterium;s__uncultured_bacterium
	OTU5	p__Bacteroidetes;c__Bacteroidia;o__Bacteroidales;f__Rikenellaceae;g__Alistipes;s__uncultured_bacterium
	OTU60	p__Firmicutes;c__Clostridia;o__Clostridiales;f__Clostridiales_vadinBB60_group;g__Ambiguous_taxa;s__Ambiguous_taxa
	OTU106	p__Firmicutes;c__Clostridia;o__Clostridiales;f__Ruminococcaceae;g__Ruminococcaceae_UCG-014
	OTU146	p__Bacteroidetes;c__Bacteroidia;o__Bacteroidales;f__Muribaculaceae
ANXA1$^{-/-}$ -D14 Unique	OTU141	p__Firmicutes;c__Clostridia;o__Clostridiales;f__Lachnospiraceae;g__Lachnospiraceae_NK4A136_group
	OTU30	p__Firmicutes;c__Clostridia;o__Clostridiales;f__Clostridiales_vadinBB60_group;g__uncultured_bacterium;s__uncultured_bacterium
	OTU38	p__Firmicutes;c__Clostridia;o__Clostridiales;f__Clostridiales_vadinBB60_group;g__Ambiguous_taxa;s__Ambiguous_taxa
	OTU192	p__Firmicutes;c__Clostridia;o__Clostridiales

Table S9. Microbial compositional changes in ANXA1$^{-/-}$ mice in relation to different diseases

	Disease	Organism	Qualitative outcome[a]	Response	Method
**Saccharimonadia**	Bacterial vaginosis	*Veillonella*	u	Log (ng target DNA/µg total DNA)	qPCR
	Colorectal cancer	*Bacteroides*	u	Log10	qPCR
	Obesity	*Bacteroides*	d	Log10	qPCR
	Periodontal disease	*Pseudoramibacter alactolyticus*	u	Log10	MiSeq sequencing
	Type 2 diabetes	*Faecalibacterium prausnitzii*	d	Log10	qPCR
**Lachnospiraceae**	Sjögren syndrome	*Stomatobaculum*	d	% (prevalence)	16S rRNA sequencing
	Atopic eczema	*Coprococcus eutactus*	d	% (abundancy)	
	Cirrhosis	*Blautia*	d	Median abundancy (%)	16S rRNA pyrosequencing
	Short bowel syndrome	*Blautia*	d	% (abundancy)	MiSeq sequencing
	Non-alcoholic fatty liver disease	*Coprococcus*	d	% (abundancy)	qPCR
	Sjögren syndrome	*Moryella*	d	% (abundancy)	16S rRNA sequencing
	Clostridium difficile infection	*Anaerostipes*	u	% (abundancy)	16S rRNA sequencing
	Type 2 diabetes	*Lachnobacterium*	d	% (abundancy)	16S rRNA sequencing
**Bacteroidales**	Autism	*Bacteroides coprocola*	d	% (abundancy)	bTEFAP
	Autoimmune polyendocrine syndrome type 1	*Bacteroides*	d	% (abundancy)	16S rRNA sequencing
	Cirrhosis	*Rikenellaceae*	d	Median abundancy (%)	16S rRNA pyrosequencing
	Colorectal cancer	*Prevotella copri*	d	% (abundancy)	16S rRNA sequencing
	Constipation	*Odoribacter*	d	% (abundancy)	16S rRNA sequencing
	Cystic fibrosis	*Bacteroides intestinalis*	d	% (abundancy)	bTEFAP
	Obesity	*Bacteroides*	d	Log10	qPCR
	Oral cancer	*Porphyromonas gingivalis*	d	% (prevalence)	16S rRNA sequencing
	Pervasive developmental disorder NOS	*Prevotella copri*	d	% (abundancy)	bTEFAP
	Pouchitis	*Parabacteroides*	d	Number of identifiable sequences	16S rRNA sequencing

[a] u: Elevated d: Reduced

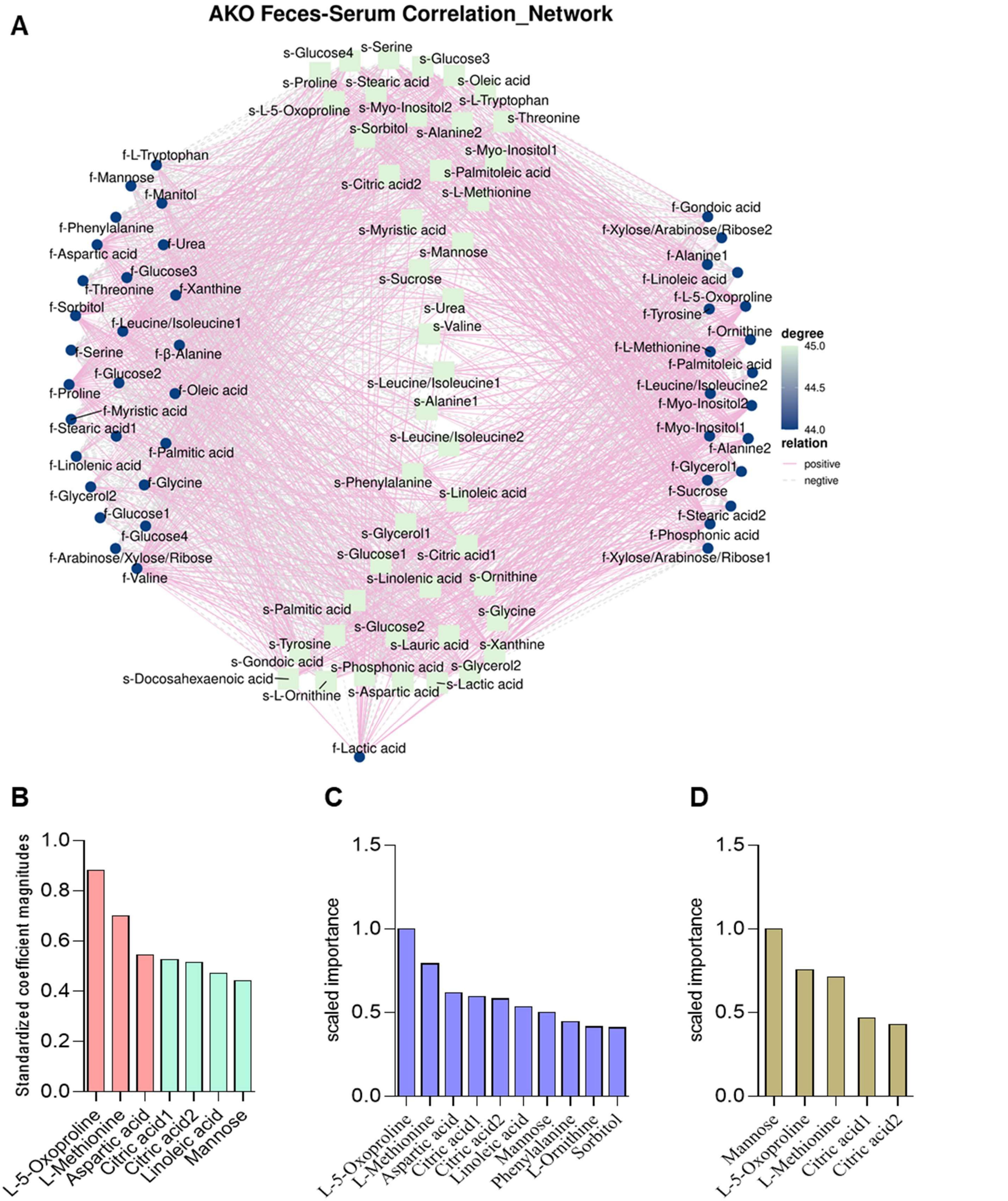

Figure S12. Correlation analysis of ANXA1$^{-/-}$ feces and serum metabolites. (A) Net map to display the correlations of fecal and serum metabolites in stressed ANXA1$^{-/-}$ mice. **(B-D)** Variable importance of metabolites from serum samples from stressed ANXA1$^{-/-}$ mice was analyzed using three machine learning approaches. From left to right, GLM, GBM, and DRF with 3-fold cross-validation; training AUC = 1 and testing AUC = 1.

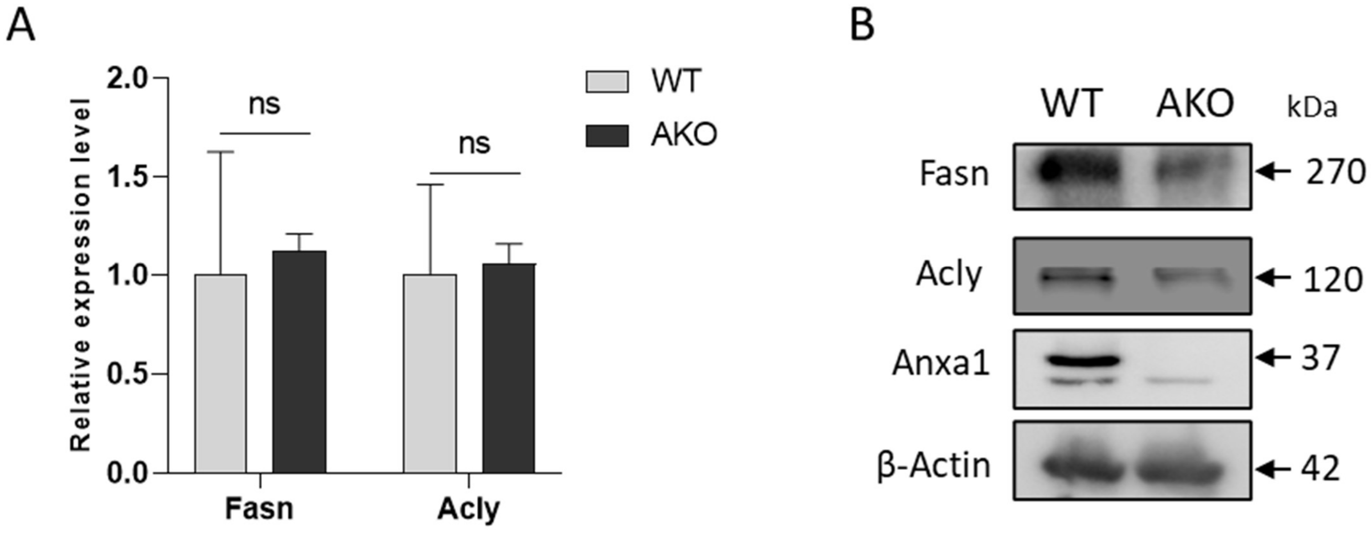

Figure S13. ANXA1 deficiency influences fatty acid synthesis. (A) Real-time PCR analysis was performed using cDNA prepared from 4T1 WT and Anxa1 -/- cells. The data shown are representative of three independent experiments and expressed as the mean ± SD. ns>0.05, paired t-test. **(B)** Western blot analysis of fatty acid synthesis protein expression in 4T1 WT and Anxa1 -/- cells. Anti-β-Actin antibody for loading control. AKO, Anxa1 -/- cells.

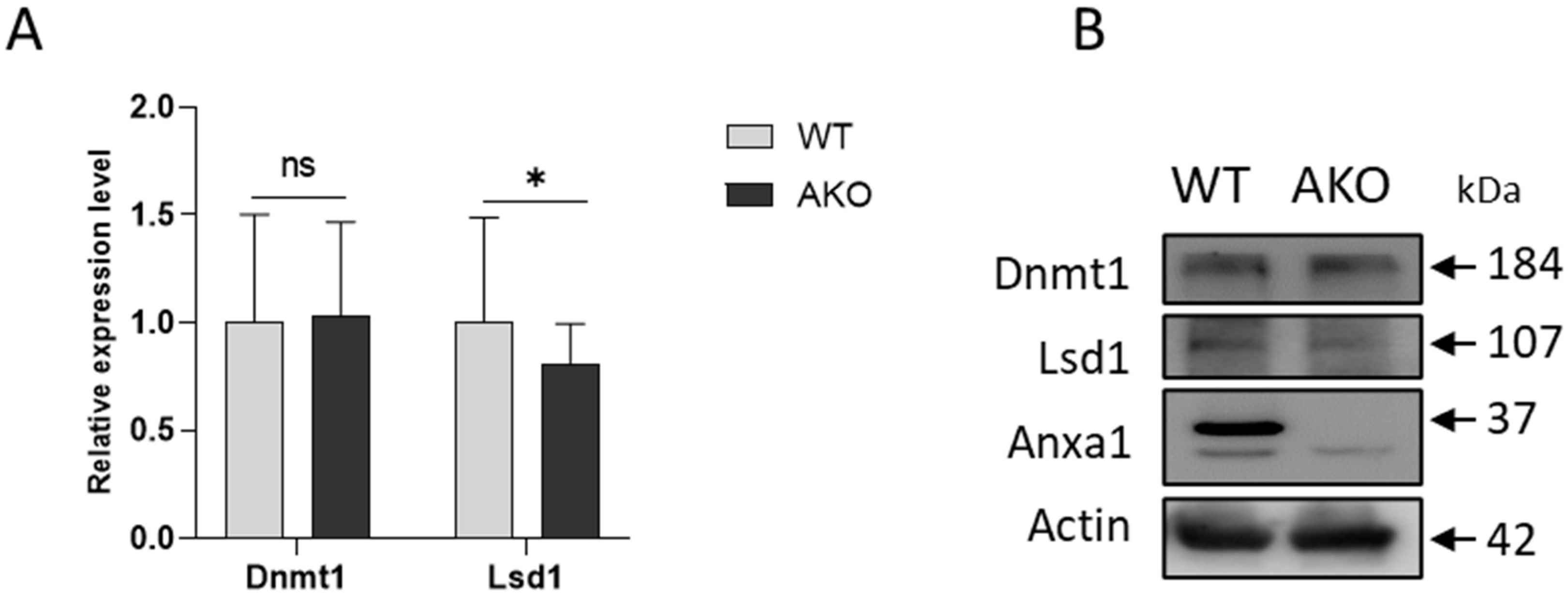

Figure S14. ANXA1 deficiency influences DNA methylation and demethylation. (A) Real-time PCR analysis was performed using cDNA prepared from 4T1 WT and Anxa1 -/- cells. The data shown are representative of three independent experiments and expressed as the mean ± SD. * p<0.05, paired t-test. **(B)** Western blot analysis of methylation and demethylation protein expression in 4T1 WT and Anxa1 -/- cells. Anti-β-Actin antibody for loading control. AKO, Anxa1 -/- cells.

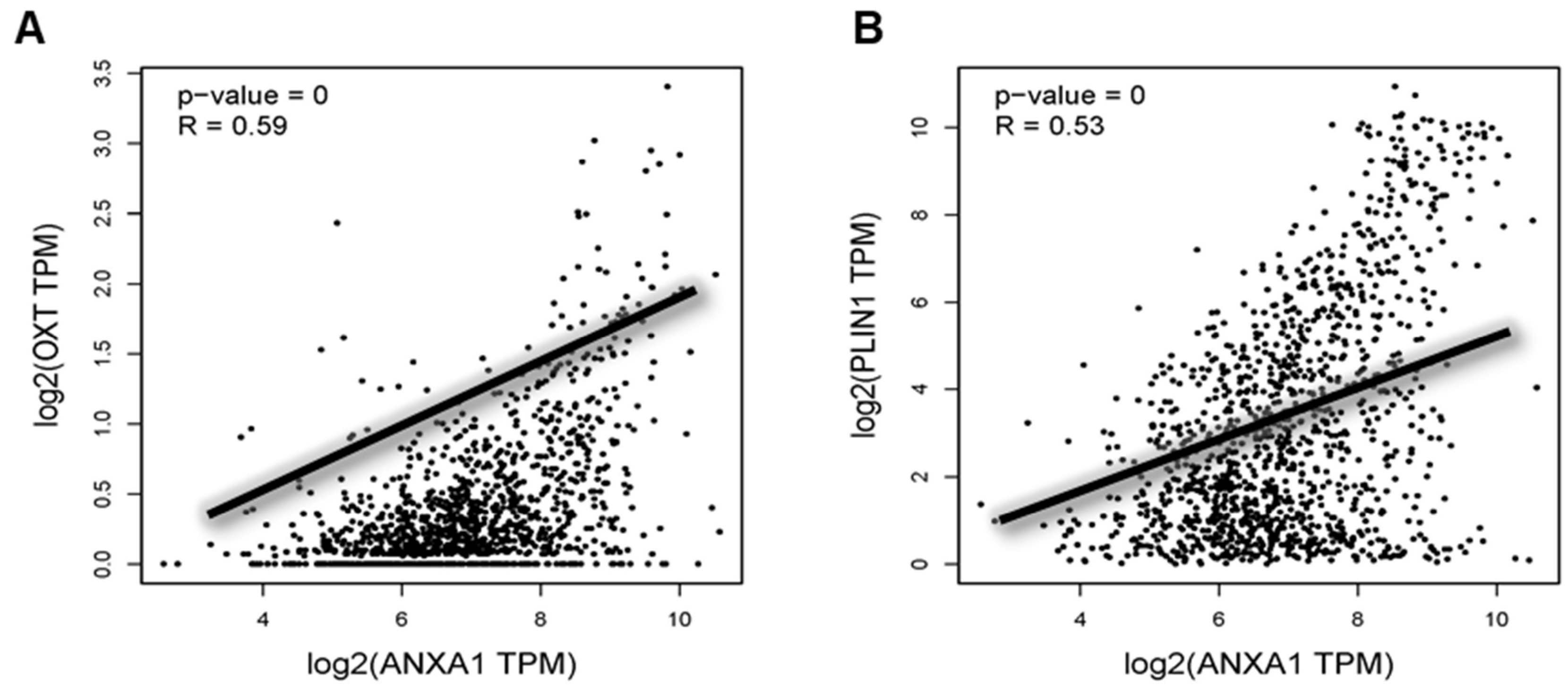

Figure S15 Pair-wise gene expression correlation analysis for Anxa1 and selected hub genes (OXT and PLIN1) using breast cancer TCGA and GTEx expression data in GEPIA.
